# Evaluation of Daily Low-Dose Prednisolone During Upper Respiratory Tract Infection to Prevent Relapse in Children With Relapsing Steroid-Sensitive Nephrotic Syndrome: The PREDNOS 2 Randomized Clinical Trial

**DOI:** 10.1001/jamapediatrics.2021.5189

**Published:** 2021-12-20

**Authors:** Martin T. Christian, Nicholas J. A. Webb, Samir Mehta, Rebecca L. Woolley, Nafsika Afentou, Emma Frew, Elizabeth A. Brettell, Adam R. Khan, David V. Milford, Detlef Bockenhauer, Moin A. Saleem, Angela S. Hall, Ania Koziell, Heather Maxwell, Shivaram Hegde, Hitesh Prajapati, Rodney D. Gilbert, Caroline Jones, Karl McKeever, Wendy Cook, Natalie Ives

**Affiliations:** 1 Department of Paediatric Nephrology, Nottingham Children’s Hospital, Nottingham, UK; 2 Department of Paediatric Nephrology, University of Manchester, Manchester Academic Health Science Centre, Royal Manchester Children’s Hospital, Manchester, UK; 3 Birmingham Clinical Trials Unit, University of Birmingham, Birmingham, UK; 4 Health Economics Unit, University of Birmingham, Birmingham, UK; 5 Department of Paediatric Nephrology, Birmingham Children’s Hospital, Birmingham, UK; 6 Department of Renal Medicine, University College London, London, UK; 7 Department of Paediatric Nephrology, Great Ormond Street Hospital for Children, London, UK; 8 Department of Glomerular Cell Biology, Bristol Medical School, University of Bristol, Bristol, UK; 9 Department of Paediatric Nephrology, Bristol Royal Hospital for Children, Bristol, UK; 10 Leicester Children’s Hospital, Leicester, UK; 11 Child Health Clinical Academic Group, King’s College London, London, UK; 12 Department of Paediatric Nephrology, Evelina Children’s Hospital, London, UK; 13 Department of Paediatric Nephrology, Royal Hospital for Sick Children, Glasgow, UK; 14 Department of Paediatric Nephrology, University Hospital of Wales, Cardiff, UK; 15 Department of Paediatric Nephrology, Leeds Children’s Hospital, Leeds, UK; 16 Department of Paediatric Nephrology, Southampton Children’s Hospital, Southampton, UK; 17 Department of Paediatric Nephrology, Alder Hey Children’s Hospital, Liverpool, UK; 18 Department of Paediatric Nephrology, Royal Hospital for Sick Children, Belfast, UK; 19 Nephrotic Syndrome Trust, Taunton, UK

## Abstract

**Importance:**

In children with corticosteroid-sensitive nephrotic syndrome, many relapses are triggered by upper respiratory tract infections. Four small studies found that administration of daily low-dose prednisolone for 5 to 7 days at the time of an upper respiratory tract infection reduced the risk of relapse, but the generalizability of their findings is limited by location of the studies and selection of study population.

**Objective:**

To investigate the use of daily low-dose prednisolone for the treatment of upper respiratory tract infection–related relapses.

**Design, Setting, and Participants:**

This double-blind, placebo-controlled randomized clinical trial (Prednisolone in Nephrotic Syndrome [PREDNOS] 2) evaluated 365 children with relapsing steroid-sensitive nephrotic syndrome with and without background immunosuppressive treatment at 122 pediatric departments in the UK from February 1, 2013, to January 31, 2020. Data from the modified intention-to-treat population were analyzed from July 1, 2020, to December 31, 2020.

**Interventions:**

At the start of an upper respiratory tract infection, children received 6 days of prednisolone, 15 mg/m^2^ daily, or matching placebo preparation. Those already taking alternate-day prednisolone rounded their daily dose using trial medication to the equivalent of 15 mg/m^2^ daily or their alternate-day dose, whichever was greater.

**Main Outcomes and Measures:**

The primary outcome was the incidence of first upper respiratory tract infection–related relapse. Secondary outcomes included overall rate of relapse, changes in background immunosuppressive treatment, cumulative dose of prednisolone, rates of serious adverse events, incidence of corticosteroid adverse effects, and quality of life.

**Results:**

The modified intention-to-treat analysis population comprised 271 children (mean [SD] age, 7.6 [3.5] years; 174 [64.2%] male), with 134 in the prednisolone arm and 137 in the placebo arm. The number of patients experiencing an upper respiratory tract infection–related relapse was 56 of 131 (42.7%) in the prednisolone arm and 58 of 131 (44.3%) in the placebo arm (adjusted risk difference, −0.02; 95% CI, −0.14 to 0.10; *P* = .70). No evidence was found that the treatment effect differed according to background immunosuppressive treatment. No significant differences were found in secondary outcomes between the treatment arms. A post hoc subgroup analysis assessing the primary outcome in 54 children of South Asian ethnicity (risk ratio, 0.66; 95% CI, 0.40-1.10) vs 208 children of other ethnicity (risk ratio, 1.11; 95% CI, 0.81-1.54) found no difference in efficacy of intervention in those of South Asian ethnicity (test for interaction *P* = .09).

**Conclusions and Relevance:**

The results of PREDNOS 2 suggest that administering 6 days of daily low-dose prednisolone at the time of an upper respiratory tract infection does not reduce the risk of relapse of nephrotic syndrome in children in the UK. Further work is needed to investigate interethnic differences in treatment response.

**Trial Registration:**

isrctn.org Identifier: ISRCTN10900733; EudraCT 2012-003476-39

## Key Points

**Question:**

In children with relapsing corticosteroid-sensitive nephrotic syndrome, does daily low-dose corticosteroid given at the time of upper respiratory tract infection prevent upper respiratory tract infection–related relapse?

**Findings:**

In this randomized clinical trial of 365 children, daily low-dose corticosteroid therapy resulted in no difference in the frequency of upper respiratory tract infection–related relapses compared with placebo.

**Meaning:**

Results of this study suggest that low-dose corticosteroid treatment is not beneficial as a strategy to prevent upper respiratory tract infection–related relapses.

## Teaser

This randomized clinical trial investigates whether a low daily dose of prednisolone in children with relapsing steroid-sensitive nephrotic syndrome prevents upper respiratory tract infection–related relapse.

## Introduction

Steroid-sensitive nephrotic syndrome (SSNS) is the most common glomerular disease of childhood, with an incidence of approximately 2 per 100 000 children, although it is up to 6 times more common in children of South Asian ethnic origin.^1^ At least 80% of children with SSNS will relapse,^2^ and relapses are associated with a risk of significant complications, including sepsis, thrombosis, dyslipidemia, and malnutrition.^3^ At least 50% of relapses follow intercurrent infections, most commonly upper respiratory tract infections (URTIs)^4,5,6^; furthermore, in children with relapsing SSNS, half or more URTIs will trigger a relapse.^7^

Previous research^8,9,10,11^ in Saudi Arabia, Sri Lanka, and India has suggested that giving low-dose daily prednisolone for 5 to 7 days when a URTI is diagnosed reduces the risk of an ensuing relapse. The first 3 study populations^8,9,10^ were restricted to children already taking maintenance alternate-day prednisolone in which the dose given every 48 hours was converted to daily for 5 to 7 days. Only the Sri Lankan study^9^ was a double-blind, placebo-controlled randomized clinical trial. That group later reproduced their findings in a population of children receiving no maintenance prednisolone treatment.^11^ These studies were relatively^8,9,10,11^ small (36-100 participants). Only the Sri Lankan studies^9,11^ were blinded, and a number of potential sources of bias were identified in Cochrane meta-analyses,^12^ including crossover design (where the first treatment may have influenced the second), postrandomization exclusions, and paucity of corticosteroid adverse effect reporting. The generalizability of the results is limited by the effect on SSNS of climate-specific patterns of infectious disease, the ethnicity of the patients, and the predominance of children receiving maintenance alternate-day corticosteroid treatment. The study by Gulati et al^10^ included a subpopulation of children receiving background levamisole in addition to prednisolone; no studies have evaluated the efficacy of the intervention in children receiving other noncorticosteroid background therapy.

The primary objective of this trial was to undertake a well-powered and methodologically robust study to determine whether, in a population of children of predominantly European and Asian ethnicity who were living in a temperate climate and had relapsing nephrotic syndrome while receiving different background medication or no medication, a 6-day course of low-dose daily prednisolone reduced the incidence of URTI-related relapse (URR). The secondary objectives were to compare the overall rate of relapses, the incidence of escalations or reductions in background therapy, the cumulative dose of prednisolone during 12 months, and assessment of corticosteroid adverse effects, including behavior, quality of life (QoL), and treatment costs. In line with a previous multicenter randomized clinical trial^2,13^ of the duration of prednisolone to treat the initial episode of nephrotic syndrome, we incorporated a trial-based economic evaluation that will be reported in full elsewhere.

## Methods

Prednisolone in Nephrotic Syndrome (PREDNOS) 2 was a phase 3, parallel-group, placebo-controlled, double-blind randomized clinical trial to test the efficacy of daily low-dose prednisolone given at the time of a URTI in reducing the risk of URR. The trial protocol^14^ was conceived and developed with input from the Nephrotic Syndrome Trust, the main UK patient support group for nephrotic syndrome. The approved trial protocol is available in Supplement 1. The trial was approved by the UK National Research Ethics Service Committee North West–Greater Manchester Central on December 4, 2012. Written informed consent was obtained from parents after provision of detailed written information about the trial. Written informed assent was obtained from older children whom the research team deemed competent. Trial oversight was provided by an independent trial steering committee that convened twice yearly and an independent data monitoring committee that reviewed interim efficacy and safety data. This study followed the Consolidated Standards of Reporting Trials (CONSORT) reporting guideline.

The trial was performed from February 1, 2013, to January 31, 2020, in 122 UK pediatric departments, consisting of 13 specialized pediatric nephrology and 109 general pediatric units because in the UK most children with SSNS are cared for by general pediatricians and only those with a more complicated disease course are seen by pediatric nephrologists. Children 1 to 18 years of age with SSNS and 2 or more relapses in the preceding 12 months were included in the study. Those with steroid-resistant nephrotic syndrome, those receiving or within 3 months of completing a course of cyclophosphamide or rituximab, those receiving daily prednisolone therapy, and those taking an alternate-day prednisolone dose greater than 15 mg/m^2^ were excluded.

### Trial Population

On the basis of a risk of a URTI resulting in a relapse in 50% of instances,^6,7^ to detect an absolute difference of 17.5% (ie, 35% proportional reduction) in URR rate (ie, from 50% to 32.5%), with 80% power and α = .05, a sample size of 250 children was required. To allow for a 15% attrition rate, the sample size was increased to 300 children (150 in each arm). During the trial, it became apparent that a larger number of participants than expected (28%) were completing the 12-month trial without experiencing a URTI. The sample size was therefore increased to 360 patients based on a revised attrition rate of 30%, which would provide the 250 patients needed to detect the difference as per the original sample size calculation.

A total of 365 children (182 in the prednisolone arm and 183 in the placebo arm) with relapsing SSNS were recruited from 91 of the 122 UK pediatric centers that were set up. Eighty children completed 12 months of follow-up without experiencing a URTI. Consent was withdrawn for 32 (8.8%), of whom 14 were withdrawn before having a URTI. This left a modified intention-to-treat population of 271 children (134 in the prednisolone arm and 137 in the placebo arm). Patient flow according to CONSORT guidelines is shown in the Figure.

**Figure.**
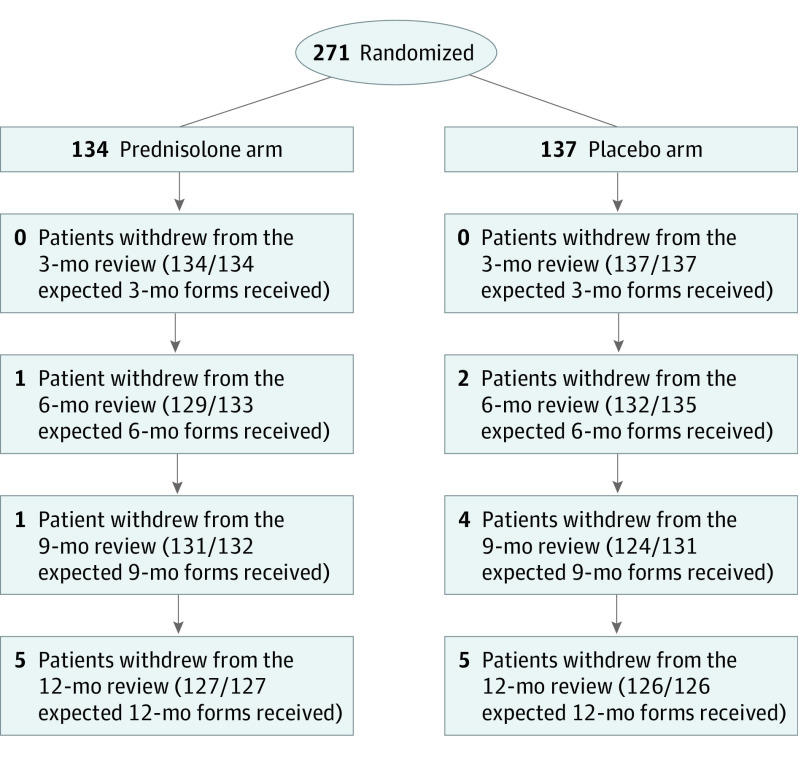
CONSORT Diagram for the Modified Intention-to-Treat Population

### Trial Procedures and Interventional Medicinal Product

Information sheets that outlined the trial were mailed to the parents or guardians of potentially eligible children (and the child when age appropriate) 1 to 2 weeks before their next clinical appointment. After confirmation of eligibility with regard to the inclusion and exclusion criteria and a further full discussion of the trial, informed consent was sought from the parents (or guardians) and children (informed consent or assent according to age) at the time of this appointment.

Children were randomized in a 1:1 ratio, minimized by background therapy at recruitment (no background treatment, maintenance prednisolone only, maintenance prednisolone and noncorticosteroid immunosuppression, and noncorticosteroid immunosuppression only), using a secure 24-hour internet-based randomization service or by a telephone call to the University of Birmingham Clinical Trials Unit. After randomization, a central pharmacy posted trial medication to the families that comprised 5-mg prednisolone tablets or matching placebo tablets. Both families and the clinical investigation teams were blinded to the allocation of the trial medication.

At the time of randomization, families were given verbal and written instructions on the number of tablets to take at the time of a URTI. For children not receiving background prednisolone treatment, the intervention arm received a daily dose of 15 mg/m^2^ (maximum dose, 40 mg) and the placebo arm an equivalent number of identical placebo tablets. The daily dose of prednisolone for those patients receiving background prednisolone treatment at the time of the URTI was the larger of 15 mg/m^2^ daily or their current alternate-day dose: for example, a child with a body surface area of 1.0 m^2^ taking background prednisolone of 5 mg on alternate days at the time of the URTI needed to take 2 study tablets and 5 mg of prednisolone on usual prednisolone days and 3 study tablets on nonprednisolone days; a child with a body surface area of 1.0 m^2^ receiving background prednisolone treatment of 20 mg on alternate days at the time of the URTI needed to take 20 mg of prednisolone on usual prednisolone days and 4 study tablets on nonprednisolone days.

A URTI was defined as the presence of at least 2 of the following for at least 24 hours: sore throat, ear pain or discharge, runny nose, cough, hoarse voice, or temperature higher than 37 °C. Parents were provided with written information as well as a fridge magnet aide-memoire for this definition and a tympanometric electronic thermometer to accurately record their child’s temperature. They were asked to contact their local research team shortly after commencing use of trial medication.

Parents tested their child’s urine daily as is routine practice. A relapse was defined as urine dipstick proteinuria (≥3+) for 3 consecutive days or the presence of generalized edema plus proteinuria (3+). A URR was defined as a relapse that occurred within 14 days of the development of a URTI.

Children were followed up for 12 months from randomization. At each 3-monthly hospital outpatient visit, children were examined clinically. Information on relapses, medication, and specific corticosteroid adverse effects was collected. Parents also completed an Achenbach Child Behavioral Checklist (ACBC).^15^ Data on QoL were collected at these outpatient visits using the Pediatric Quality of Life Inventory^16,17^ and the Child Health Utility Index–9 Dimension or EuroQoL 5-Dimension^18^ instruments (dependent on the child’s age).

If the child relapsed at any point during the trial, including after a URTI, the child was advised to undergo treatment with a standard relapse course,^19^ stopping use of trial medication if necessary. Relapses were based on a standard definition of proteinuria (3+) for 3 days based on home testing and were not confirmed by a hospital visit. Parents were allowed to commence treatment for relapse without consulting with their medical teams if they were confident to do this.

Patients underwent intensification of background immunosuppressive therapy (ie, the addition of a new immunosuppressive agent) after 2 or more relapses in any 6-month period or when unacceptable adverse effects of prednisolone or other therapy occurred. Similarly, immunosuppressive therapy was discontinued after sustained remission for at least 6 months or when unacceptable adverse effects of therapy occurred.

Adherence to the trial medication was assessed at each follow-up visit by asking whether the parent began administering the trial medication to their child at the time of the URTI, the number of days from the start of the URTI to the start of medication use, and whether the whole 6-day course of trial mediation was taken or prematurely discontinued.

### Trial Outcomes

The primary outcome was the incidence of the first URR after any URTI during the 12-month follow-up period. Secondary outcomes were the overall rate of URRs, overall rate of relapses (URTI and non–URTI related), escalations or reductions in background treatment, cumulative dose of prednisolone during 12 months, rates of serious adverse events, incidence of corticosteroid adverse effects, change in behavior measured by ACBC, and QoL.

### Statistical Analysis

Analyses were based on the intention-to-treat principle. The analysis population was based on a modified intention-to-treat population, which included only those children who had a URTI during the 12-month follow-up period. Regression models provided estimates of treatment effects along with 2-sided 95% CIs and *P* values from 2-sided tests at the *P* < .05 significance level. Analyses were adjusted for the minimization variable (background therapy at randomization) and baseline scores (where appropriate). No corrections for multiple tests were made. All analyses were performed using SAS statistical software, version 9.4 (SAS Institute Inc) or Stata software, version 16 (StataCorp). Subgroup analyses were limited to the primary outcome only. Data from the modified intention-to-treat population were analyzed from July 1, 2020, to December 31, 2020.

## Results

### Trial Population

After withdrawals or completion of follow-up without an URTI, the modified intention-to-treat analysis population comprised 271 children (mean [SD] age, 7.6 [3.5] years; 174 [64.2%] male), with 134 in the prednisolone arm and 137 in the placebo arm. The baseline demographic characteristics for the modified intention-to-treat population are given in Table 1. For the modified intention-to-treat population at all 4 follow-up data collection time points, 1040 of a possible 1055 case report forms (98.6%) that contained study visit data were received (99.0% from the intervention arm and 98.1% from the placebo arm).

**Table 1.** Baseline Demographic Characteristics for the Modified Intention-to-Treat Population^a^

Characteristic	Prednisolone (n = 134)	Placebo (n = 137)
Background treatment regimen^b^		
No long-term treatment	31 (23.1)	31 (22.6)
Long-term maintenance prednisolone	40 (30.0)	34 (24.8)
Other immunosuppressant therapy plus long-term maintenance prednisolone	43 (32.1)	48 (35.0)
Other immunosuppressant therapy only	20 (14.9)	24 (17.5)
Age, mean (SD), y	7.7 (3.6)	7.5 (3.5)
Sex		
Male	83 (61.9)	91 (66.4)
Female	51 (38.0)	46 (33.6)
BMI percentile		
Median (IQR)	84.1 (63.7-96.9)	86.4 (68.4-97.0)
Underweight (<5th percentile)	0	0
Healthy (5th-84th percentile)	69 (51.5)	64 (46.7)
Overweight (85th-94th percentile)	24 (17.9)	30 (21.9)
Obese (≥95th percentile)	41 (30.6)	43 (31.4)
Prednisolone dose on alternate days, mg	9.2 (3.7)	8.4 (3.1)
Race and ethnicity^c^		
South Asian	30 (22.4)	28 (20.4)
White	96 (71.6)	92 (67.2)
Other or unknown	8 (6.0)	17 (12.4)
Age at diagnosis of nephrotic syndrome, mean (SD), y	4.4 (2.5)	4.4 (2.8)
Time to randomization, median (IQR) [range], d		
Time to last relapse	90 (58-143) [14-280]	87 (58-126) [7-280]
Second last relapse	209.5 (153-287) [42-363]	189 (146-252) [36-365]

Abbreviation: BMI, body mass index (calculated as weight in kilograms divided by height in meters squared).

^a^ Data are presented as number (percentage) of patients unless otherwise indicated.

^b^ Minimization variable.

^c^ Ethnicity was self-reported from an extended list and subsequently rationalized into broad categories as displayed. The broad category definitions according to self-reported ethnicity are given in eTable 5 in Supplement 2.

### Primary Outcome

A total of 384 URTIs and 82 URRs occurred in the prednisolone arm and 407 URTIs and 82 URRs in the placebo arm. A total of 56 of 131 children (42.7%) experienced a URR in the prednisolone arm and 58 of 131 (44.3%) in the placebo arm (adjusted risk difference, −0.02; 95% CI, −0.14 to 0.10; *P* = .70) (Table 2). One patient in the placebo arm had a URTI but no information was provided on whether that patient had a URR or not; therefore, data for this patient were classed as missing. Three patients in the intervention arm and 5 patients in the placebo arm had URTIs but withdrew before the 12-month follow-up and did not report any URR for any time points when they provided data. These patients were excluded from the primary analysis but were included in the sensitivity analysis. Sensitivity analyses for the primary outcome are given in eTable 1 in Supplement 2. There was no evidence that the treatment effect differed when the data were analyzed by predefined subgroups according to background treatment (eFigure in Supplement 2).

**Table 2.** Primary and Secondary Outcome Measures

Outcome measure	Prednisolone (n = 134)	Placebo (n = 137)	Treatment effect (95% CI)^a^	*P* value
**Primary outcome measure**				
Proportion of patients experiencing an URTI-related relapse (n = 131 in both arms)				
No	75 (57.3)	73 (55.7)	RD, −0.024 (−0.142 to 0.095)	.70
Yes	56 (42.7)	58 (44.3)	RD, 0.96 (0.74 to 1.26)	
**Secondary outcome measures**				
URTI-related relapse rate (n = 134 in prednisolone arm and 136 in placebo arm)				
0	78 (58.2)	78 (57.4)	IRR, 0.98 (0.72 to 1.33)	.88
1	36 (26.9)	41 (30.1)		
2	15 (11.2)	10 (7.4)		
3	4 (3.0)	7 (5.1)		
4	1 (0.8)	0 (0)		
Proportion of patients experiencing any relapse (URTI and non–URTI related) (n = 132 in both arms)				
No	41 (31.1)	34 (25.8)	RD, −0.05 (−0.16 to 0.06)	.33
Yes	91 (68.9)	98 (74.2)	RD, 0.93 (0.80 to 1.09)	
Relapse rate (n = 134 in prednisolone arm and 136 in placebo arm)				
0	43 (32.1)	38 (27.9)	IRR, 0.89 (0.74 to 1.07)	.23
1	28 (20.9)	39 (28.7)		
2	24 (17.9)	24 (17.7)		
3	22 (16.4)	11 (8.1)		
4	11 (8.2)	14 (10.3)		
5	6 (4.5)	5 (3.7)		
≥6	0 (0)	5 (3.7)		
Proportion of patients who had escalation of background immunosuppressant therapy (n = 130 in prednisolone arm and 128 in placebo arm)				
No	72 (55.4)	71 (55.5)	RD, −0.003 (−0.12 to 0.12)	.96
Yes	58 (44.6)	57 (44.5)	RD, 0.98 (0.75 to 1.29)	
Proportion of patients who had reduction of background immunosuppressant therapy (n = 128 in prednisolone arm and 129 in placebo arm)				
No	73 (57.0)	67 (51.9)	RD, −0.04 (−0.15 to 0.06)	.42
Yes	55 (43.0)	62 (48.1)	0.91 (0.71 to 1.16)	
Cumulative prednisolone dose, median (IQR), mg	2060 (1128-3355)	1880 (1115-3295)	Difference, 180 (−301.83 to 661.83)	.72

Abbreviations: IRR, incidence rate ratio (an offset was included in the model); RD, risk difference; RR, risk ratio; URTI, upper respiratory tract infection.

^a^ All treatment effects are from regression models that adjusted for the background therapy at baseline, except for cumulative prednisolone dose, which is based on an unadjusted analysis with the *P* value from a Wilcoxon rank sum test. An IRR less than 1 favors the prednisolone arm. An RR less than 1 favors the prednisolone arm. A negative RD favors the prednisolone arm.

In the modified intention-to-treat group, 58 (21.4%) of the population reported South Asian ethnicity (Bangladeshi, Indian, mixed White-Asian, Pakistani, Sri Lankan, or other Asian). A total of 30 of 134 individuals (22.4%) were in the prednisolone arm and 28 of 137 (20.4%) in the placebo arm (see eTable 5 in Supplement 2 for broad classification of ethnic grouping). A post hoc subgroup analysis that assessed the primary outcome in those of South Asian ethnicity (risk ratio, 0.66; 95% CI, 0.40-1.10) vs other (Afro-Caribbean, mixed White and Afro-Caribbean, White, other ethnicity, or not stated) ethnicity (risk ratio, 1.11; 95% CI, 0.81-1.54) found that daily prednisolone was more effective in those of South Asian ethnicity, but the result was nonsignificant (test for interaction *P* = .09).

### Secondary Outcomes

No significant differences in secondary outcomes were found between the treatment arms (Table 3). A total of 216 relapses (URTI and non–URTI related) occurred in 91 children in the prednisolone arm and 237 relapses occurred in 98 children in the placebo arm (adjusted risk difference, −0.05; 95% CI, −0.16 to 0.06; *P* = .33). Background treatment was escalated on at least 1 occasion in 58 of 130 children (44.6%) in the prednisolone arm compared with 57 of 128 children (44.5%) in the placebo arm (*P* = .96). Fifty-five of 128 children (43.0%) in the prednisolone arm had at least 1 treatment reduction during the trial compared with 62 of 129 children (48.1%) in the placebo arm (*P* = .42). The median cumulative dose of prednisolone during 12 months was 2060 mg (IQR, 1128-3355 mg) in the prednisolone arm and 1880 mg (IQR, 1115-3295 mg) in the placebo arm (*P* = .72). No significant differences were found between trial arms in the number of serious adverse events or specific corticosteroid adverse effects (eTable 2 in Supplement 2). No differences were found in behavior scores measured using ACBC, or in QoL (eTables 3 and 4 in Supplement 2).

**Table 3.** Comparison of All Trial Populations

Variable	PREDNOS 2	Mattoo et al^8^	Abeyagunawardena et al,^9^ 2008	Gulati et al^10^	Abeyagunawardena et al,^11^ 2017
Recruited sample size	365	36	48 (Crossover)	100	48 (Crossover)
Completed sample size	253	Not reported	40	89	33
Age at recruitment, mean (SD), y	7.7 (3.6) (Prednisolone arm) and 7.5 (3.5) (placebo arm)	7.2 (Prednisolone arm) and 6.8 (control arm)	Median (range), 5.3 (1.5-13.2)	6.5 (2.97) (Prednisolone arm) and 6.8 (3.23) (control arm)	12.3 (Prednisolone arm) and 9.9 (placebo arm)
Time from diagnosis to recruitment, mo	39.7 for intervention and 36.8 for control	Not reported	Not reported	9.8 for intervention and 10.5 for control	90 For intervention and 76.8 for control
Population definition	≥2 Relapses in past 12 mo	Low-dose maintenance prednisolone: FRNS (n = 22); after cyclophosphamide (n = 14)	Receiving low-dose maintenance prednisolone	Receiving low-dose maintenance prednisolone	Previous SDNS not receiving any immunosuppression for ≥3 mo
Average background prednisolone dose, mg/kg every 48 h	0.3 (maintenance prednisolone) (n = 165)	0.5	0.36 (range, 0.1-0.6)	0.6 (0.1) (Prednisolone arm) and 0.7 (0.2) (placebo arm)	Not reported
Other background immunosuppression	Other immunosuppression alone: n = 44; low-dose prednisolone plus other immunosuppression: n = 91	No	No	Levamisole in 32 of 100 patients	No
No. of infections (per patient per year)	Mean, 2.95; median, 2	3.5^a^	NA	3.8^b^	3.3^c^
Children excluded because of no URTI	80 of 333 who completed 12-mo follow-up (24%)	0	3 of 48 (6.3%)	0	0
Total relapses (per patient per year)	1.67	1.93	Not reported	1.34	0.55
URR frequency in control arm, % of all URTIs	20.2	Not reported	47.5	35.0	24.8

Abbreviations: FRNS, frequently relapsing nephrotic syndrome; NA, not applicable; PREDNOS 2, Prednisolone in Nephrotic Syndrome 2; SDNS, steroid-dependent nephrotic syndrome; URR, URTI-related relapse; URTI, upper respiratory tract infection.

^a^ Median of 7 URTIs reported during 2 years.

^b^ A total of 226 episodes of infections reported in the intervention group and 161 in the control group for population of 100.

^c^ A total of 115 URTIs in the treatment arm and 101 URTIs in the control group with 33 who completed the 2 years of the trial as the denominator.

### Adherence

Trial medication was commenced at the time of a URTI for 691 of 791 URTIs (87.3%): 328 URTIs (85.4%) in the intervention arm and 363 URTIs (89.2%) in the placebo arm. The median time to commencement was 0 days (IQR, 0-1 day) for both arms of the trial. No differences were found in rates of adherence for each 3-month period throughout the trial. The most common reasons for not starting use of the trial medication at the time of the URTI were parent or guardian forgetting, medication had not arrived in time, and child already relapsed and/or treatment for relapse.

To address any effect of adherence on the primary outcome, we performed an exploratory per protocol analysis of those URTIs in which medication use was started within 2 days of the URTI. The analysis found an adjusted risk difference of −0.05 (95% CI, −0.17 to 0.08), supporting the trial’s main finding.

## Discussion

This randomized clinical trial (PREDNOS 2) found that giving 6 days of daily low-dose prednisolone to children with relapsing SSNS at the time of a URTI does not reduce the risk of a URR. The findings are consistent for all background treatment subgroups. Similarly, no differences were found in secondary outcomes between the 2 treatment arms.

The findings do not support the conclusions of 4 previously published trials, which reported a benefit of daily prednisolone therapy at the time of URTI.^8,9,10,11^ However, these previous studies^8,9,10,11^ have methodologic issues, such as lack of blinding, small sample size, postrandomization exclusions, and crossover design. These issues limit the impact of these studies,^8,9,10,11^ leading to the latest Cochrane review concluding that “clinicians are unlikely to use this regimen without additional data to confirm its efficacy and safety.”^12^^(p 20)^

A comparison of the populations for all 5 trials is given in Table 3. The average age at recruitment was similar for the PREDNOS 2 cohort. The overall frequency of relapses during the trial was similar, but the rate of infections was lower for PREDNOS 2. Only 1 trial^9^ of the 4 previous trials reported numbers of children excluded for lack of infection, and this percentage was much smaller (Table 3). Of note, the event rate (the likelihood of any single infection resulting in a relapse in the placebo arm of the trial) was lower in PREDNOS 2, even though the overall rate of relapses was similar. Historical observational studies^4,5,6^ reported an event rate of 50%, but only the first Sri Lankan trial^9^ reported a similar event rate. In their second trial,^11^ the event rate (24.8%) was considerably lower and in PREDNOS 2 was lower still (20.2%).

No comment about ethnicity is made in any of the previous studies,^8,9,10,11^ and it is assumed that the populations comprised local populations of homogeneous ethnicity. In this trial, which recruited children from diverse ethnic backgrounds, the small difference in response according to ethnicity from a post hoc subgroup analysis was interesting. Differences in rates of infection do not wholly explain the differences in the trial findings or even in event rate; intervention response related to ethnicity may provide an important explanation for the difference in our findings compared with previous studies.^8,9,10,11^ However, the numbers are small, and this was not a planned subgroup analysis. Furthermore, ethnic differences in disease response to treatment are not supported by recent cohort studies.^20,21^

### Strengths and Limitations

The strengths of PREDNOS 2 are its size, methods, and multicenter nature. It is also the only trial to have included children receiving all types of background treatment or none. In contrast to some of the previous trials,^8,10^ PREDNOS 2 was double-blinded and placebo-controlled. It was the only trial that systematically recorded corticosteroid adverse events, including an objective measure of the effect of corticosteroid on behavior.

In contrast to previous studies,^8,9,11^ we did not review and examine children at the time of their URTI, and it is possible that some excluded children had experienced URTIs that were not captured within the trial definition. If we had made a type 2 error by failing to find a significant result that is present, we would have expected to see an increased number of relapses. However, the small increase in patients experiencing any relapses in the placebo arm was non-significant.

## Conclusions

In the largest ever clinical trial, to our knowledge, of an investigational medicinal product in children with nephrotic syndrome, for a nonselected population of children with relapsing SSNS, PREDNOS 2 found no benefit to a short course of daily low-dose prednisolone at the time of a URTI. The size of the trial is larger than all previous studies^8,9,10,11^ combined and is therefore likely to change the Cochrane meta-analysis on this issue. Further work is needed to investigate interethnic differences in treatment response and the pathogenesis of individual viral infections and their effect on nephrotic syndrome.

## Notes

Supplement 1. Trial Protocol and Statistical Analysis Plan

Supplement 2. **eTable 1.** Sensitivity Analyses for Primary Outcome Measures **eTable 2.** Adverse Events **eTable 3.** Achenbach Child Behavior Checklist Scores **eTable 4.** Quality of Life Using the PEDSQL **eTable 5.** Rationalisation of Ethnic Groups Following Self-reported Ethnicity **eFigure.** Subgroup Analyses for Primary Outcome Measure

Supplement 3. Data Sharing Statement
