# Supplementary material for: Evaluation of Daily Low-Dose Prednisolone During Upper Respiratory Tract Infection to Prevent Relapse in Children With Relapsing Steroid-Sensitive Nephrotic Syndrome: The PREDNOS 2 Randomized Clinical Trial
Source: JAMA Pediatr. 2021 Dec 20;176(3):1–8. doi: 10.1001/jamapediatrics.2021.5189 (PMC8689426; doi:10.1001/jamapediatrics.2021.5189)

PROTOCOL

Full Title: Short course daily prednisolone therapy at the time of upper respiratory tract infection in children with relapsing steroid sensitive nephrotic syndrome

Short Title: PREDNOS 2 study

EudraCT number – 2012-003476-39
MHRA Clinical Trials Authorisation: 21761/0281/001-0001
Ethical Approval: North West GM Central Ref: 12/NW/0766
ISRCTN10900733

Trial Co-Sponsors – Manchester University NHS Foundation Trust and University of Birmingham
Sponsor's Project Number: RG_12-188

Chief Investigator – Dr Martin Christian
Nottingham Children's Hospital, Nottingham

Co-Investigators – Ms Natalie Ives, Dr Emma Frew, Mrs Elizabeth Brettell
University of Birmingham
Dr David Milford, Prof Detlef Bockenhauer, Prof Moin Saleem, Dr Angela Hall, Dr Ania Koziell, Dr Heather Maxwell, Dr Shivaram Hegde, Dr Eric Finlay, Dr Rodney Gilbert, Ms Jenny Booth, Dr Caroline Jones, Dr Karl McKeever, Mrs Wendy Cook
NIHR Medicines for Children Research Network Nephrology Clinical Studies Group

Protocol Version 3.0 **Date 1st August 2018**

Funding Body: National Institute for Health Research Health Technology Assessment programme: Grant number 11/129/261

Amendments and Chief Investigator Signature

This protocol describes the PREDNOS 2 trial and provides information about procedures for patients taking part in the PREDNOS 2 trial. The protocol should not be used as a guide for treatment of patients not taking part in the PREDNOS 2 trial.

This protocol version has been approved by:

Name: Dr Martin Christian **Trial Role:** Chief Investigator

Signature: [signature] **Date:** 6 August 2018

Date of amendment	Protocol version number	Type of amendment	Summary of amendment
22nd Nov 2012	1.1	In response to REC provisional opinion - minor	Typo corrections and addition of CTA, REC and ISRCTN numbers
20th Feb 2014	1.2	Substantial amendment	Amendment to the wording of an exclusion criterion, additional details added to the statistical analysis section, update to the TSC and DMEC members, and other minor and administrative amendments.
9th June 2015	1.3	Substantial amendment	Update to recruitment period and total study duration.
28th February 2017	2.0	Substantial amendment	Update to recruitment period and study duration, amendment to the wording of SAE reporting, amendment to the primary endpoint, update to the data collection section and other minor amendments.
1st August 2018	3.0	Substantial amendment	Change in Chief Investigator, sponsor trust name change, data protection regulations update, parental responsibility text added and update to recruitment period and total study duration.

Principal Investigator Declaration

I have read and agree to the current PREDNOS 2 protocol, as described above and understand that any suggested changes to the protocol must be approved by the Trial Steering Committee prior to seeking approval from the Main Research Ethics Committee (MREC) and/or Regulatory Authority.

I am aware of my responsibilities as an Investigator under the guidelines of Good Clinical Practice (GCP), the Declaration of Helsinki, local regulations (as applicable) and the trial protocol and I agree to conduct the trial according to these guidelines and to appropriately direct and assist the staff under my control, who will be involved in the trial.

Principal Investigator Signature

Name: ………………………………………………………….

Signature: ……………………………………………………...

Date: ……………………………………………………………

Name of Institution

Trust Name: ……………………………………………………

The Principal Investigator should sign this page, file the original copy with the current protocol in the site file, and return a copy to the PREDNOS 2 Trial Office.

PREDNOS 2 Trial Management Group

Nephrology
Dr Martin Christian
(Nottingham Children's Hospital)
Martin.Christian@nhs.net

Clinical Trials & Statistics
Ms Natalie Ives
(University of Birmingham Clinical Trials Unit)
n.j.ives@bham.ac.uk

Mrs Elizabeth Brettell
(University of Birmingham Clinical Trials Unit)
e.a.brettell@bham.ac.uk

Health Economics
Dr Emma Frew
(University of Birmingham)
e.frew@bham.ac.uk

PREDNOS 2 Study Office

For any general queries about the study and for information on site set-up:

Birmingham Clinical Trials Unit (BCTU), College of Medical & Dental Sciences, Public Health Building, University of Birmingham, Edgbaston, Birmingham B15 2TT, UK

Tel: 0121 415 9100 (answering machine outside office hours); Fax: 0121 415 9135

**Renal Trials Manager**	Mrs Elizabeth Brettell	0121 415 9130	e.a.brettell@bham.ac.uk
**Trial Coordinator**	Miss Emma Barsoum	0121 415 9132	barsoume@bham.ac.uk
**Computing**	Mr Nick Hilken	0121 415 9121	n.h.hilken@bham.ac.uk
**Statistician**	Miss Natalie Ives	0121 415 9113	n.j.ives@bham.ac.uk

Randomisation

Internet: https://www.trials.bham.ac.uk/PREDNOS2
Telephone: 0800 953 0274 (toll free in UK, available 9am to 5pm)

Clinical queries during office hours should be directed to one of the Clinical Co-ordinators, or to an appropriate member of the Trial Management Group.

Protocol Synopsis

Title	Short course daily prednisolone therapy at the time of upper respiratory tract infection in children with relapsing steroid sensitive nephrotic syndrome
Short Title	PREDNOS 2 study
Clinical Phase	III
Chief Investigator	Dr Martin Christian, Nottingham Children's Hospital, Nottingham
Trial Co-Sponsors	Manchester University NHS Foundation Trust and University of Birmingham
Funding	National Institute for Health Research Health Technology Assessment programme (HTA 11/119/261)
Sample Size	360
Accrual Period	5 years and 11 months
Follow-up period	All subjects will be followed up for 12 months
Study Duration	7 years and 10 months
End of Trial Definition	The end of trial will be 6 months after the last data capture. The last data capture will be 12 months following recruitment of the last subject. This will allow sufficient time for the completion of protocol procedures, data collection and data input. The Trials Office will notify the MHRA and main REC that the trial has ended and will provide them with a summary of the clinical trial report within 12 months of the end of trial.
Study Design	Double blind randomised controlled trial (RCT)
Aim of Study	To evaluate the effectiveness of a six day course of daily prednisolone therapy at the time of URTI in reducing the development of subsequent nephrotic syndrome relapse in children with relapsing SSNS.
Primary Study Objectives	To determine whether a six day course of oral prednisolone given at the time of URTI reduces the incidence of first URTI-related relapse in children with relapsing steroid sensitive nephrotic syndrome
Primary Outcome Measures	First URTI-related relapse of nephrotic syndrome during the 12 month follow-up period.<br>Relapse is defined as Albustix positive proteinuria (+++ or greater) for three consecutive days or the presence of generalised oedema plus 3+ proteinuria. URTI-related relapse is defined as a relapse occurring within 14 days of the development of an URTI. First URTI-related relapse refers to the first URTI-related relapse which occurs within the 12 month study follow up period.
Secondary Study	To determine whether a six day course of oral

Objectives	prednisolone given at the time of URTI<br>i] Reduces the overall rate of URTI-related relapse of nephrotic syndrome (expressed as relapses per year).<br>ii] Reduces the overall rate of relapse of nephrotic syndrome (expressed as relapses per year).<br>iii] Reduces the cumulative dose of prednisolone received over the 12 month study period.<br>iv] Reduces the incidence and prevalence of adverse effects of prednisolone including behavioural abnormalities.<br>v] Reduces the number of subjects undergoing escalation of background immunosuppressive therapy (e.g. addition of ciclosporin, tacrolimus, cyclophosphamide etc.).<br>vi] Increases the number of subjects undergoing reduction of background immunosuppressive therapy (e.g. cessation of ciclosporin or long term maintenance prednisolone therapy).<br>vii] Is more cost effective than standard therapy.
Secondary Outcome Measures	i] Rate of URTI-related relapse of nephrotic syndrome (relapses per year).<br>ii] Rate of relapse (URTI-related and non URTI-related) of nephrotic syndrome (relapses per year).<br>iii] Cumulative dose of prednisolone (mg/kg and mg/m^2) received over the 12 month study period.<br>iv] Incidence of SAEs.<br>v] Incidence of adverse effects of prednisolone including assessment of behaviour using the Achenbach Child Behaviour Checklist.<br>vi] Incidence of escalation of background immunosuppressive therapy (e.g. addition of ciclosporin, tacrolimus, cyclophosphamide etc.).<br>vii] Incidence of reduction of background immunosuppressive therapy (i.e. cessation of long term maintenance prednisolone therapy).<br>viii] Quality of life using the CHU-9D, EQ-5D and PedsQL.<br>ix] Cost per relapse of nephrotic syndrome.<br>x] Cost per QALY gained.
Inclusion Criteria	Subjects aged over 1 year and less than 19 years will be eligible for inclusion if they have relapsing SSNS, defined as **having experienced 2 or more relapses in the preceding 12 months**. This will include the following groups:<br>• Subjects on no long-term immunosuppressive therapy;<br>• Subjects receiving long term maintenance prednisolone therapy at a dose of up to and including $15mg/m^2$ on alternate days. Note that this

	is the maximum dose at the time of recruitment – if children subsequently receive a higher dose e.g. after relapse, they can remain in the study;<br>• Subjects receiving long term maintenance prednisolone therapy at a dose of up to and including 15mg/m^2 on alternate days in conjunction with other immunosuppressive therapies, including levamisole, ciclosporin, tacrolimus, MMF, mycophenolate sodium and azathioprine;<br>• Subjects receiving long-term immunosuppressive therapies, including levamisole, ciclosporin, tacrolimus, MMF, mycophenolate sodium and azathioprine without long term maintenance prednisolone therapy.<br>• Subjects who have previously received a course of oral or intravenous cyclophosphamide:<br>o Must have experienced two relapses in the 12 months prior to randomisation (in keeping with all other subjects)<br>o Must have experienced at least one of these relapses following completion of cyclophosphamide therapy<br>o Must be at least 3 months post completion of oral or intravenous cyclophosphamide therapy<br>• Subjects who have previously received a single dose or course of intravenous rituximab:<br>o Must have experienced two relapses in the 12 months prior to randomisation (in keeping with all other subjects)<br>o Must have experienced at least one of these relapses following completion of rituximab therapy<br>o Must be at least 3 months post completion of intravenous rituximab therapy<br>• Parents and (where age appropriate) subject understand the definition of URTI and the need to commence study drug once this definition has been met.<br>• Written informed consent obtained from the subject's parents/guardians and written assent obtained from subject (where age appropriate). Subjects aged 16 years and above will provide their own written informed consent.
Exclusion Criteria	• Subjects with steroid resistant nephrotic syndrome.<br>• Subjects receiving, or within 3 months of completing a course of oral or intravenous cyclophosphamide.<br>• Subjects receiving, or within 3 months of receiving a course of rituximab.<br>• Subjects on daily prednisolone therapy at time of recruitment.

	• Subjects on a prednisolone dose of greater than $15mg/m^2$ on alternate days at time of recruitment.<br>• Subjects with a documented history of significant non-adherence with medical therapy.<br>• Subjects who will be transferred from paediatric to adult services during the 12 month study period.<br>• Subjects unable to take prednisolone tablets, even in crushed form.<br>• Known allergy to prednisolone.
Treatment Description/Arms	Active treatment arm: Daily prednisolone for a total of 6 days commenced once criteria for URTI have been met.<br>Control arm: Daily placebo for a total of 6 days commenced once criteria for URTI have been met.<br>An URTI will be defined as the presence of at least 2 of the following *for at least 24 hours*:<br>• sore throat<br>• ear pain/discharge<br>• runny nose<br>• cough (dry/barking)<br>• hoarse voice<br>• fever >37°C (measured using tympanometric electronic thermometer)<br>Subjects who are receiving a long term maintenance prednisolone **dose of up to and including $15mg/m^2$ on alternate days** (including those not on maintenance prednisolone therapy) at the time of development of URTI will receive the following;<br>Active treatment arm - prednisolone $15mg/m^2$ daily for a total of six days, through the use of additional prednisolone study drug tablets.<br>Control arm – No change to therapy through the use of placebo study drug tablets for a total of six days.<br>For example, if a subject is receiving a long term maintenance prednisolone dose of $10mg/m^2$ on alternate days, for those subjects randomised to the active treatment arm, the dose will be increased to $15mg/m^2$ daily for a total of six days. Those in the control arm will receive prednisolone $10mg/m^2$ on alternate days, with placebo tablets being administered to increase the total number of tablets so that it is identical to that being administered in the active treatment arm.<br>Subjects who are receiving a prednisolone **dose of greater than $15mg/m^2$ on alternate days** at the time of development of URTI will receive the following;<br>Active treatment arm - prednisolone at alternate daily dose given daily for a total of six days, through the use of additional prednisolone study drug tablets<br>Control arm – No change to therapy through the use of placebo study drug tablets for a total of six days.

	For example, if a subject is receiving a prednisolone dose of 30mg/m^2 on alternate days, for those subjects randomised to the active treatment arm, the dose will be increased to 30mg/m^2 daily for a total of six days. Those in the control arm will receive prednisolone 30mg/m^2 on alternate days with a matching number of placebo tablets being administered on the alternate days (3 days) for a total of 6 days.<br>Once the six day course of study drug is complete, the subject will revert to their previous long term maintenance prednisolone dose (or no prednisolone if previously not receiving this).<br>This will be repeated each and every time the subject develops an URTI meeting the designated criteria over the 12 month study period.
Primary Analysis	Incidence of first URTI-related relapse during the 12 month study period.
Secondary Analyses	Comparison between active treatment and control arms relating to;<br>i] URTI-related relapse rate.<br>ii] Overall relapse rate (i.e. URTI related relapses plus non-URTI related relapses).<br>iii] Cumulative dose of prednisolone received.<br>iv] Proportion of subjects who develop, and severity of, steroid-related adverse events including behavioural problems.<br>v] Proportion of subjects who undergo both escalation and reduction of immunosuppressive therapy.<br>vi] Economic evaluation of cost per relapse and cost per QALY gained.
Exploratory Objectives	A single 10ml sample of blood will be collected for DNA and RNA extraction. This will be divided into two 5ml portions and used to<br>i] Perform a genome wide association study to look for possible genetic loci associated with steroid sensitive nephrotic syndrome (in collaboration with Prof Detlef Bockenhauer and Professor Robert Kleta, Great Ormond Street Hospital and Institute of Child Health, University College, London)<br>ii] Perform whole exome screening, initially looking for the 22 genetic mutations known to date to be associated with nephrotic syndrome, then moving to the 4000 genes expressed in the podocyte (key cell within the kidney filter) and finally to the entire exome. RNA will be used to perform functional studies to ascertain whether any detected mutation is pathogenic (in collaboration with Dr Ania Koziell and Professor Moin Saleem, Kings College London and University of Bristol)
Safety Monitoring	Adverse events will be assessed at each study visit.

	Important expected adverse events will be actively surveyed (i.e. must be assessed to complete the case report form). Serious adverse events (SAEs) and suspected unexpected serious adverse reactions (SUSARs) occurring over the 12 month study period and up to 3 months post treatment will be reported within 24 hours to the Birmingham Clinical Trials Unit (BCTU).<br>An independent Data Monitoring and Ethics Committee (DMEC) will review adverse event data annually or more frequently if requested by the DMEC.

Study schema

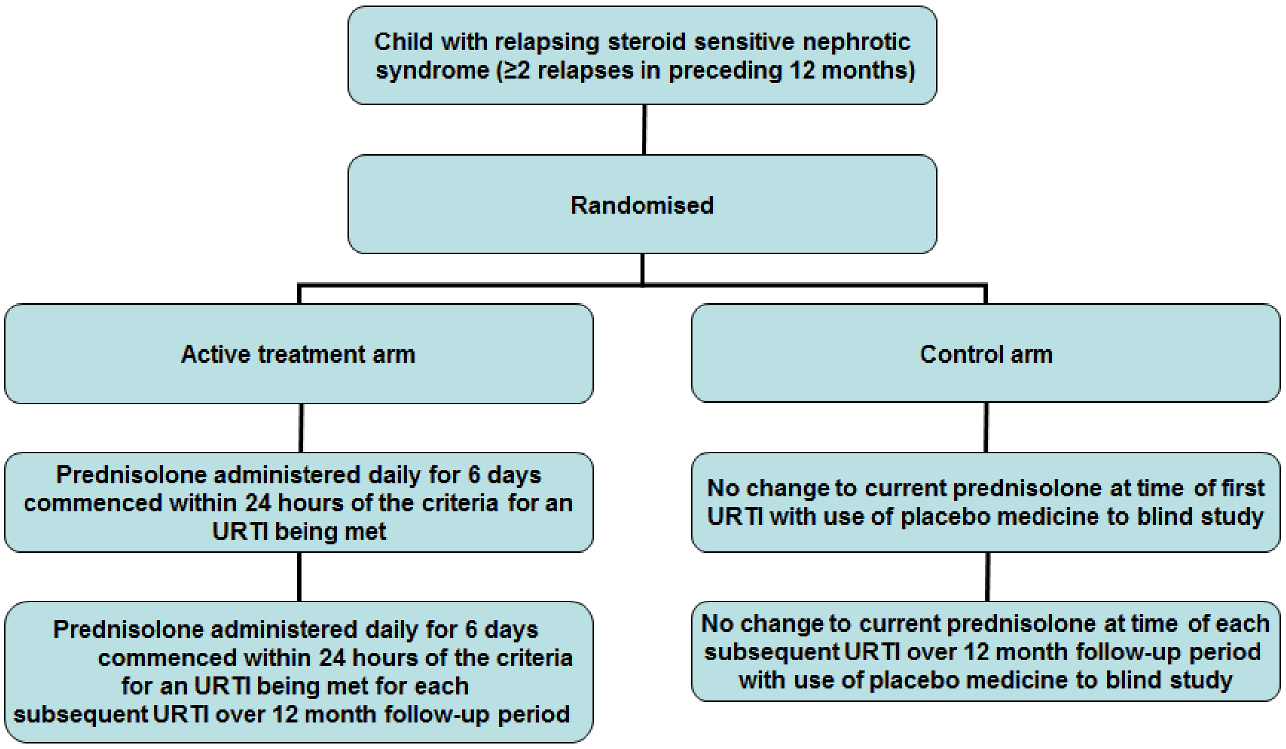

Table of Abbreviations

A+E	Accident and Emergency
ACBC	Achenbach Child Behaviour Checklist
AE	Adverse event
AR	Adverse reaction
BCH	Birmingham Children's Hospital
BCTU	Birmingham Clinical Trials Unit (also known as the Study Office)
BNF	British National Formulary
BSA	Body surface area
CHU-9D	Child Health Utility 9D
CI	Confidence interval
CRF	Case Report Form
CRN	Clinical Research Network
CTA	Clinical Trial Authorisation
DMEC	Data Monitoring and Ethics Committee
DNA	Deoxyribonucleic acid
DSUR	Development Safety Update Report
GCP	Good Clinical Practice
GP	General Practitioner
HTA	Health Technology Assessment
ICH-GCP	International Conference on Harmonisation Guidelines for Good Clinical Practice
IMP	Investigational Medicinal Product
ISF	Investigator Site File
ISKDC	International Study of Kidney Disease in Children
MCD	Minimal change disease
MHRA	Medicines and Healthcare products Regulatory Agency
MMF	Mycophenolate mofetil
NeST	Nephrotic Syndrome Trust
NIHR	National Institute for Health Research
NS	Nephrotic syndrome
Parent	Parent or guardian of the study participant
PSSRU	National Schedule for Reference Costs and the Unit Costs of Health and Social Care
QALY	Quality Added Life Years
R+D	Research and Development
RCT	Randomised controlled trial
REC	Research Ethics Committee
RNA	Ribonucleic acid
SAE	Serious Adverse Event

SAR	Serious adverse reaction
SD	Standard deviation
SmPC	Summary of Product Characteristics
SSNS	Steroid sensitive nephrotic syndrome
SUSAR	Suspected unexpected serious adverse reaction
TMG	Trial Management Group
URTI	Upper respiratory tract infection

Index

1. SUMMARY 17
2. GENERAL INFORMATION 18
2.1 Study Co-sponsors 18
2.2 Ethical approval 18
2.3 EudraCT number 18
2.4 MHRA Clinical Trials Authorisation 18
2.5 Clinical Research Network 19
2.6 Chief Investigator 19
2.7 Co-Investigators 19
2.8 Name and address of trial co-ordinator and co-ordinating centre 19
2.9 Trial Steering Committee 20
2.10 Trial Management Committee 20
2.11 Data Monitoring Committee 21
2.12 Sponsor’s medical expert for the trial 21
2.13 Protocol and protocol amendments 22
2.14 Services Involved 22
3. BACKGROUND 22
3.1 Study rationale/introduction 22
3.2 Research question 25
3.3 Investigational medicinal product 25
3.4 Name of supplier 25
3.5 Summary of known and potential risks and benefits 26
3.6 Trial conduct 26
4. TRIAL DESIGN 26
4.1 Introduction 26
4.2 Inclusion criteria 26
4.3 Exclusion criteria 27
4.4 Randomisation and allocation concealment 27
4.5 Planned interventions 28
4.6 Definition of an Upper Respiratory Tract Infection 28
4.7 Commencement of study drug 29
4.8 Dosing of study drug 30
4.9 Treatment of relapse 37
4.10 Escalation of background immunosuppressive therapy 37
4.11 The research setting 37
4.12 Trial schema and study visit schedule 38
4.13 Recruitment 38
4.14 Patient and carer information leaflet 39
4.15 Informed Consent 39
4.16 Randomisation 40

4.17 Trial treatment 41
4.18 Concomitant medications 41
4.19 Vaccination 41
4.20 Follow-up assessments 42
4.21 Study visits 42
4.22 Data Collection 44
4.23 Outcome Measures 45
4.23.1 Primary study endpoint 45
4.23.2 Secondary endpoints/outcome measures 45
4.24 Monitoring of prednisolone related adverse effects 46
4.25 Emergency unblinding 46
4.26 Withdrawal 46
4.27 Blood sample collection for DNA isolation and sub studies 47
4.27.1 Blood sampling 47
4.27.2 Genome-wide association study of steroid sensitive nephrotic syndrome .. 47
4.27.3 Other genetic studies 48
4.28 Sample Size 48
4.29 Statistical Analysis 49
4.30 Health Economic analysis 50
5. SAFETY ASSESSMENT AND REPORTING 52
5.1 Adverse Events (AEs) 53
5.2 Assessing Severity and Causality of AEs and SAEs 53
5.3 Serious Adverse Events (SAEs) 53
5.4 Reporting procedure at site 54
5.4.1 Provision of follow-up information 55
5.4.2 Reporting procedure at BCTU 55
5.5 Reporting to the Competent Authority and main Research Ethics Committee ... 55
5.5.1 Suspected Unexpected Serious Adverse Reactions (SUSARs) 55
5.6 Reporting all Types of Adverse Events 56
5.6.1 Reporting Adverse Events/Reactions 56
5.6.2 Reporting Serious Adverse Events/Reactions 56
5.7 Pharmacovigilance Responsibilities 56
5.8 Notification of Serious Breaches 57
6. DIRECT ACCESS TO SOURCE DATA/DOCUMENTS: AUDITING, MONITORING AND INSPECTION 58
7. QUALITY CONTROL AND QUALITY ASSURANCE 58
7.1 Risk Assessment 58
7.2 Data and Safety Monitoring Plan 59
8. ETHICAL CONSIDERATIONS 59
8.1 Trial Conduct 60
9. DATA HANDLING AND RECORD KEEPING 60
9.1 Trial Coordination and Data Management Centre 60
9.2 Confidentiality of Personal Data 61

9.3 Long-Term Storage of Data 62
10. RESEARCH INDEMNITY 62
11. RESEARCH GOVERNANCE 62
11.1 Quality Assurance 62
11.2 Sponsor 63
11.3 Trial Organisation 63
12. FINANCE 63
13. COST IMPLICATIONS 63
14. TRIAL COMMUNICATIONS 64
15. CLINICAL QUERIES 64
16. PUBLICATION 64
17. REFERENCES 65
18. APPENDIX 1: PREDNOS 2 TRIAL SCHEMA 66
19. APPENDIX 2: PREDNISOLONE EXPECTED ADVERSE EVENTS 67
20. APPENDIX 3: PREDNOS 2 UNBLINDING OF PATIENTS 69
21. APPENDIX 4: PREDNOS 2 QUESTIONNAIRES TABLE OF COMPLETION BY AGE.. 71

1.SUMMARY

Minimal change nephrotic syndrome is the commonest glomerular disease of childhood [Trompeter et al, 1985]. The presenting episode is treated with high dose oral prednisolone to which >90% make a complete response, responders receiving the diagnostic label of steroid sensitive nephrotic syndrome (SSNS) [Trompeter et al, 1985]. The optimum duration of prednisolone therapy at presentation remains unclear and is currently being investigated in the PREDNOS study (HTA 08/53/31).

Following successful initial treatment, 70-80% of children develop disease relapses necessitating further courses of high dose prednisolone, and around 50% develop frequently relapsing disease [International Study of Kidney Disease in Children, 1982]. Long term low dose maintenance prednisolone is the most commonly prescribed therapy to reduce relapse frequency, though a number will require additional immunosuppressive agents including levamisole, cyclophosphamide, ciclosporin, tacrolimus, mycophenolate mofetil (MMF) and rituximab.

Nephrotic syndrome relapses are associated with a risk of significant complications, including sepsis, thrombosis, dyslipidaemia and malnutrition [Webb, 2008]. The treatment of relapses with high dose prednisolone is associated with major adverse effects, including hip avascular necrosis, hypertension, diabetes and behavioural problems. Further, children are kept off school during relapses, resulting in impaired education performance and parental absence from work.

It is well recognised that at least 50% of relapses are precipitated by viral URTI, possibly mediated through release of cytokines [MacDonald et al, 1986]. Furthermore, in children with frequently relapsing SSNS, the development of an URTI results in relapse in over 50% of instances. Given these strong links between viral URTI and relapse, and the morbidity and cost associated with relapse and its treatment, it is logical that attempts are made to ameliorate the URTI-driven process.

Three studies published to date suggest that in children from developing countries (Saudi Arabia, Sri Lanka and India) with relapsing SSNS receiving long-term alternate day prednisolone therapy, the use of a short course of daily prednisolone therapy at the time of intercurrent URTI associated with fever is associated with a lower rate of subsequent relapse of nephrotic syndrome than when no such change is made to their therapy (current standard care) [Mattoo et al, 2000, Abeyagunawardena et al, 2008, Gulati et al, 2011]. These studies had a number of methodological problems, and have not identified:

1. Whether this is the case in children from developed countries, where the pattern of childhood URTI is significantly different (lower incidence of fever, absence of diarrhoea etc.);
2. Whether this effect occurs in children receiving long term maintenance prednisolone therapy in conjunction with other immunosuppressive therapies e.g. levamisole, ciclosporin, tacrolimus and MMF;
3. Whether this effect occurs in children receiving levamisole, ciclosporin, tacrolimus, and MMF without long term maintenance prednisolone therapy;
4. Whether this effect occurs in children on no long-term immunosuppressive therapy;
5. Whether there is an increase in the incidence of prednisolone-related adverse effects, including behavioural abnormality associated with the use of this intervention;
6. The cost-effectiveness analysis of such an approach, using a decision-analytic modelling approach.

The PREDNOS 2 study will provide important definitive evidence to address these outstanding questions. In this study, a total of 360 children will be randomised to receive either a six day course of daily prednisolone or no change to their ongoing immunosuppressive therapy (with the use of placebo to double blind the study) each time they develop an URTI over a 12 month period. The primary end point of the study will be the development of an URTI-related relapse after an URTI suffered during the 12 month follow-up study period. Close attention will be paid to the development of adverse effects of prednisolone therapy, including cosmetic and behavioural changes. The study will also collect detailed information on quality of life and health outcomes to allow a comprehensive health economic analysis to be performed. A single 10ml blood sample will be collected from study participants for the isolation of DNA and RNA. Using genome wide association study and total exome sequencing methodologies, we will search for potential candidate genes for SSNS and also identify potential candidate genes which might influence response to steroid therapy and the development of adverse effects.

2.GENERAL INFORMATION

2.1 Study Co-sponsors

Manchester University NHS Foundation Trust
Trust Headquarters, Cobbett House
Manchester Royal Infirmary
Oxford Road
Manchester
M13 9WL

And

The University of Birmingham
Edgbaston
Birmingham
B15 2TT
United Kingdom

2.2 Ethical approval

The study has been approved by the North West GM Central Research Ethics Committee Ref: 12/NW/0766

2.3 EudraCT number

2012-003476-39

2.4 MHRA Clinical Trials Authorisation

21761/0281/001-0001

2.5 Clinical Research Network

The study has been adopted by the NIHR Clinical Research Network: Children.

2.6 Chief Investigator

Dr Martin Christian

Consultant Paediatric Nephrologist and Network Lead for EMEESY Children's Kidney Network

Nottingham Children's Hospital

Nottingham University Hospitals NHS Trust

Queen's Medical Centre

Nottingham

NG7 2UH

2.7 Co-Investigators

**University of Birmingham**	**NIHR Medicines for Children Research Network Nephrology Clinical Studies Group**
Miss Natalie Ives	Dr David Milford (Birmingham)
Dr Emma Frew	Prof Detlef Bockenhauer (London)
Mrs Elizabeth Brettell	Prof Moin Saleem (Bristol)
	Dr Angela Hall (Leicester)
	Dr Ania Koziell (London)
	Dr Heather Maxwell (Glasgow)
	Dr Shivaram Hegde (Cardiff)
	Dr Eric Finlay (Leeds)
	Dr Rodney Gilbert (Southampton)
	Ms Jenny Booth (Newcastle)
	Dr Caroline Jones (Liverpool)
	Dr Karl McKeever (Belfast)
	Mrs Wendy Cook (consumer representative)

2.8 Name and address of trial co-ordinator and co-ordinating centre

Mrs Elizabeth Brettell (Renal Trials Manager)

Birmingham Clinical Trials Unit (BCTU)

School of Health and Population Sciences

College of Medical and Dental Sciences

Public Health Building

University of Birmingham

Edgbaston

Birmingham
B15 2TT

2.9 Trial Steering Committee

Membership of the Trial Steering Committee:

Independent Trial Steering Committee

Dr Megan Thomas (Independent Expert and Chair), Dr Nigel Coad (Independent Expert), Mrs Sandra Cope (Independent Consumer Representative), Dr Andrew Duncan (Independent Expert), Dr Darren Green (Independent Expert), Dr Ly-Mee Yu (Independent Expert Statistician).

Additional non-independent members of Trial Steering Committee

Miss Natalie Ives (BCTU Statistician), Dr Martin Christian (Chief Investigator).

Additional Trial Steering Committee Attendees (non-members)

Mrs Elizabeth Brettell (BCTU Renal Trials Manager), Professor Peter Clayton (External Expert), Mrs Wendy Cook (Consumer Representative), Dr Richard Coward (Investigator), Dr Carole Cummins (External Expert), Dr Emma Frew (Health Economist), Mr Adam Khan (BCTU Renal Data Manager), Mrs Charmaine Hunt (BCTU Renal Trial Administrator), Dr Richard Trompeter (External Expert), Miss Rebecca Woolley (Statistician), Mr Samir Mehta (Statistician).

2.10 Trial Management Committee

Dr Martin Christian (Consultant Paediatric Nephrologist)
Nottingham Children's Hospital
Nottingham

Miss Natalie Ives (Senior Statistician)
Birmingham Clinical Trial Unit (BCTU)
University of Birmingham
Birmingham

Dr Emma Frew (Senior Lecturer in Health Economics)
Health Economics Unit

University of Birmingham
Birmingham

Mrs Elizabeth Brettell (Renal Trials Manager)
Birmingham Clinical Trials Unit (BCTU)
University of Birmingham
Birmingham

2.11 Data Monitoring Committee

Membership of Data Monitoring Committee:

Professor Philip Kalra (Chairman)
Consultant Nephrologist and Honorary Professor
Salford Royal Hospital NHS Trust
Stott Lane
Salford
M6 8HD

Prof Saul Faust
Reader in Paediatric Immunology and Infectious Diseases
Southampton General Hospital
Tremona Road
Southampton
Hampshire
SO16 6YD

Dr Andrea Marshall
Senior Research Fellow in Medical Statistics
Warwick Clinical Trials Unit
The University of Warwick
Gibbet Hill Road
Coventry
CV4 7AL

2.12 Sponsor's medical expert for the trial

Dr Martin Christian (Consultant Paediatric Nephrologist)
Nottingham Children's Hospital
Nottingham, NG7 2UH
Tel: 0115 924 9924 x63832
Fax: 0115 970 9419
Email: Martin.Christian@nhs.net

2.13 Protocol and protocol amendments

Any changes will be authorised by the Chief Investigator, Dr Martin Christian, and approved by the MHRA and Health Research Authority (HRA).

2.14 Services Involved

Birmingham Children's Hospital Pharmacy will dispense the study drugs centrally for this trial.

No other services are involved.

3. BACKGROUND

3.1 Study rationale / introduction

Minimal change nephrotic syndrome is the commonest glomerular disease of childhood [Trompeter et al, 1985]. The presenting episode is treated with high dose oral prednisolone to which >90% make a complete response, responders receiving the diagnostic label of steroid sensitive nephrotic syndrome (SSNS) [Trompeter et al, 1985]. The optimum duration of prednisolone therapy at presentation remains unclear and is currently being investigated in the PREDNOS study (HTA 08/53/31, Chief Investigator Prof Nicholas Webb).

Following successful initial treatment, 70-80% of children develop disease relapses necessitating further courses of high dose prednisolone, and around 50% develop frequently relapsing disease [International Study of Kidney Disease in Children, 1982]. Long term low dose maintenance prednisolone therapy is the most commonly prescribed therapy to reduce relapse frequency, though a number will require additional immunosuppressive agents including levamisole, cyclophosphamide, ciclosporin, tacrolimus and mycophenolate mofetil (MMF).

Nephrotic syndrome relapses are associated with a risk of significant complications, including sepsis, thrombosis, dyslipidaemia and malnutrition [Webb, 2008]. The treatment of relapses with high dose prednisolone is associated with major adverse effects, including hip avascular necrosis, hypertension, diabetes and behavioural problems. Further, children are kept off school during relapses, resulting in impaired education performance and parental absence from work.

It is well recognised that at least 50% of relapses are precipitated by viral URTI, possibly mediated through release of cytokines [MacDonald et al, 1986]. Furthermore, in children with frequently relapsing SSNS, the development of an URTI results in relapse in over 50% of instances. Given these strong links between viral URTI and relapse, and the morbidity and cost associated with relapse and its treatment, it is logical that attempts are made to ameliorate the URTI-driven process.

Summary of previous studies investigating the use of daily prednisolone therapy at the time of URTI

Current practice in the large majority of UK centres is for no change to be made to immunosuppressive therapy at the time of development of an URTI. Three previous studies have assessed whether the use of daily prednisolone at the time of URTI reduces the subsequent risk of relapse of nephrotic syndrome.

Mattoo *et al* studied 36 Saudi children with relapsing SSNS who were receiving a long-term maintenance dose of alternate day prednisolone ‘of about 0.5mg/kg/day’ [Mattoo et al, 2000]. Starting on the day of onset of URTI (defined by onset of cough and/or cold with or without fever) subjects were alternatively assigned in an unblinded manner to receive either 5 days of daily prednisolone or to remain on alternate day prednisolone. They were then followed for a total of 2 years. The number of disease relapses (defined as Albustix 3+ positivity on morning urinalysis for 3 days) was documented in each group. Patients who did not relapse and patients who did not achieve remission with cyclophosphamide were both excluded. In the 18 children assigned to daily prednisolone at the time of URTI, the rate of relapse was lower than in the 18 children who continued on alternate day prednisolone (relapse rate (mean ± standard deviation (SD)): 2.2 ± 0.87 vs. 5.5 ± 1.33, p=0.04). The intervention arm received a median of 7 courses of daily prednisolone over the 2 year follow-up period. No difference was noted in the frequency of hospitalisations or the length of treatment for relapses between the two treatment arms.

Abeyagunawardena *et al* recruited 48 Sri Lankan children into a randomised double-blind cross-over study [Abeyagunawardena et al, 2008]. All were receiving long-term low dose alternate day prednisolone (mean 0.36mg/kg; range: 0.1 to 0.6mg/kg). Subjects were studied over 2 consecutive URTIs (defined as cough, runny nose, sore throat, lethargy, body aches, fever with no evidence of bacterial infection) and randomised to receive either daily prednisolone for 7 days or to continue on alternate day prednisolone (with placebo being administered on non treatment days to maintain the blind). Those who received daily prednisolone for the first URTI received alternate day prednisolone plus placebo for the second URTI, and vice versa. Children were reviewed on days 3 and 7 to assess for evidence of disease relapse (defined as Albustix 3+ proteinuria for 3 consecutive days). Those who developed prednisolone related adverse events; those who relapsed frequently and were prescribed steroid sparing therapy; those in whom prednisolone was discontinued due to sustained remission; and those who did not have two viral infections were all excluded from the study (8 of 48 recruited, thus 40 children completed the trial). Overall, there were 7 (18%) relapses following 40 URTIs treated with daily prednisolone and 19 (48%) relapses following URTI where alternate day prednisolone and placebo were administered (p=0.014; 2-sided Fishers exact test). No significant adverse effects were encountered.

The most recent and largest study was performed in 100 Indian children recently diagnosed with frequently relapsing SSNS ‘eligible for therapy with long-term alternate day prednisolone with or without levamisole’ [Gulati et al, 2011]. Children were recruited in stable remission, having received alternate day prednisolone 1.5mg/kg for 4 weeks, and then tapered by 0.25mg/kg every 2 weeks, until a dose of 0.5-0.75mg/kg on alternate days was reached. If a dose of prednisolone greater than 1mg/kg on alternate days was required, then levamisole was added. Children were randomised (stratified according to whether they received levamisole (n=32) or not (n=68)) to either daily prednisolone for 7 days or to remain on alternate day prednisolone in an unblinded manner at the time of development of URTI (defined as fever >38°C, rhinorrhoea or cough for >1 day and diarrhoea for >2 days). Children were reviewed every 2 months for a total of 12 months. The primary endpoint was the incidence of infection-associated relapse, with secondary

endpoints of infection frequency and type and cumulative dose of prednisolone. Patients exited the study if there were 2 or more relapses in any 6 month period. Daily prednisolone therapy at the time of URTI resulted in a reduction in the incidence of infection-associated relapse (0.7 vs. 1.4; rate difference: 0.7, 95% confidence interval (CI): 0.3 to 1.1; $p<0.01$) and the overall relapse rate (0.9 vs. 1.8; rate difference: 0.9, 95% CI: 0.4 to 1.4; $p<0.0001$). Whilst not powered to do so, a sub-analysis showed that this difference was lost in those receiving levamisole. Nineteen children in the daily prednisolone group vs. 7 in the alternate day group remained relapse-free over the entire 12 month study period ($p=0.03$). There was no difference in cumulative prednisolone dose between the two arms. More infections occurred in the daily prednisolone group (226 vs. 161; $p=0.04$), though no difference was detected in height SDS, Cushingoid features, cataract or serious infection. A total of 6 children (2 in daily steroid group) exited the study because of treatment failure necessitating treatment with cyclophosphamide or calcineurin inhibitors.

Critique of previous studies investigating the use of daily prednisolone therapy at the time of URTI and summary of findings

The first two studies were small, underpowered, and contained a number of methodological and analysis flaws [Mattoo et al, 2000; Abeyagunawardena et al, 2008]. Mattoo's patients were assigned to treatment group on an alternate basis, which could have introduced bias due to lack of allocation concealment. The use of a cross-over design in Abeyagunawardena's study may have resulted in the first treatment course influencing the second, required each patient to have two URTIs (which was not the case in 3 patients) and only studied patients over the course of two relapses. Mattoo's study made no mention of power calculations, and whilst Abeyagunawardena stated that the study was aiming to detect a 50% reduction in relapse rate, the sample size required to achieve this was not stated. Adverse event incidence reporting was also sparse. The Mattoo and Gulati studies were not placebo-controlled, and in the latter all patients recruited had early steroid dependent disease. In patients receiving alternate day levamisole in combination with alternate day prednisolone, there was no statistically significant benefit associated with the use of daily prednisolone during URTI, however the study was not powered to answer this question. Finally, all studies excluded patients from the analyses for a variety of reasons which could have introduced bias (e.g. patients who did not suffer any relapses, patients who discontinued prednisolone treatment due to sustained remission, patients who continued to suffer frequent relapses and were prescribed alternative immunosuppressive therapies).

In summary, the three studies published to date suggest that in children from developing countries with relapsing SSNS receiving long-term alternate day maintenance prednisolone therapy, the use of daily prednisolone therapy at the time of intercurrent URTI associated with fever is associated with a lower rate of subsequent relapse of nephrotic syndrome than when no such change is made to their therapy (current standard care). However, as outlined above, these studies had a number of methodological problems, and these studies have not identified:

i] Whether this is the case in children from developed countries, where the pattern of childhood URTI is significantly different (lower incidence of fever, absence of diarrhoea etc.);

ii] Whether this effect occurs in children receiving long term maintenance prednisolone therapy in conjunction with other immunosuppressive therapies e.g. levamisole, ciclosporin, tacrolimus and MMF;

iii] Whether this effect occurs in children receiving levamisole, ciclosporin, tacrolimus, and MMF without long term maintenance prednisolone therapy;

iv] Whether this effect occurs in children on no long-term immunosuppressive therapy;

v] Whether there is an increase in the incidence of prednisolone-related adverse effects, including behavioural abnormality associated with the use of this intervention;

vi] The cost-effectiveness analysis of such an approach, using a decision-analytic modelling approach.

A meeting was convened in January 2012 to discuss this study to which the members of the NIHR Medicines for Children Research Network (MCRN) Nephrology Clinical Studies Group, representing each of the 13 tertiary paediatric nephrology centres in the UK were invited to attend. A number of consumer representatives, including the Chairperson of the UK Nephrotic Syndrome Trust (NeST), and other parents of children with nephrotic syndrome were also in attendance. It was clear from this meeting that interest not only lay in the two cohorts originally included in the expression of interest, but also in those receiving other immunosuppressant therapies (with or without prednisolone) for their nephrotic syndrome. A single pragmatic trial was designed to include all patients with relapsing SSNS, in order to answer the overarching question of whether treatment with a short course of daily prednisolone at the time of upper respiratory tract infection (URTI) in children with relapsing SSNS reduces the subsequent development of nephrotic syndrome relapse. The results of the study will therefore be widely generalisable.

If this study were to show that the administration of a short course of daily prednisolone at the time of URTI effectively and safely reduces the rate of disease relapse in UK children this would alter practice both in the UK and other developed nations. There are already a small number of centres who routinely increase prednisolone doses at the time of URTI in individual patients, but the large majority do not because of a lack of sound evidence upon which to base such practice. A reduction in the nephrotic syndrome relapse rate would reduce relapse and treatment-associated morbidity, hospitalisation rates, and reduce parental time absent from work.

3.2 Research question

Does a six day course of daily prednisolone given early in the course of URTI in children with relapsing SSNS effectively and safely reduce the incidence of subsequent URTI-related relapse?

3.3 Investigational medicinal product

Prednisolone is a licensed corticosteroid immunosuppressant. Subjects will receive a supply of 100 tablets of study drug; prednisolone or matching placebo tablets which are being used to blind the study. Both are produced by the same supplier.

3.4 Name of supplier

Essential Nutrition Ltd. Bank House, Saltgrounds Road, Brough, HU15 1EG, UK

3.5 Summary of known and potential risks and benefits

Please use section 4.8 of the Wockhardt UK Ltd dated 31st March 2008 SmPC for prednisolone tablets. Please see Appendix 2 for Prednisolone Expected Adverse Events.

IMPs will only be used for trial subjects.

3.6 Trial conduct

This trial will be conducted in compliance with the protocol, Good Clinical Practice (GCP) and the applicable regulatory requirements.

4.TRIAL DESIGN

4.1 Introduction

Design: PREDNOS 2 will be conducted as a phase III randomised parallel group, placebo-controlled, double-blind trial.

Population to be studied: Children with relapsing SSNS (≥2 relapses in preceding 12 months).

Active treatment arm: Prednisolone 15mg/m^2/day as a single morning dose for six days commenced within 24 hours of the criteria for an URTI being met.

Control arm: Placebo (identical number of tablets) as a single morning dose for six days commenced within 24 hours of the criteria for an URTI being met.

4.2 Inclusion criteria

Subjects aged over 1 year and less than 19 years will be eligible for inclusion if they have relapsing SSNS, defined as **having experienced 2 or more relapses in the preceding 12 months**. This will include the following groups:

- Subjects on no long-term immunosuppressive therapy;
- Subjects receiving long term maintenance prednisolone therapy at a dose of up to and including 15mg/m^2 on alternate days;
- Subjects receiving long term maintenance prednisolone therapy at a dose of up to and including 15mg/m^2 on alternate days in conjunction with other immunosuppressive therapies, including levamisole, ciclosporin, tacrolimus, MMF, mycophenolate sodium and azathioprine;
- Subjects receiving long-term immunosuppressive therapies, including levamisole, ciclosporin, tacrolimus, MMF, mycophenolate sodium and azathioprine without long term maintenance prednisolone therapy.
- Subjects who have previously received a course of oral or intravenous cyclophosphamide:
    - Must have experienced two relapses in the 12 months prior to randomisation (in keeping with all other subjects)
    - Must have experienced at least one of these relapses following completion of cyclophosphamide therapy
    - Must be at least 3 months post completion of oral or intravenous cyclophosphamide therapy.
- Subjects who have previously received a single dose or course of intravenous rituximab:

    - Must have experienced two relapses in the 12 months prior to randomisation (in keeping with all other subjects)
    - Must have experienced at least one of these relapses following completion of rituximab therapy
    - Must be at least 3 months post completion of intravenous rituximab therapy.
- Parents and (where age appropriate) subject understand the definition of URTI and the need to commence study drug once this definition has been met.
- Written informed consent obtained from the subject's parents/guardians and written assent obtained from subject (where age appropriate). Subjects aged 16 years and above will provide their own written informed consent.

It is expected that there will be relatively even representation of subjects across the four background therapy groups (no background immunosuppressive therapy, long term maintenance prednisolone therapy, long term maintenance prednisolone therapy plus other immunosuppressive therapies, other immunosuppressive therapies alone), but this will be monitored during the trial. There is no evidence to suggest that the incidence of URTI is different with any one treatment regimen compared with the others, or that the likelihood of URTI precipitating a relapse of nephrotic syndrome differs according to this background immunosuppressant therapy.

4.3 Exclusion criteria

- Subjects with steroid resistant nephrotic syndrome
- Subjects receiving, or within 3 months of completing a course of oral or intravenous cyclophosphamide
- Subjects receiving, or within 3 months of receiving a course of rituximab
- Subjects on daily prednisolone therapy at time of recruitment
- Subjects on a prednisolone dose of greater than 15mg/m^2 on alternate days at the time of recruitment*
- Subjects with a documented history of significant non-adherence with medical therapy
- Subjects who will be transferred from paediatric to adult services during the 12 month study period
- Subjects unable to take prednisolone tablets, even in crushed form
- Known allergy to prednisolone

*This applies to dose of prednisolone at the time of randomisation only. If prednisolone dose increases during the 12 month study period e.g. after a relapse, subject may remain in the study.

4.4 Randomisation and allocation concealment

Following the informed consent process, which will be conducted in accordance with current GCP [Medical Research Council], and completion of the baseline assessments, subjects will be randomised at the level of the individual on a 1:1 basis to either the **active treatment** or **control** arm. Randomisation will be provided by a computer generated programme at the Birmingham Clinical Trials Unit (BCTU). The randomisation will be stratified by the treatment regimen that the subject is receiving at randomisation (no treatment; long term maintenance prednisolone therapy only; long term maintenance prednisolone therapy with other immunosuppressant therapy; other immunosuppressant

therapy only). Treatment allocation will only be revealed to the central pharmacy dispensing the study drug at Birmingham Children's Hospital.

Study drug will be manufactured, packed and labelled by Essential Nutrition. Following randomisation, subjects will be provided with a supply of 100 tablets (2 containers of 50 tablets) of study drug (prednisolone 5mg for those randomised to the **active treatment arm** or matching placebo for those randomised to the **control arm**). This will be sent directly to the family home from the central pharmacy at Birmingham Children's Hospital by Royal Mail Special Delivery on a day convenient for the family. The study drug containers and their contents will appear identical in every way, thus maintaining the double-blind. Study drug labelling will comply with the applicable regulatory requirements and clinical trial specific labels will be attached to all treatment. Birmingham Children's Hospital Pharmacy will maintain drug accountability logs for dispensed and returned study drug according to their local policy. Central pharmacy at Birmingham Children's Hospital must document when contacted by the parent/patient to confirm receipt of IMP, as below.

Every subject participating in the study will also be issued with a standard pack containing:

i. Subject diary in which the results of the morning urinalysis, treatment administration and any consultations with healthcare professionals (GP, nurse, hospital A+E department etc.), development of URTI, commencement of study drug and details of medicines prescribed or purchased over the counter are to be recorded.
ii. Instructions on contacting the central pharmacy at Birmingham Children's Hospital on receipt of IMP at the family home. This will include date of receipt and number of bottles received.
iii. Electronic tympanometric thermometer to measure temperature at the time of suspected URTI (see below).
iv. Written and (if requested) electronically downloadable information regarding URTI definition.
v. Written and (if requested) electronically downloadable information regarding dose of study drug to be commenced in the event of URTI developing.
vi. Other 'aide memoirs' regarding definition of URTI etc. displayed on fridge magnets and laminated sheets.

4.5 Planned interventions

Current practice is for no change to be made to immunosuppressive therapy at the time of development of an URTI. The intervention being assessed within PREDNOS 2 is a 6 day course of daily prednisolone therapy at the time of onset of URTI.

Those randomised to the **active treatment arm** will commence on a 6 day course of daily prednisolone each time they develop an URTI (see below for strict definition) during the 12 month follow-up period. Those randomised to the **control arm** will receive an identical number of placebo tablets, thus allowing double blinding of the study. If the subject is receiving background long-term immunosuppressive therapy (e.g. levamisole, ciclosporin), then this should continue unchanged (see section 4.8).

4.6 Definition of an Upper Respiratory Tract Infection

An URTI will be defined as the presence of at least 2 of the following *for at least 24 hours*:

- sore throat
- ear pain/discharge
- runny nose
- cough (dry/barking)
- hoarse voice
- fever >37°C (measured using tympanometric electronic thermometer)

Parents will be provided with clear written and (if requested) downloadable electronic information informing them of the study definition of an URTI as outlined above. They will also be provided with abbreviated versions of these printed onto laminated cards which can be kept in multiple locations within the family home including in fridge magnet format. An electronic tympanometric thermometer (Braun thermoscan) will be provided to the parents of all subjects to allow them to measure their child's temperature. A study diary will also be provided for parents to record the results of the daily morning urinalysis (standard care in children with relapsing SSNS), development of URTI, commencement of study drug, other ongoing treatment, acute illnesses and other issues.

4.7 Commencement of study drug

Once the subject meets the definition for URTI (two or more criteria for at least 24 hours), the parents will commence their child on study drug (prednisolone 5mg tablets for those in the **active treatment arm** or placebo for those in the **control arm** according to randomisation). The precise dosing regimen will have been calculated at the baseline study visit at the time of their recruitment and will be reviewed at each study visit, or between study visits if there is a change in the dose of long term maintenance prednisolone therapy. Information about the precise number of study drug tablets to administer will be provided for the family in written form, using a standard form (Advice Letter to Parent/Participants) which will be completed by the investigator. Please ensure that a copy of the Advice Letter to Parent/Participants is copied and filed in the patient notes and site file. This information is considered as usual clinical information and as such should also be recorded in the patient notes. At each assessment please recalculate the study tablets needed if the patient develops an URTI, and as necessary if the subject's height, weight, body surface area or background prednisolone therapy has changed. Please use the Dose Calculator on the online system and complete numbers in the Advice Letter to Parent/Participants. If a new Advice Letter to Parent/Participants is issued again please copy and file in the patient notes and site file and record the information in the patient notes.

It is anticipated that parents will have no difficulties in identifying that their child has met the URTI criteria and will be happy to commence study drug unassisted, having been provided with comprehensive advice at the time of their recruitment. However, to provide a secure back-up service in case of any doubt, parents will be instructed to contact their local study site, or in case this is not possible, a PREDNOS 2 study telephone number will be provided to the parents of all study subjects. This will be manned by the Chief Investigator or his nominated deputy during periods of annual leave etc. Parents will be able to seek advice regarding whether their child meets URTI criteria, the dose of study drug required, or any other issues relating to the study. A log of these telephone calls will be maintained, and the Chief Investigator will report their content to the local Principal Investigator by email.

To ensure patient safety, the information provided to parents will also contain information about signs of more serious infection (non-blanching rash, leg pain, cool extremities, rapid breathing, blue lips, fitting, unconsciousness or any other major concern). If any of these features are present, parents will be instructed not to start study drug and to seek urgent medical attention for their child from their GP or local Accident and Emergency Department.

Parents will be asked to contact their local study site before or within 24 hours of commencing study drug to inform the Principal Investigator or nominated deputy and to allow them the opportunity to discuss any of their child's symptoms that may be of concern to them. Please ensure, in accordance with GCP, that all contacts with parents/patients are recorded in the patient's medical notes, including telephone and email contacts. Paediatric centres regularly receive a significant number of telephone calls from the parents of children with SSNS as part of routine clinical care (e.g. for advice on the management of disease relapses, requests for information following exposure to infections etc.). Parents of children with SSNS all routinely perform regular and careful home-based monitoring of their child's condition, including daily urinalysis and monitoring for exposure to and development of infection. They are instructed to contact the paediatric centre when proteinuria recurs or whenever there are other concerns and thus the telephone call for the purpose of this study will represent a simple extension of this well established practice, but must be recorded in the medical notes.

This process should be repeated every time the child develops an URTI over the 12 month follow-up period. The only exception to this is if the child is receiving daily prednisolone therapy e.g. in the early stages of treatment for a previous relapse – in this instance study drug should not be commenced.

4.8 Dosing of study drug

The number of tablets of study drug (prednisolone 5mg for those in the **active treatment arm** and matching placebo for those in the **control arm**) to be administered once URTI develops will be calculated at the time of the baseline (randomisation) visit and will be reviewed at each subsequent visit, to allow any adjustment necessary because of changes in the child's body surface area or long term maintenance prednisolone dose.

1] Subjects not receiving long term maintenance prednisolone therapy at time of development of URTI

Subjects should receive a total number of study drug tablets equivalent to a prednisolone dose of 15mg/m^2 (maximum dose 40mg) for a total of 6 days given as 5mg tablets. Dosing should be rounded up or down to the nearest 5mg (maximum dose 40mg).

Example 1: A subject with a body surface area of 1.0 m^2.

Note: Body surface area is frequently used for drug dosing in paediatric practice and is most simply calculated using the Mosteller formula BSA=√(height*weight/3600) [Mosteller, 1987].

Prednisolone dose = 15mg daily for 6 days = 3 x 5mg tablets daily for 6 days

Subject should therefore receive 3 tablets of study drug (prednisolone for those in the **active treatment arm** or placebo for those in the **control arm**) for 6 days.

Example 2: A subject with a body surface area of 1.2m^2

Prednisolone dose = 18mg daily for 6 days, rounded up to 20mg daily for 6 days = 4 x 5mg tablets daily for 6 days

Subject should therefore receive 4 tablets of study drug (prednisolone for those in the **active treatment arm** or placebo for those in the **control arm**) for 6 days.

Example 3: A subject with a body surface area of 0.8m^2

Prednisolone dose = 12mg daily for 6 days, rounded down to 10mg daily for 6 days = 2 x 5mg tablets daily for 6 days

Subject should therefore receive 2 tablets of study drug (prednisolone for those in the **active treatment arm** or placebo for those in the **control arm**) for 6 days.

Study drug should always be given as a single dose in the morning.

Co-administration of other immunosuppressive and other medications

If the subject is receiving an additional immunosuppressive therapy e.g. ciclosporin, levamisole etc., this should continue unchanged throughout the six day course of study drug. Other drug treatment should also continue unchanged.

At the end of the 6 day course of study drug, the subject should continue as previously, i.e. taking no prednisolone. Other drug treatment should continue unchanged.

The study drug dose to be used on development of an URTI may change during the study due to alterations in the subject's long-term maintenance prednisolone dose and normal childhood growth. The precise regimen to be administered will be discussed with parents along with a written treatment plan at the time of recruitment and (in light of the above) will be re-discussed at each study visit. Where changes in long term maintenance prednisolone dose occur between study visits (e.g. as part of a planned reducing regimen), updated information will be sent to parents by mail or email. Parents will always have clear written instructions to inform them of the precise regimen of study drug to give to their child should they develop an URTI.

2] Children receiving long term maintenance prednisolone therapy at a dose of less than 15mg/m^2 on alternate days at time of development of URTI

Subjects should receive a total number of study drug tablets equivalent to a prednisolone dose of 15mg/m^2 (maximum dose 40mg) given as 5mg tablets. Dosing should be rounded up or down to the nearest 5mg (maximum dose 40mg).

Example 1: A subject with a body surface area of 1.0 m^2 receiving a long term maintenance prednisolone dose of 10mg (10mg/m^2) on alternate days

Note: Body surface area is frequently used for drug dosing in paediatric practice and is most simply calculated using the Mosteller formula BSA=√(height*weight/3600) [Mosteller, 1987].

At development of URTI, subject commences on study drug (5mg prednisolone or placebo) as follows so that the prednisolone dose received in the active treatment arm is 15mg/m^2 (equivalent to 15mg in this case) and those in the control arm receive an identical number of tablets, thus maintaining the double blind.

If day of commencement of study drug is a day when regular maintenance prednisolone is due,

Day 1 Prednisolone 10mg + 1 tablet of study drug

Day 2 3 tablets of study drug

Day 3 Prednisolone 10mg + 1 tablet of study drug

Day 4 3 tablets of study drug

Day 5 Prednisolone 10mg + 1 tablet of study drug

Day 6 3 tablets of study drug

If day of commencement of study drug is not a regular treatment day then,

Day 1 3 tablets of study drug

Day 2 Prednisolone 10mg + 1 tablet of study drug

Day 3 3 tablets of study drug

Day 4 Prednisolone 10mg + 1 tablet of study drug

Day 5 3 tablets of study drug

Day 6 Prednisolone 10mg + 1 tablet of study drug

This regimen will result in subjects randomised to the active treatment arm receiving prednisolone 15mg daily for 6 days and those randomised to the control arm undergoing no change in their therapy.

Example 2: A subject with a body surface area of 1.2m^2 receiving a long term maintenance prednisolone dose of 10mg (8.3mg/m^2) on alternate days

Note: Body surface area is frequently used for drug dosing in paediatric practice and is most simply calculated using the Mosteller formula BSA=√(height*weight/3600) [Mosteller, 1987].

At development of URTI, subject commences on study drug (5mg prednisolone or placebo) as follows so that the prednisolone dose received in the active treatment arm is 15mg/m^2 (equivalent to 20mg in this case – precise dose is 18mg, though rounded up to 20mg) and those in the control arm receive an identical number of tablets, thus maintaining the double blind.

If day of commencement of study drug is a day when regular maintenance prednisolone is due,

Day 1 Prednisolone 10mg + 2 tablets of study drug

Day 2 4 tablets of study drug

Day 3 Prednisolone 10mg + 2 tablets of study drug

Day 4 4 tablets of study drug

Day 5 Prednisolone 10mg + 2 tablets of study drug

Day 6 4 tablets of study drug

If day of commencement of study drug is not a regular treatment day then,

Day 1 4 tablets of study drug

Day 2 Prednisolone 10mg + 2 tablets of study drug

Day 3 4 tablets of study drug

Day 4 Prednisolone 10mg + 2 tablets of study drug

Day 5 4 tablets of study drug

Day 6 Prednisolone 10mg + 2 tablets of study drug

This regimen will result in subjects randomised to the active treatment arm receiving prednisolone 20mg daily for 6 days and those randomised to the control arm undergoing no change in their therapy.

Example 3: A subject with a body surface area of 0.8m^2 receiving a long term maintenance prednisolone dose of 10mg (12.5mg/m^2) on alternate days

Note: Body surface area is frequently used for drug dosing in paediatric practice and is most simply calculated using the Mosteller formula BSA=√(height*weight/3600) [Mosteller, 1987].

At development of URTI, subject commences on study drug (5mg prednisolone or placebo) as follows so that the prednisolone dose received in the active treatment arm is 15mg/m^2 (equivalent to 10mg in this case – precise dose is 12mg, though rounded down to 10mg) and those in the control arm receive an identical number of tablets, thus maintaining the double blind.

If day of commencement of study drug is a day when regular maintenance prednisolone is due,

Day 1 Prednisolone 10mg + 0 tablets of study drug

Day 2 2 tablets of study drug

Day 3 Prednisolone 10mg + 0 tablets of study drug

Day 4 2 tablets of study drug

Day 5 Prednisolone 10mg + 0 tablets of study drug

Day 6 2 tablets of study drug

If day of commencement of study drug is not a regular treatment day then,

Day 1 2 tablets of study drug

Day 2 Prednisolone 10mg + 0 tablets of study drug

Day 3 2 tablets of study drug

Day 4 Prednisolone 10mg + 0 tablets of study drug

Day 5 2 tablets of study drug

Day 6 Prednisolone 10mg + 0 tablets of study drug

This regimen will result in subjects randomised to the active treatment arm receiving prednisolone 10mg daily for 6 days and those randomised to the control arm undergoing no change in their therapy.

Study drug should always be given as a single dose in the morning.

Co-administration of other immunosuppressive and other medications

If the subject is receiving an additional immunosuppressive therapy e.g. ciclosporin, levamisole etc., this should continue unchanged throughout the six day course of study drug. Other drug treatment should also continue unchanged.

At the end of the 6 day course of study drug, the subject should continue on their previous dose of long term maintenance prednisolone therapy. Other drug treatment should continue unchanged.

The study drug dose to be used on development of an URTI may change during the study due to alterations in the subject's long-term maintenance prednisolone dose and normal childhood growth. The precise regimen to be administered will be discussed with parents along with a written treatment plan at the time of recruitment and (in light of the above) will be re-discussed at each study visit. Parents will always have clear written instructions to inform them of the precise regimen of study drug to give to their child should they develop an URTI.

3] Children receiving prednisolone therapy at a dose of greater than 15mg/m^2 on alternate days at the time of development of URTI

Note – this will represent the minority of study subjects, as one of the key exclusion criteria for randomisation is subjects receiving a prednisolone dose which is greater than 15mg/m^2 on alternate days. There will, however, be subjects who develop a disease relapse (either URTI related or non-URTI related) during the course of the 12 month study and may therefore be receiving a higher dose of prednisolone at the time of development of an URTI. This could be the first URTI, around which the primary end point of the study is based, or a subsequent URTI. The management should be the same in both instances and is as follows;

Subjects should receive a total number of study drug tablets equivalent to the alternate daily prednisolone dose being received (maximum dose 60mg) given as 5mg tablets. Dosing should be rounded up or down to the nearest 5mg (maximum dose 60mg).

Example 1: A subject with a body surface area of 1.0 m^2 receiving a prednisolone dose of 20mg (20mg/m^2) on alternate days

Note: Body surface area is frequently used for drug dosing in paediatric practice and is most simply calculated using the Mosteller formula BSA=√(height*weight/3600) [Mosteller, 1987].

At development of URTI, subject commences on study drug (5mg prednisolone or placebo) as follows so that the daily prednisolone dose received in the active treatment arm is equivalent to the current alternate daily dose – 20mg/m^2 (equivalent to 20mg in this case) and those in the control arm receive an identical number of tablets, thus maintaining the double blind.

If day of commencement of study drug is a day when regular maintenance prednisolone is due,

Day 1 Prednisolone 20mg + 0 tablets of study drug

Day 2 4 tablets of study drug

Day 3 Prednisolone 20mg + 0 tablets of study drug

Day 4 4 tablets of study drug

Day 5 Prednisolone 20mg + 0 tablets of study drug

Day 6 4 tablets of study drug

If day of commencement of study drug is not a regular treatment day then,

Day 1 4 tablets of study drug

Day 2 Prednisolone 20mg + 0 tablets of study drug

Day 3 4 tablets of study drug

Day 4 Prednisolone 20mg + 0 tablets of study drug

Day 5 4 tablets of study drug

Day 6 Prednisolone 20mg + 0 tablets of study drug

This regimen will result in subjects randomised to the active treatment arm receiving prednisolone 20mg daily for 6 days and those randomised to the control arm undergoing no change in their therapy.

Example 2: A subject with a body surface area of 1.2m^2 receiving a prednisolone dose of 25mg (20.8mg/m^2) on alternate days

Note: Body surface area is frequently used for drug dosing in paediatric practice and is most simply calculated using the Mosteller formula BSA=√(height*weight/3600) [Mosteller, 1987].

At development of URTI, subject commences on study drug (5mg prednisolone or placebo) as follows so that the daily prednisolone dose received in the active treatment arm is equivalent to the current alternate daily dose – 20.8mg/m^2 (equivalent to 25mg in this case) and those in the control arm receive an identical number of tablets, thus maintaining the double blind.

If day of commencement of study drug is a day when regular maintenance prednisolone is due,

Day 1 Prednisolone 25mg + 0 tablets of study drug

Day 2 5 tablets of study drug

Day 3 Prednisolone 25mg + 0 tablets of study drug

Day 4 5 tablets of study drug

Day 5 Prednisolone 25mg + 0 tablets of study drug

Day 6 5 tablets of study drug

If day of commencement of study drug is not a regular treatment day then,

Day 1 5 tablets of study drug

Day 2 Prednisolone 25mg + 0 tablets of study drug

Day 3 5 tablets of study drug
Day 4 Prednisolone 25mg + 0 tablets of study drug
Day 5 5 tablets of study drug
Day 6 Prednisolone 25mg + 0 tablets of study drug

This regimen will result in subjects randomised to the active treatment arm receiving prednisolone 25mg daily for 6 days and those randomised to the control arm undergoing no change in their therapy.

Study drug should always be given as a single dose in the morning.

Co-administration of other immunosuppressive and other medications

If the subject is receiving an additional immunosuppressive therapy e.g. ciclosporin, levamisole etc., this should continue unchanged throughout the six day course of study drug. Other drug treatment should also continue unchanged.

At the end of the 6 day course of study drug, the subject should continue on their previous dose of long term maintenance prednisolone therapy. Other drug treatment should continue unchanged.

The study drug dose to be used on development of an URTI may change during the study due to alterations in the subject's long-term maintenance prednisolone dose and normal childhood growth. The precise regimen to be administered will be discussed with parents along with a written treatment plan at the time of recruitment and (in light of the above) will be re-discussed at each study visit. Parents will always have clear written instructions to inform them of the precise regimen of study drug to give to their child should they develop an URTI.

4] Children receiving daily prednisolone at time of development of URTI

Where URTI occurs whilst children are receiving daily prednisolone e.g. in the early stages of treatment for a disease relapse, study drug should **not** be commenced. Subjects can continue to participate in the study and any subsequent URTI should be treated with study drug provided that they are receiving alternate day prednisolone by this point.

CLINICAL COURSE FOLLOWING STUDY DRUG TREATMENT AT TIME OF URTI; DEFINITION OF URTI-RELATED RELAPSE

Following commencement of study drug, parents will continue to monitor their child's urine in accordance with routine clinical care. **Where disease relapse occurs, the parents should contact their study centre in accordance with routine clinical care and treatment for disease relapse should be commenced.**

Relapse is defined as Albustix positive proteinuria (+++ or greater) for three consecutive days or the presence of generalised oedema plus 3+ proteinuria.

URTI-related relapse is defined as a relapse (as defined above) occurring within 14 days of the development of an URTI.

It is important that these definitions are adhered to for the purposes of the study, particularly for the first episode of URTI-related relapse, which defines the primary end point of the study.

4.9 Treatment of relapse

Relapses should be treated in accordance with the ISKDC relapse regimen. Prednisolone should be commenced at a dose of 60mg/m^2 daily (maximum dose 80mg) until the urine has been negative or trace for three consecutive days, then reduced to 40mg/m^2 (maximum dose 60mg) on alternate days for four weeks (14 doses). A subsequent tapering dose can be used at the individual physician's discretion.

Where relapse therapy is commenced, long term maintenance prednisolone therapy (e.g. 10mg on alternate days) is discontinued. Where relapse occurs whilst the child is receiving the six day course of study drug, this should also be discontinued. Once the relapse regimen has been completed, long term maintenance prednisolone therapy may be recommenced at any dose at the PI's discretion.

Background immunosuppressive therapy other than prednisolone e.g. ciclosporin, mycophenolate mofetil, levamisole etc. should be continued unchanged throughout the relapse treatment period.

4.10 Escalation of background immunosuppressive therapy

Subjects will only undergo intensification of background immunosuppressive therapy (i.e. the addition of a new immunosuppressive agent) where there are two or more relapses of their nephrotic syndrome (URTI-related or unrelated) in any six month period or where there are unacceptable adverse effects of prednisolone or other therapy. These subjects will remain under follow-up, though intensification of immunosuppressive therapy is an important study secondary endpoint. Similarly immunosuppressive therapy will only be discontinued where there is freedom from relapse for at least six months or where there are unacceptable adverse effects of therapy.

4.11 The research setting

The study will be performed in approximately 100 paediatric centres throughout the UK using the network of sites that we have established for the PREDNOS study. This will include the 13 paediatric nephrology centres (Alder Hey Children's Hospital, Belfast Royal Children's Hospital, Birmingham Children's Hospital, Bristol Royal Hospital for Sick Children, Evelina Children's Hospital London, Royal Hospital for Children Glasgow, Great North Children's Hospital Newcastle, Great Ormond Street Hospital London, Queen's Medical Centre Nottingham, Royal Manchester Children's Hospital, St James's University Hospital Leeds, Southampton General Hospital, University Hospital of Wales Cardiff) and Leicester Royal Infirmary.

4.12 Trial schema and study visit schedule

Please see Appendix 1 for trial schema

Visit	1	2	3	4	5
Month	0	3	6	9	12
Visit window		+/-2w	+/-2w	+/-2w	+/-2w
Inclusion/Exclusion criteria	x				
Informed Consent	x				
Randomisation	x				
Allocation of study number	x				
Documentation of URTI		x	x	x	x
Documentation of commencement of study drug		x	x	x	x
Documentation of recent relapse	x	x	x	x	x
Recent medical/drug history	x	x	x	x	x
Adverse event documentation		x	x	x	x
Compliance check (tablet count using counting triangle)***		x	x	x	x
Physical exam	x	x	x	x	x
Assessment of steroid toxicity	x	x	x	x	x
Height and weight	x	x	x	x	x
Blood pressure	x	x	x	x	x
Calculation of study drug dose to be administered in event of URTI and explanation / provision of documentation of this to parents – includes review of height, weight and BSA to confirm correct dose	x	x	x	x	x
If 3 or more courses of study drug have been administered since previous visit, confirm parental understanding of definition of URTI		x	x	x	x
Blood sample for DNA/RNA*	x*	x*	x*	x*	x*
Achenbach CBC**	x	x	x	x	x
Peds QL**	x	x	x	x	x
CHU-9D and EQ-5D**	x	x	x	x	x
Study drug returned to Birmingham Children's Hospital pharmacy for accountability					x

* Blood sample to be collected on one single occasion only. Sample required for all subjects.

** See Appendix 4 for version to be used at different ages

*** Tablet count details to be recorded in the medical notes and the CRF on each occasion

4.13 Recruitment

All children with relapsing SSNS will be under the care of a paediatric nephrologist and/or a general paediatrician, and will be reviewed at least once every 3 months in a hospital out-patient clinic. Potential subjects will be identified at the time of their routine clinic attendance. To facilitate recruitment, ethics approval will be sought for a letter and information on the trial to be mailed to the parents of potentially eligible children (and the child where age appropriate) 1 to 2 weeks prior to their appointment. This will allow time for the parent(s) and child to consider the information provided and discuss the study with their family and friends. At the clinic appointment, they will be approached by an appropriately trained member of the clinical team regarding entering the PREDNOS 2 study. This individual will discuss the trial with them in detail (informing them of any possible benefits or risks relating to participation) and also give them time to ask any questions that they may have regarding the trial. Informed consent will then be sought from the parent(s) and children (informed consent or assent according to age) who agree to enter the study. The study has been adopted by the CRN for Children, and the Local Research Networks will assist with subject identification and the recruitment process.

4.14 Patient and carer information leaflet

The conduct of the trial will be in accordance with the Principles of Good Clinical Practice and applicable regulatory requirements.

For subjects under 16 years of age, the parent's written informed consent for their child to participate in the trial and the subject's assent as appropriate given the child's competence must be obtained before randomisation and after a full explanation has been given of the treatment options and the manner of treatment allocation.

Subjects of 16-18 years of age will give their own consent to participation in the study.

Information sheets for parents, subjects aged 16-18yrs, older children and younger children will be used where appropriate.

The subject's GP will be notified, with the parent's and/or subject's consent.

Sites will use the current, ethically approved version of the consent/assent forms, patient/parent information sheets and GP letter.

4.15 Informed Consent

It is the responsibility of the Investigator (or designate e.g. Research Nurse if local practice allows and this responsibility has been delegated by the Principal Investigator as captured on the Site Signature and Delegation Log) to obtain written informed consent for each parent/subject prior to performing any trial related procedure. Investigators must ensure that they adequately explain the aim, trial treatment, anticipated benefits and potential hazards of taking part in the trial to the parent/ subject. The Investigator should also stress that the subject is completely free to refuse to take part or withdraw from the trial at any time. The parent/subject should be given ample time (e.g. up to one week) to read the Parent/Patient Information Sheet and to discuss their participation with others outside of the site research team. The parent/subject must be given an opportunity to ask questions which should be answered to their satisfaction. The right of the parent/subject to refuse to participate in the trial without giving a reason must be respected.

If the parent expresses an interest in their child participating in the trial they should be asked to sign and date the current ethically approved version of the Informed Consent Form. The subject will sign their own consent form if aged 16-18 years inclusive or, if below 16 years may sign an assent form if age appropriate

The Investigator or designate must then sign and date the form. A copy of the Informed Consent/Assent Form should be given to the parent/subject, a copy should be filed in the hospital notes, and the original placed in the Investigator Site File (ISF). Once the subject is entered into the trial the subject's trial number should be entered on the Informed Consent/Assent Form maintained in the ISF. In addition, a copy of the signed Informed Consent/Assent Form must be sent by fax to the Birmingham Children's Hospital Pharmacy Department. Details of the informed consent discussions should be recorded in the subject's medical notes, this should include; the date of the initial discussion, information regarding the initial discussion and the outcome, the date consent was given,

the name of the trial, the version number of the Parent/Patient Information Sheet, the version number of the Informed Consent Form, and that the person signing the consent form on behalf of the child has been determined to have the parental responsibility to do so. Throughout the trial the parent/subject should have the opportunity to ask questions about the trial and any new information that may be relevant to the subject's continued participation should be shared with them in a timely manner.

Electronic copies of the Parent/Patient Information Sheet and Informed Consent/Assent Forms are available from the Study Office and should be printed or photocopied onto the headed paper of the local institution.

Details of all subjects approached about the trial should be recorded on the Subject Screening/Enrolment Log and with the parent's/subject's prior consent their General Practitioner (GP) should also be informed that they are taking part in the trial. A GP Letter is provided electronically for this purpose.

4.16 Randomisation

Subjects will be randomised online via a secure 24 hour internet based randomisation service, or by a telephone call to the BCTU once informed consent has been obtained. Randomisation will take place at the time of the initial study visit, prior to the development of the first URTI to ensure that study drug has reached the parents/subject by the time the first URTI develops. The Investigator must complete the Randomisation Notepad to prepare for, and be able to, randomise. The Randomisation Notepad must be signed by the investigator to indicate that all of the eligibility criteria have been checked. It should also be noted in the medical records that the investigator has checked all of the eligibility criteria and that the patient meets all of the inclusion criteria and none of the exclusion criteria. The signed Randomisation Notepad should be kept in the investigator site file and a copy sent to the Trials Office. Only delegated staff at the Birmingham Children's Hospital Pharmacy will be able to view the treatment allocation in order to dispatch the pots of study drug. This will be done via a secure login link to the randomisation programme once a subject has been randomised. This method of randomisation will ensure that investigators and the study office are blinded to the subject's treatment allocation to active treatment or placebo. Subjects will be randomised between the two treatment groups (active treatment or placebo) in a one-to-one ratio via the BCTU online randomisation service. This secure internet-based central randomisation service is available 24 hours a day and will ensure concealment of treatment allocation. The randomisation will be carried out by means of a minimisation algorithm, to ensure balance between the arms with regard to important covariates. The minimisation variables will be background therapy (no background immunosuppressive therapy, long term maintenance prednisolone therapy, long term maintenance prednisolone therapy plus other immunosuppressive therapies, other immunosuppressive therapies alone).

Informed consent must be obtained by the local investigator or other qualified local research staff, as detailed in Section 4.15, before randomisation is performed. The investigator should then log into the online randomisation service at https://www.trials.bham.ac.uk/PREDNOS2, which will ask the investigator to confirm the eligibility criteria. Eligible subjects will then be randomised to either active treatment or placebo. The subject will also be assigned a unique trial identification number to be used on all trial related material for the subject. Confirmation of the randomisation and trial identification number will be forwarded to the trial coordinator, the local investigator and the Birmingham Children's Hospital NHS Trust Pharmacy via e-mail immediately upon randomisation.

In the event that access to the internet is unavailable, the investigator may call the BCTU (0800 953 0274) during normal office hours (9am to 5pm GMT) who will access the randomisation program, randomise the subject and inform the investigator of the trial number and ensure that the randomisation information reaches the appropriate study personnel. The randomisation list will be produced using a block design with the program Sample Size 2.0.

Once the subject has been randomised and a PREDNOS 2 trial number has been obtained, the investigator must fax/email and send a signed copy of the Clinical Trial Prescription Form and the Consent/Assent Form(s) to the Pharmacy at Birmingham Children's Hospital, Fax: 0121 333 9776, Email: pharmacy.clinicaltrialsteam@nhs.net, to order the PREDNOS 2 study drug.

4.17 Trial treatment

Prednisolone and placebo tablets will be manufactured by Essential Nutrition Ltd.

Subjects will be randomised to the active treatment or control arm. Subjects randomised to the active treatment arm will receive a supply of 100 prednisolone tablets for use at the time of development of URTI consistent with study intervention criteria. Subjects randomised to the control arm will receive an identically labelled supply of matching placebo tablets. For subjects unable to swallow the tablets whole, the prednisolone and placebo tablets can be crushed and a tablet crusher will be supplied with the study drug at the prescriber's request.

The pharmacy at Birmingham Women's and Children's NHS Trust will dispense and label the drugs and be responsible for drug accountability in line with their local policy. Study drug (2 containers of 50 tablets of prednisolone or placebo) will be contained within standard containers – these will be sent to the study subject's home or an alternative address via Royal Mail Special Delivery following subject randomisation. Subjects will receive storage information with their study drug. If requested, study drug can be sent to the local investigator who is then responsible for ensuring the subject receives the study drug.

Compliance with study drug will be assessed at each study visit; this will be done by a manual pill count by an appropriate staff member at the local site, using a standard counting triangle.

4.18 Concomitant medications

All other medications are allowed as required through the trial. These should be recorded in the subject Case Report Forms (CRFs).

4.19 Vaccination

Where the subject is due for routine vaccination, or specialised vaccinations, there are some contraindications which need to be taken into account as stated in the Department of Health's Immunisation Against Infectious Disease 1996, otherwise known as "The Green Book".

Live vaccines can, in some situations, cause severe or fatal infections in immunosuppressed individuals due to extensive replication of the vaccine strain. For this reason, severely immunosuppressed individuals should not be given live vaccines, and vaccination in immunosuppressed individuals should only be conducted in consultation with an appropriate specialist. Inactivated vaccines cannot replicate and so may be administered to immunosuppressed individuals, although they may elicit a lower response than in immunocompetent individuals.

Subjects who receive prednisolone, orally or rectally, at a daily dose (or its equivalent) of 2mg/kg/day for at least one week, or 1 mg/kg/day for one month are classified as a special risk group for being given live vaccines. Administration of live vaccines should be postponed for at least three months after immunosuppressive treatment has stopped, or three months after levels have been reached that are not associated with immunosuppression. Live vaccines include MMR and the BCG vaccine for tuberculosis. Also for parents who may wish to travel with their children other live vaccines include Yellow Fever and oral typhoid.

Where these issues arise with a subject within the trial please seek the advice of an immunologist or specialist within the field.

4.20 Follow-up assessments

All subjects will be followed-up for 12 months from randomisation, with subjects reviewed prior to randomisation and then once every three months for trial follow-up assessments (i.e. at 3, 6, 9 and 12 months post randomisation). This is entirely in keeping with the normal frequency of follow-up of this population in routine clinical practice. Follow-up assessments may be made by any appropriately qualified health professional included on the site delegation log. This may include study nurses or clinical nurse specialists as appropriate.

All parents and/or subjects will have previously been trained to perform early morning urine protein estimation as a part of routine clinical practice. They will be provided with a subject-held record book to enter the results of urine protein testing and the medication administered on a daily basis. This will be maintained for the 12 month duration of the study; monitoring of daily urinalysis forms part of routine care for this patient population. Parents will also use this diary to record any intercurrent illness and or consultations with healthcare professionals (GP, nurse, hospital A+E departments etc.), development of URTI and commencement of study medicines, along with details of medicines prescribed or purchased over the counter. Information from diaries reported by parents and subjects to consultants should be recorded in the medical records and on the study CRF. Please copy any patient diaries that are brought into clinic by the parent/participant and keep a copy in the patient notes and the site file. If the patient diaries are not returned to clinic, please record this in the patient notes. If the patient diaries are returned to clinic but are blank, please also record this in the patient notes.

4.21 Study visits

At the baseline visit:

- Confirmation of inclusion and exclusion criteria.
- Informed consent/assent.
- Randomisation and allocation of study number.

- Information will be captured on dates of last two relapses.
- Medical and drug history since diagnosis of nephrotic syndrome.
- A physical examination will be performed, including measurement of height, weight and blood pressure.
- Calculation of dose of study drug to be administered when URTI occurs and provision of written information for parents regarding this.
- Scoring of prednisolone adverse effects will be performed by use of a standardised proforma.
- In order to evaluate changes in subject behaviour associated with the different prednisolone regimens, the Achenbach Child Behaviour Checklist (ACBC) will be used. The ACBC is a standardised measure made up of 120 items measuring internalising (withdrawn, somatic complaints, anxiety/depression, thought problems) and externalising (social problems, attention problems, delinquent and aggressive) behaviour problems. A total Behavioural Problem score is calculated from these problem scales and forms the basis of comparison with age and gender-matched normative data.
- Information relating to quality of life will also be collected using the CHU-9D and EQ-5D (dependent upon subject age) and PedsQL questionnaires.

At subsequent study visits (months 3, 6, 9 and 12):

- Details will be collected about any URTI that has developed and whether study drug has been commenced.
- Information will be captured regarding recent history of relapse and any treatment received for this.
- Recent other medical/drug history will be recorded.
- Adverse events will be documented.
- A study drug compliance check will be performed (tablet count using counting triangle).
- A physical examination will be performed, including measurement of height, weight and blood pressure.
- Scoring of prednisolone adverse effects will be performed by use of a standardised proforma.
- Recalculation of dose of study drug to be administered when URTI occurs and provision of written information for parents regarding this.
- If 3 or more courses of study drug have been administered since previous visit, confirmation of parental understanding of definition of URTI.
- Information will be collected about consultations with health care professionals in primary care (including the home) and also in hospital (both outpatient and inpatient) and any treatment administered.
- A record will be made of all medicines taken, including those prescribed and those purchased over the counter.
- Repeat administration of the Achenbach, CHU-9D and EQ-5D (dependent upon subject age) and PedsQL will be performed.
- A blood sample for DNA and RNA analysis will be collected at one single visit.
- At the final (12 month) study visit, any remaining study drug will be returned to the Birmingham Children’s Hospital Pharmacy for accountability.

4.22 Data Collection

The CRF will comprise of the following forms:

Form	Summary of data recorded	Schedule for submission
Randomisation Notepad	Subject details, confirmation of eligibility and clinical details.	Details collected via online randomisation and copy sent to BCTU following randomisation.
Baseline and follow-up CRFs	Clinical details, primary care visits, hospital admissions, hospital A&E visits, hospital outpatient visits, study drug, adverse effects, subject withdrawal.	Details collected via online data entry system at each follow-up assessment time point (baseline and months 3, 6, 9 and 12)
Serious Adverse Event (SAE) Form	Details of any SAE occurring over the 12 month study period and up to 3 months post treatment	Faxed/emailed within 24 hours of research staff becoming aware of event.
CHU -9D	Child Quality of Life (parent rated)	Details collected via online data entry system after follow-up assessment at month 0, 3, 6, 9 and 12.
EQ-5D	Child Quality of Life (parent rated)	Details collected via online data entry system after follow-up assessment at month 0, 3, 6, 9 and 12.
PedsQL	Child Quality of Life (parent rated)	Details collected via online data entry system after follow-up assessment at month 0, 3, 6, 9 and 12.
Achenbach (ACBC)	Child behaviour (parent rated)	Sent to BCTU after follow-up assessment month 0, 3, 6, 9 and 12.

Data from all CRF documents, except for the Achenbach Child Behaviour Checklist and SAE form, should be entered online directly by site staff at http://www.bctu.bham.ac.uk/PREDNOS2. To access the online data entry system, authorised staff at sites will be provided with an individual secure login username and be requested to set-up a password. Completed copies of the Achenbach Child Behaviour Checklist and SAE form should be forwarded to the PREDNOS 2 Trial Office for data entry. For details regarding the return of completed SAE forms, please see section 5.4 of the protocol.

Data reported on each eCRF should be consistent with the source data or the discrepancies should be explained. If information is not known, this must be clearly indicated on the eCRF. All missing and ambiguous data will be queried. All sections are to be completed before returning.

Any information received from any source (parent/patient contact, patient diaries etc.) regarding development of URTIs and related symptoms, relapse dates and treatment, commencement of study medication, commencement or change of any medication (especially prednisolone and other immunosuppressive therapies), must be documented in the patient notes.

All missing and ambiguous data will be queried via Data Clarification Forms (DCFs). Responses should be made via amendments to the eCRF. The original DCF should be kept in the site file, and a copy of the completed DCF should be returned to the PREDNOS 2 Trial Office.

In all cases it remains the responsibility of the Investigator to ensure that the eCRF has been completed correctly and that the data are accurate.

Trial forms may be amended by the Study Office, as appropriate, throughout the duration of the trial. Whilst this will not constitute a protocol amendment, new versions of the form must be implemented by participating sites immediately on receipt.

4.23 Outcome Measures

4.23.1 Primary study endpoint:

First URTI-related relapse of nephrotic syndrome during 12 month follow-up period.

Relapse is defined as Albustix positive proteinuria (+++ or greater) for 3 consecutive days or the presence of generalised oedema plus 3+ proteinuria. URTI-related relapse is defined as a relapse occurring within 14 days of the development of an URTI.

4.23.2 Secondary endpoints/outcome measures:

Rate of URTI-related relapse of nephrotic syndrome (relapses per year)

Rate of relapse (URTI-related and non URTI-related) of nephrotic syndrome (relapses per year)

Cumulative dose of prednisolone (mg/kg and mg/m^2) received over the 12 month study period

Incidence of SAEs

Incidence of adverse effects of prednisolone including assessment of behaviour using the Achenbach Child Behaviour Checklist

Incidence of escalation of background immunosuppressive therapy (e.g. addition of ciclosporin, tacrolimus, cyclophosphamide etc.)

Incidence of reduction of background immunosuppressive therapy (i.e. cessation of long term maintenance prednisolone therapy)

Quality of life using the CHU-9D, EQ-5D and PedsQL

Cost per relapse of nephrotic syndrome

Cost per QALY gained

4.24 Monitoring of prednisolone related adverse effects

We will carefully monitor study subjects for prednisolone related adverse effects (see Appendix 2). As this is a Clinical Trial of an Investigational Medicine Product, we in any case need to document all adverse health events, their treatments and outcomes. Additionally to ensure that all adverse effects are recorded we will measure the following:

i. Height will be carefully monitored at each study visit (with the exception of telephone or home study visits) using a calibrated stadiometer with the subject in bare or stocking feet.
ii. Weight will be assessed at each study visit using calibrated scales.
iii. Body mass index standard deviation score will be calculated from i] and ii].
iv. Cushingoid features and hypertrichosis will be assessed by the clinician on a Likert scale and appetite will be assessed by the parent on a Likert scale.
v. The presence or absence of striae will be documented at each study visit.
vi. Behaviour will be assessed using the Achenbach Child Behaviour Checklist.
vii. Blood pressure will be assessed at each study visit to document the incidence of hypertension.
viii. Dipstick analysis of the urine will be performed at each study visit to look for glycosuria ***(>5mmol/l)***; where this is significant and persistent, plasma glucose will be measured in keeping with routine clinical practice.
ix. The development of significant bacterial, viral and fungal infections will be documented.
x. Use of varicella prophylaxis (zoster immune globulin or antiviral therapy) will be recorded.
xi. We will prospectively collect information regarding all episodes of consultation with General Practitioners or hospital medical teams, including data on treatments prescribed or purchased. This will be incorporated into the costs measured as part of the health economic analysis.

If any of the above prednisolone related adverse-effects meet the criteria of an SAE the event must be recorded on the SAE form and faxed to the BCTU on 0121 415 9135 within 24 hours of the research staff becoming aware of the event. Please try to ensure that the local Principal Investigator has assigned causality to the SAE before reporting.

4.25 Emergency unblinding

There may be medical emergencies where it is necessary for a subject’s treatment to be unblinded. Unblinding should only occur when there are valid medical or safety reasons. Subject always to clinical need, where possible, members of the research team will remain blinded.

A code-break will be available via the BCH Pharmacy (see Unblinding Procedure: Appendix 3).

4.26 Withdrawal

Patients who cease study drug (i.e. those who do not want to start study drug at time of URTI) will be followed up for the entire duration of the study provided that consent for their ongoing participation in the study is not withdrawn.

Where consent is withdrawn, parents and subjects may choose any of the following;

- The subject may wish to withdraw from the investigational treatment, but is willing to be followed-up according to the trial protocol (i.e. has agreed that follow-up data can be collected and used in the final analysis).
- The subject may not want to attend trial specific follow-up visits but is willing to be followed-up according to standard practice (i.e. has agreed that follow-up data can be collected at standard clinic visits and used in the trial final analysis).
- The subject is not willing to be followed up for trial purposes at any further visits (but has agreed that any data collected prior to the withdrawal of consent can be used in the trial final analysis).

4.27 Blood sample collection for DNA isolation and sub studies

There are multiple reasons to suggest that SSNS may be a genetic disorder. As part of the PREDNOS 2 study, we will obtain DNA and RNA samples to identify changes which may be the cause of or contribute to the disease process of SSNS. The discovery of genetic changes which are unique to SSNS would increase our understanding of the disease and may lead to improved, more specific therapies becoming available.

4.27.1 Blood sampling

As part of the consent process, permission will be obtained to collect a single 10ml blood sample for genetic sub studies at some point during the first year of follow-up, preferably at the time of routine venepuncture for clinical purposes. If for any reason the parents and or subject are unwilling to provide a blood sample, this will not preclude them taking part in the main study.

The 10ml blood sample will be collected into two 5ml aliquots (standard blood sample tubes) containing Potassium EDTA. Using pre-prepared stamped addressed packages designed to provide a safe transport system for biological substances compliant with UK guidelines, both samples will be posted to Professor Bockenhauer and Professor Kleta's laboratory at the Institute of Child Health, London. One of the 5ml samples will be retained there and the other collected by a member of Dr Koziell's laboratory team from Guy's Hospital, London, to where it will be transferred.

Samples will be labelled with the subject's PREDNOS 2 study number and their date of birth in mmm/yyyy format only – there will be no personal information about the subject identifiable to the bench researchers.

Subjects will be asked whether they also agree to submit their DNA for future studies to help understand genetic links to related medical conditions, but will be equally free to opt out at any point in time and not do so. In this case a subject will be able to request and be assured that their particular sample has been destroyed after analysis for the PREDNOS 2 lab sub study.

4.27.2 Genome-wide association study of steroid sensitive nephrotic syndrome

This sub study will be performed in the laboratories of Professor Robert Kleta and Prof Detlef Bockenhauer at the Institute of Child Health, Great Ormond Street and the Royal Free Hospital, London (both laboratories are part of University College, London). The aim of the study is to identify any genetic mutations which may be associated with childhood SSNS. The identification of a genetic mutation associated with the development of SSNS

would improve the understanding of the underlying disease mechanisms and might lead to more specific therapies in the future. Using DNA samples collected from study participants, a genome-wide association study will be performed utilizing up to date SNP chip technology and analysis. Identified risk alleles will undergo next generation sequencing to prove sequence variations within these alleles.

A separate application for funding for this work is currently being submitted.

4.27.3 Other genetic studies

This sub study investigating DNA mutations that might be important in the pathogenesis of relapsing SSNS will be conducted in collaboration with Dr Ania Koziell at Guy's Hospital, King's College London, and Professor Moin Saleem at the University of Bristol. The approach used will be whole exome screening. This represents a cost effective approach to identify the mutations of highest biomedical importance, generating information that can be used to help understand disease mechanisms and thus help improve treatment. As a consequence, exome screening has rapidly gained popularity in disease studies and has helped reveal many highly interesting causal loci not detectable by other methods. Initial data from whole genome sequencing has indicated that much of the diversity in the human genome is in rare DNA variants within the protein coding region or exome. These variants may cause or predispose to disease, with or without the need for an environmental trigger or modify response to treatment giving an explanation why different individuals respond to drugs in different ways. Eventually, the knowledge gained could help tailor drugs to particular individuals to get maximum response with minimum side-effects.

Preliminary data indicates that rare variants may also have a significant biological effect in childhood nephrotic syndrome, supporting their role in genetic susceptibility and progression of this disease, and possibly response to medication. Whole exome screening is the method of choice as it is more likely to provide information about genetic contribution than identifying common variants in regulatory regions; these are difficult to interpret in orphan diseases such as nephrotic syndrome with an incidence of less than 1 per 2000. Analysis of results will first examine a panel of 22 genes known to be associated with nephrotic syndrome, namely *WT1*, *NPHS1*, *NPHS2*, *CD2AP*, *LAMB2*, *LMX1B*, *TRCP6*, *INF2*, *ACTN4*, *PLCe1, RHGAP24*, *cubulin*, *MYO1E*, *COQ2, SCARB2, MHY9, APOL1, PDSS2, SMARCL1, PMM2, COL4A3, COL4A4* as these are cloned primarily through studies of families with autosomal recessive or dominant nephrotic disease, and less commonly pathogenic in the wider nephrotic population. If these test negative, bioinformatics analysis will examine the 4000 functional genes expressed in the podocyte. If no conclusive results are obtained, the remaining exome will undergo analysis. All analysis will be performed with appropriate bioinformatics support and subjected to rigorous statistical testing. Public databases of the human genome such as dBSNP and 1000genomes will be used as controls and standard mutation analysis programmes such as SIFT used to determine the functional significance of mutations.

RNA will also be isolated from the same 5ml blood sample. This will be used to perform functional studies to ascertain whether or not any identified mutation is pathogenic.

4.28 Sample Size

A total of 360 subjects will be recruited into the study (180 in each study arm).

The primary analysis will be based on a comparison of the number of **first** URTI-related relapses of nephrotic syndrome in the two study arms during the 12 month follow-up period. In children with frequently relapsing SSNS, the development of an URTI results in relapse in around 50% of instances [Abeyagunawardena et al, 2008]. In the Abeyagunawardena study, overall 40 URTIs treated with placebo were followed by 19 (48%) relapses, compared with 7 (18%) relapses in the prednisolone-treated group. This corresponds to an absolute difference of 30% (a 62.5% proportional reduction). In the first treatment period, there were 10 (45%) relapses in 22 placebo-treated children compared with 4 (22%) relapses in 18 prednisolone-treated children (23% absolute difference, 51% proportional reduction) [Abeyagunawardena et al, 2008]. This is a large treatment effect based on a small study of children on long term maintenance alternate day prednisolone therapy in a developing country. Therefore to detect a more conservative difference of 17.5% (i.e. 35% proportional reduction) in URTI-related relapse rate (i.e. from 50% to 32.5%), with 80% power, 2-sided test and alpha=0.05, requires 250 subjects in total (comparison of two proportions [Machin et al]). If we allow for 30% drop-out (e.g. subject withdrawal or lost to follow-up, or subject not having an URTI during the 12 month follow-up period), then this will require recruitment of 360 subjects. We therefore propose to recruit 360 subjects, 180 to each arm. However, if the treatment effect is more in line with the 50% reduction observed in the first treatment period of the Abeyagunawardena study, then this study has sufficient power (>95%) to detect this difference. To detect a 50% proportional reduction (i.e. 50% to 25%) with 90% power and alpha=0.05, requires 154 subjects, increasing to 220 with allowance for 30% drop-out.

4.29 Statistical Analysis

The Statistical Analysis Plan describes the planned analyses for PREDNOS 2 in full detail. A summary of the main analysis methods are described here.

The primary endpoint is the first URTI-related relapse of nephrotic syndrome (URTI-related relapse is defined as 3 days of Albustix 3+ proteinuria within 14 days of onset of an URTI) which occurs during the 12 month follow-up period. The proportion of subjects that experience an URTI-related relapse will be calculated for the two study treatment arms, and compared using a chi-squared test. Treatment effects will be expressed as relative risks with 95% confidence intervals.

This was chosen as the primary endpoint as it is hypothesised that giving daily prednisolone at time of URTI will reduce the subsequent development of disease relapse. If this hypothesis is correct, then those subjects randomised to placebo will experience more URTI-related relapses than those subjects randomised to active drug. This would mean that subjects in the placebo arm are more likely to undergo intensification of immunosuppressive therapy. This in turn would reduce the chances of the subjects in the placebo arm from having further relapses (both URTI-related and non URTI-related) which introduces bias. Therefore, all subjects will be followed-up for 12 months to also assess the relapse rates between the two treatment arms over a 12 month period as a secondary endpoint, but due to these potential biases, the primary endpoint will focus on the first URTI-related relapse. The analysis of the secondary endpoints for relapses per year will assess incidence (relapse) density rates for the two treatment arms and rate differences will be calculated for both URTI and non-URTI related relapse of nephrotic syndrome over the 12 month follow-up period. Appropriate regression analysis (e.g. Poisson) will be used to compare relapse rates in the two treatment groups, should there be an imbalance in any important co-variates (e.g. URTI rates across the two study arms).

The secondary endpoints include both continuous and categorical data items. The dichotomous data (e.g. incidence of escalation of background immunosuppressive therapy) will be analysed as per the primary outcome. Continuous data (e.g. prednisolone dose, quality of life scores) will be expressed as means and standard deviations, with differences in means and 95% confidence intervals. Longitudinal plots of the data over time will be constructed for visual presentation of the data. Data will be analysed using mixed effect repeated measures models with the interventions specified as independent variables. Baseline data will be included in the model as a covariate.

Analyses will be of all randomised subjects using intention to treat, except for those who do not have an URTI following randomisation. Over the course of the study up to the 12th July 2016, the number of patients who have completed the trial without experiencing at least one URTI is approximately 24% which is higher than the originally estimated <10% at the start of the trial. Exclusion of these subjects will result in no bias as these drop-outs are not related to the treatment and should by chance be equally distributed across the two treatment arms (this will be monitored throughout the trial). Sensitivity analyses will be performed on the 12 month data analyses due to the issues raised above. These analyses will be fully outlined in the Statistical Analysis Plan, and will include analyses with subjects censored at point of treatment escalation.

The only subgroup analyses planned are by the background treatment regimen that the subjects are on at randomisation (no long-term treatment; on long term maintenance prednisolone therapy; on other immunosuppressant therapy with long term maintenance prednisolone therapy; on other immunosuppressant therapy without prednisolone); an analysis comparing those subjects on prednisolone versus those not on prednisolone; and an analysis comparing those subjects on other immunosuppressant therapy versus those not on other immunosuppressant therapy. The study is not powered to detect differences in treatment effect across these subgroups, so these analyses will be considered as hypothesis generating.

4.30 Health Economic analysis

The economic evaluation will involve the development of a decision analytic simulation model as a framework for conducting cost-effectiveness analysis. The modelling framework will allow additional exploration of scenarios that are not being specifically investigated as part of the primary study such as changing medication at different time points through the duration of the study (based on an assumption that the efficacy would remain unchanged). A modelling framework is ideally suited to demonstrate and explore these issues whilst accounting for any inherent uncertainty.

The economic evaluation will take the form of a cost-effectiveness analysis based on a primary clinical outcome of cost per episode of URTI-related relapse of proteinuria. Utility-based outcomes will also be incorporated into the model allowing a secondary outcome to be cost per quality-adjusted life year (QALY) gained (based on CHU-9D and EQ-5D values). Four different groups of subjects will be considered in the model: (i) subjects on no long-term immunosuppressive therapy; (ii) subjects receiving long term maintenance prednisolone therapy; (iii) subjects receiving long term maintenance prednisolone therapy in conjunction with other immunosuppressive therapies; and (iv) subjects receiving long-term immunosuppressive therapies without long term maintenance prednisolone therapy. For each of these four groups, the model will make a direct comparison between two strategies implemented each time a subject develops an URTI: the strategy of administering a course of daily prednisolone for 6 days and a strategy of no change to

ongoing therapy (with the use of placebo tablets to maintain blinding). The base case economic evaluation will adopt the NHS perspective. NHS costs will include:

A. Treatment costs: medicines, management, adverse-effects, and treatment complications;

B. Consultation and follow-up costs: routine tests such as blood tests and urinalysis, number of outpatient visits, inpatient visits, and GP visits;

C. Longer term treatment costs (care for long-term adverse effects).

Resource use data such as visits to the subject's GP, hospital (as an outpatient or inpatient) and medicines supplied will be collected using subject-held record books provided for them. This information will be collected at the time of study visits. Due to the economic burden associated with relapse of URTI-related proteinuria that is placed on patients and their families, we will also conduct an economic evaluation that includes private costs such as over the counter medicines and parents' time off work. Resource use data related to these costs will also be collected using patient-held record books. Unit cost data for both NHS and private costs will be derived from nationally representative sources such as the British National Formulary (BNF), the National Schedule for Reference Costs and the Unit Costs of Health and Social Care (PSSRU).

To test the robustness of the conclusions to assumptions made in the modelling, and to sampling variation in the data used in the construction of the model, full deterministic and probabilistic sensitivity analysis will be carried out and results will be reported in terms of incremental cost effectiveness ratios and cost effectiveness acceptability curves. Costs and benefits will be discounted at the standard rate (3.5%).

Figure 1: Indicative decision analytical model

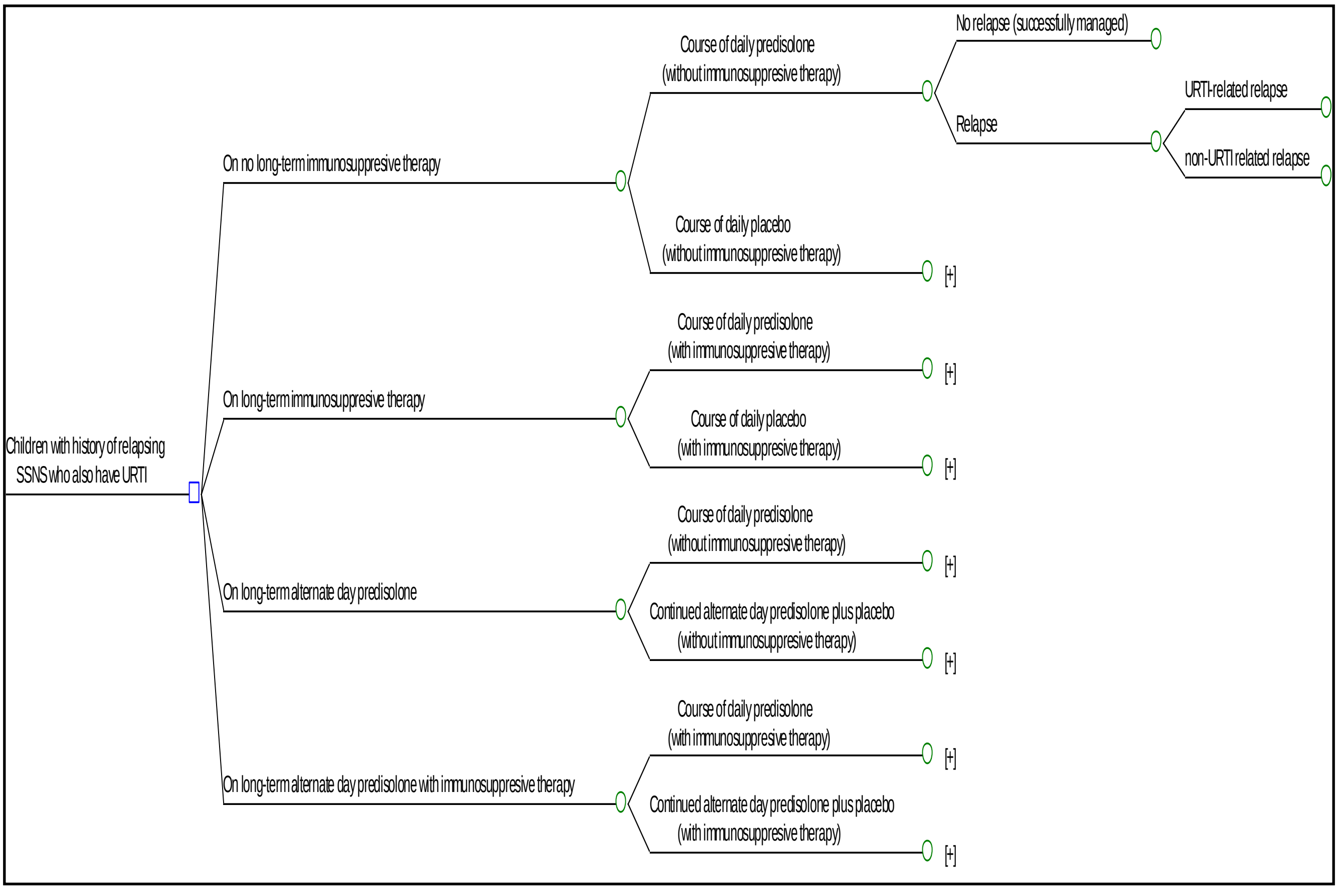

[+] means ‘same structure but with appropriate changes in probabilities’.

Figure 1 is a hypothetical illustration of the anticipated modelling approach. The decision tree presents the outcomes that are anticipated to follow either of the two treatment arms (course of daily prednisolone vs. use of placebo tablets such that there is no change to therapy). With each treatment pathway, detailed events will be modelled such as rate of relapse, adverse-effect profile and corresponding cost and quality of life effects. The detailed layout of each treatment pathway will therefore be mapped out once the trial data is collected. At the end of each treatment pathway, cost and quality of life effects will be calculated so that overall cost-effectiveness ratios for the full model can be presented.

The decision analysis model will be constructed using TreeAge Pro software. This is a widely-used and highly user-friendly package ideally suited to the construction and analysis of decision trees and other models.

5.SAFETY ASSESSMENT AND REPORTING

Pharmacovigilance reporting will comply with the Medicines for Human Use (Clinical Trials) Regulations 2004 and Amended Regulations 2006. Annual Development Safety Update Reports (DSURs) will be submitted to the main Research Ethics Committee (REC) and Medicines and Healthcare products Regulatory Agency (MHRA).

The Investigator will assess the seriousness and causality (relatedness) of all AEs experienced by the patient (this should be documented in the source data) with reference to the Prednisolone Tablet (Wockhardt UK Ltd) Summary of Product Characteristics (SmPC), dated 31/03/2008, section 4.8. This is the Reference Safety Information (RSI) for the study.

Within the PREDNOS 2 trial the steroid prednisolone and the matching placebo are both defined as Investigational Medicinal Products (IMPs).

5.1 Adverse Events (AEs)

An AE is any untoward medical occurrence in a patient or clinical trial subject administered a pharmaceutical product that does not necessarily have a causal relationship with this treatment. An AE can therefore be any unfavourable and unintended sign (including an abnormal laboratory finding), symptom or disease temporally associated with the use of a (investigational) medicinal product, whether or not related to the medicinal product.

Expected AEs are those listed in the RSI. Information on the current expected events is also given in Appendix 2.

The following are not AEs for the purposes of this trial:

- A pre-existing condition (unless it worsens significantly during treatment)
- Diagnostic and therapeutic procedures, such as surgery (although the medical condition for which the procedure was performed must be reported if new).

The causality and severity of all AEs should be recorded in the medical notes. Only AEs requested on the CRFs should be routinely reported to the PREDNOS 2 Trial Office.

5.2 Assessing Severity and Causality of AEs and SAEs

All AEs and SAEs should be evaluated by a doctor to determine severity and causality between the IMP and/or concomitant therapy and the AE.

The term “severe” is often used to describe the intensity (severity) of a specific event. This is not the same as “serious,” which is based on patient/event outcome or action criteria.

An AR is an AE judged by the reporting investigator as having a reasonable causal relationship to a medicinal product. The expression “reasonable causal relationship” means in general that there is evidence or argument to suggest a causal relationship.

5.3 Serious Adverse Events (SAEs)

All events that meet the definition of being Serious Adverse Events (see below) will be reported for the duration of the 12 month study and for 3 months following completion of study drug.

An SAE is any AE that:

- Results in death
- Is life threatening*
- Requires in-patient hospitalisation or prolongation of existing hospitalisation**
- Results in persistent or significant disability or incapacity
- Results in a congenital anomaly or a birth defect

- Or is otherwise considered medically significant by the Investigator***

*Life-threatening in this context refers to an event in which the subject was at risk of death at the time of event; it does not refer to an event which hypothetically might have caused death if it were more serious.

** Admissions that result in an overnight stay in hospital must be reported. Day admissions do not need to be reported as an SAE.

*** Medical judgment should be exercised in deciding whether an AE is serious in other situations. Important AEs that are not immediately life threatening or do not result in death or hospitalisation but may jeopardise the subject or may require intervention to prevent one of the other outcomes listed in the definition above, should be considered serious.

Investigators will report all AEs that meet the definition of being serious immediately and within 24 hours of being made aware of the event other than the following events:

Hospitalisations for:

- Routine treatment or monitoring of the studied indication, not associated with any deterioration in condition.
- Treatment, which was elective or pre-planned, for a pre-existing condition that is unrelated to the indication under study, and has not worsened.

These are expected events and do not require expedited reporting. These events must be reported on the study specific SAE form and sent to the Study Office at the BCTU within one week of the site being made aware the date of hospitalisation.

5.4 Reporting procedure at site

All Serious Adverse Events (SAE) that require expedited reporting should be reported to the Study Office at the BCTU. A trial-specific SAE form should be faxed to the BCTU immediately and within 24 hours of the research staff becoming aware of the event. The Principal Investigator or medically qualified delegate must assess the causality and severity of the event.

The assessment of causality will have 5 categories (unrelated, unlikely to be related, possibly related, probably related and definitely related) within the SAE form for the study. These will map to the binary outcome categorisation of 'related' or 'unrelated'; Unrelated and unlikely to be related will be categorised as "unrelated", whilst possibly, probably and definitely related will be categorised as related.

To report an SAE to the BCTU office, the Investigator or their delegate(s) must complete, date and sign the study specific BCTU SAE form. The completed form should be faxed or emailed to the BCTU trials team using one of the numbers listed below as soon as possible and no later than 24 hours after first becoming aware of the event:

To report an SAE, fax the SAE Form to:

0121 415 9135 and 0121 415 9136 or prednos2@trials.bham.ac.uk

On receipt of an SAE form, the BCTU trials team will allocate each SAE a unique reference number and return this via fax or email to the site as proof of receipt. If the site has not received confirmation of receipt of the SAE from the BCTU or if the SAE has not been assigned a unique SAE identification number, the site should contact the BCTU trials

team within 1 working day. The site and the BCTU trials team should ensure that the SAE reference number is quoted on all correspondence and follow-up reports regarding the SAE and filed with the SAE in the Site File.

Where an SAE Form has been completed by someone other than the Investigator, the original SAE form will be required to be countersigned by the Investigator to confirm agreement with the causality and severity assessments.

5.4.1 Provision of follow-up information

Following reporting of an SAE for a participant, the participants should be followed up until resolution or stabilisation of the event. Follow-up information should ideally be provided on a new SAE Form using the SAE reference number provided by the BCTU trials team. Once the SAE has been resolved, all follow-up information has been received and the paperwork is complete, the SAE form that was completed at site must be returned to the BCTU trials office and a copy kept in the Site File.

5.4.2 Reporting procedure at BCTU

On receipt of a faxed SAE form from the site, the BCTU trials team will allocate each SAE form with a unique reference number and enter this onto the SAE form in the section for office use only. The SAE form (containing the completed unique reference number) will be forwarded to the site as proof of receipt within 1 working day. The SAE reference number will be quoted on all correspondence and follow-up reports regarding the SAE and filed with the actual SAE in the TMF.

On receipt of an SAE Form the CI or delegate will independently determine the seriousness and causality of the SAE. An SAE judged by the CI or delegate to have a reasonable causal relationship with the trial medication will be regarded as a Serious Adverse Reaction (SAR). The causality assessment given by the PI will not be downgraded by the CI or delegate. If the CI or delegate disagrees with the PI's causality assessment, the opinion of both parties will be documented, and where the event requires further reporting, the opinion will be provided with the report.

The CI or delegate will also assess all SARs for expectedness. If the event meets the definition of a SAR that is unexpected (i.e. is not defined in the approved version of the RSI it will be classified as a Suspected Unexpected Serious Adverse Reaction (SUSAR).

5.5 Reporting to the Competent Authority and main Research Ethics Committee

5.5.1 Suspected Unexpected Serious Adverse Reactions (SUSARs)

BCTU will report a minimal data set of all individual events categorised as a fatal or life threatening SUSAR to the Medicines and Healthcare products Regulatory Agency (MHRA), main REC and Co-Sponsors within 7 days. Detailed follow-up information will be provided within an additional 8 days.

All other events categorised as non-life threatening SUSARs will be reported within 15 days.

5.6 Reporting all Types of Adverse Events

5.6.1 Reporting Adverse Events/Reactions

Adverse events relating to the patient's underlying disease and its treatment will be assessed at each study visit, and recorded on the follow-up CRFs. The BCTU will provide details of all adverse events to the Data Monitoring and Ethics Committee (DMEC) for their review on an annual basis.

5.6.2 Reporting Serious Adverse Events/Reactions

All SAEs (other than those listed in Section 5.3) must be recorded on the SAE form and faxed to the BCTU on 0121 415 9135/6 within 24 hours of the research staff becoming aware of the event. Assessment of causality **must be made by a doctor**. If a doctor is unavailable, initial reports without the causality assessment should be submitted to the BCTU by a healthcare professional within 24 hours, but must be followed up by medical assessment as soon as possible thereafter.

For each SAE, the following information will be collected:

- Full details in medical terms with a diagnosis, if possible (Information on the current expected events is given in Appendix 2)
- Action taken
- Outcome
- Causality, in the opinion of the investigator*

*Assessment of causality **must be made by a doctor**.

SAEs should be reported for the duration of the 12 month study and for 3 months following completion of study drug.

Any SAEs ongoing or still present at the end of the study must be followed up at least until the final outcome is determined, even if this means that follow-up for that patient continues after the study has ended.

5.7 Pharmacovigilance Responsibilities

Local Investigator:

- Medical judgement in assigning seriousness and causality to AEs.
- To fax SAE form to BCTU within 24 hours of becoming aware, and to provide further follow-up information as soon as available.
- To report SAEs to local committees if required, in line with local arrangements.
- To sign an Investigator's Agreement accepting these responsibilities.

Chief Investigator (or nominated individual in their absence):

- To assign causality and expected nature of SAEs where it has not been possible to obtain local assessment.
- To assess expectedness of SAEs.

- In the event of disagreement between local assessment and Chief Investigator review with regards to SUSAR status, local assessment will not be overruled, but the Chief Investigator may add comments and can upgrade if deemed appropriate prior to reporting to MHRA.

Birmingham Clinical Trials Unit:

- To report SUSARs to MHRA and REC within the required timelines.
- To notify Investigators of all SUSARs.
- To prepare annual safety reports to MHRA, REC, Trial Steering Committee (TSC), and Trial Sponsor.
- To prepare SAE reports for the DMEC at intervals to be decided by the DMEC.

Trial Steering Committee:

- To liaise with the DMEC regarding safety issues.
- The TSC may close the trial on the recommendation of the independent DMEC in the event of clear evidence of harm or benefit for one treatment regimen.

Data Monitoring and Ethics Committee:

- To review the un-blinded overall safety data to identify safety issues which may not be apparent on an individual case basis.
- To review interim analyses.
- To advise the TSC of the safety of the trial.

5.8 Notification of Serious Breaches

In accordance with Regulation 29A of the Medicines for Human Use (Clinical Trials) Regulations 2004 and its amendments, the Sponsor of the trial is responsible for notifying the MHRA in writing of any serious breach of:

- The conditions and principles of GCP in connection with that trial or;
- The protocol relating to that trial, within 7 days of becoming aware of that breach.

For the purposes of this regulation, a “serious breach” is a breach which is likely to effect to a significant degree:

- The safety or physical or mental integrity of the subjects of the trial; or
- The scientific value of the trial.

Sites are therefore requested to notify the Trials Office of a suspected trial-related serious breach of GCP and/or the trial protocol. Where the Trials Office is investigating whether or not a serious breach has occurred sites are also requested to cooperate with the Trials Office in providing sufficient information to report the breach to the MHRA where required and in undertaking any corrective and/or preventive action.

6. DIRECT ACCESS TO SOURCE DATA/DOCUMENTS: AUDITING, MONITORING AND INSPECTION

PREDNOS 2 will employ central monitoring of data. This monitoring process will be applied by the BCTU. Trials staff will be in regular contact with the site research team to check on progress and address any queries that they may have. Trials staff will check incoming CRFs for compliance with the protocol, data consistency, missing data and timing. Sites will be sent data clarification requests for inconsistencies or discrepancies.

Sites may be suspended from further recruitment in the event of serious and persistent non-compliance with the protocol and/or GCP, and/or poor recruitment. Any major problems identified during monitoring may be reported to the Trial Management Group and Trial Steering Committee and the relevant regulatory bodies. This includes reporting serious breaches of GCP and/or the trial protocol to the main REC and the MHRA.

Additional on-site monitoring visits may be triggered for example by poor CRF return, poor data quality, low SAE reporting rates, excessive number of patient withdrawals or deviations. If a monitoring visit is required the Trials Office will contact the site to arrange a date for the proposed visit and will provide the site with written confirmation. The investigators are required to permit trial-related monitoring, audits, REC review and regulatory inspection(s) providing direct access to source data/documents. Sites are also requested to notify the Trials Office of any MHRA inspections.

7. QUALITY CONTROL AND QUALITY ASSURANCE

7.1 Risk Assessment

A risk assessment will be documented by BCTU. The trial is considered to be Type A = No higher than the risk of standard medical care. All investigators and staff will follow GCP guidelines. This clinical trial will be conducted in accordance with the ethical principles that have their origin in the Declaration of Helsinki, and that are consistent with GCP and all applicable regulatory requirements.

Participants will be aged over 1 year and less than 19 years. Children are considered vulnerable trial subjects, however a trial involving children with relapsing SSNS is ethically justified as the condition is specific to children and the evidence base for treatment used in clinical practice is inadequate. The treatment being used in this trial is used in current clinical practice by some centres and children participating in the trial will face little excess clinical risk, beyond that which the patients would experience in treatment outside of a trial. Prednisolone has been in use for many decades for a wide variety of indications and is standard therapy for the treatment of SSNS. The aim of this study is to try and prevent the development of URTI related relapse, necessitating the commencement of high dose daily prednisolone therapy. There is the risk that this course of action will increase overall steroid exposure without reducing relapse rate. We will be monitoring patients every three months and will carefully document steroid related adverse events, including impact on behaviour. Those children who experience steroid toxicity during the course of the study will have their background immunosuppressive therapy enhanced in an attempt to reduce relapse frequency. There will be no additional study visits for the purposes of the study alone. The three monthly visits are in keeping with routine care in children with relapsing nephrotic syndrome.

Patients aged 16-18 years will be provided with an information sheet and consent form. For children aged under 16, there will be three different information sheets available: one for parents, one for older children and one for younger children. The clinical team will make a decision about which information sheet to use based on their assessment of the maturity of child and guidance from the parent. The parent's (and patient's if aged 16-18 years) written informed consent to participate in the trial and the child’s assent (if under 16 years) as appropriate given the child’s competence must be obtained before randomisation and after a full explanation has been given of the treatment options and the manner of treatment allocation.

Participating sites will be provided with guidelines detailing how to conduct the trial and the co-ordinating centre will check that the PI and all staff listed on the delegation log understand these at the Site Initiation prior to commencement of recruitment at the site. Only staff with suitable training and experience (as demonstrated by their current GCP certificate and signed CV) will be listed on the delegation log. Data will be monitored centrally by the trial co-ordination centre.

7.2 Data and Safety Monitoring Plan

An independent DMEC is in place for this trial (see section 2.11). The DMEC will meet annually to review all collected data and may meet more frequently if required after analysis of the available data. The DMEC will advise the Trial Management Committee and the independent TSC on the safety of continuing this clinical trial.

All SUSARs will be reported to the Trial Management Committee and the trial Co-Sponsors (University of Birmingham and Manchester University NHS Foundation Trust) by facsimile urgently. All data regarding reported adverse events will be made available to the DMEC for review.

8. ETHICAL CONSIDERATIONS

Children are considered vulnerable trial subjects, however a trial involving children with nephrotic syndrome is ethically justified as the condition is specific to children and the evidence base for treatment used in clinical practice is inadequate. It is not possible to perform the study in adult subjects and extrapolate the results to children. The active treatment arm will receive a six day course of daily prednisolone at the time of each URTI which they develop over the 12 month study period. Our hypothesis is that this will reduce the risk of subsequent relapse and reduce the total amount of prednisolone that these subjects are exposed to. There is risk that the hypothesis is incorrect and that these subjects will therefore receive unnecessary additional prednisolone treatment with its attendant adverse effects. The subjects participating in the study will already be receiving significant annual total doses of prednisolone as a result of their relapsing disease and the additional prednisolone administered through their participation in PREDNOS 2 will represent only a relatively small increase in total annual dose. We will carefully monitor adverse effects of prednisolone in this study, including the effect on child behaviour.

Informed consent will be sought from parents and subjects aged 16-18 years. Children of an appropriate age and level of education will be asked to provide signed assent.

Information sheets will be provided for parents, subjects aged 16-18 years, older child subjects and younger child subjects.

8.1 Trial Conduct

The trial will be performed in accordance with the recommendations guiding physicians in biomedical research involving human subjects, adopted by the 18th World Medical Association General Assembly, Helsinki, Finland, June 1964, amended at the 48th World Medical Association General Assembly, Somerset West, Republic of South Africa, October 1996 (website: http://www.wma.net/en/30publications/10policies/b3/index.html).

The trial will be conducted in accordance with the Research Governance Framework for Health and Social Care, the applicable UK Statutory Instruments, (which include the Medicines for Human Use Clinical Trials 2004 and subsequent amendments and the General Data Protection Regulation and Data Protection Act 2018, Human Tissue Act 2008 and the International Conference on Harmonisation Guidelines for Good Clinical Practice (ICH GCP). This trial will be carried out under a Clinical Trial Authorisation in accordance with the Medicines for Human Use Clinical Trials regulations. The protocol has been submitted to and approved by the main Research Ethics Committee (REC).

Before any patients are enrolled into the trial, local capability and capacity must be confirmed and a clinical trial site agreement between the sponsor and the site must be in place. Sites will not be permitted to enrol patients until written confirmation is received from the Trials Office.

It is the responsibility of the Principal Investigator to confirm that they have read and understood all subsequent amendments, by signing the signature page at the start of each relevant protocol. This does not affect the individual clinicians' responsibility to take immediate action if thought necessary to protect the health and interest of individual patients.

9. DATA HANDLING AND RECORD KEEPING

9.1 Trial Coordination and Data Management Centre:

Birmingham Clinical Trials Unit, College of Medical & Dental Sciences, Public Health Building, University of Birmingham, Edgbaston, Birmingham, B15 2TT
Telephone 0121 415 9131
Fax 0121 415 9135
Email: prednos2@trials.bham.ac.uk

The named clinicians at the participating sites will enter data onto the CRFs. Where this duty is delegated to other staff, this will be recorded in a delegation log.

Data from this trial will be handled by the BCTU, a full-time research facility dedicated to, and with substantial experience in, the design and conduct of randomised clinical trials. The BCTU recognises the responsibilities of a data management centre with respect to the ethical practice of research and the adequate protection of human subjects.

9.2 Confidentiality of Personal Data

The trial will collect personal data about participants, and medical records will be reviewed for all patients and routine physical examinations will be performed.

Personal data recorded on all documents will be regarded as strictly confidential and will be handled and stored in accordance with the General Data Protection Regulation and Data Protection Act 2018. With the parent/patient's consent, their date of birth will be collected at trial entry to assist with long-term follow-up. Patients will be identified using only their unique trial number and date of birth in mmm/yyyy format on the CRF and correspondence between the Trials Office and the participating site. Patient name, address, postcode, date of birth and parent's telephone numbers will be collected after randomisation on a Clinical Trial Prescription form and faxed, together with the Parent Consent Form, to Birmingham Children's Hospital Pharmacy Department to allow the pharmacy to dispense the study drug and send this directly to the parent's home address. Parents are asked to give permission for the Birmingham Children's Hospital Pharmacy Department to be sent a copy of their signed Informed Consent Form which will not be anonymised. These identifiable personal data will be stored separately from the study record.

Study drug will not be dispensed unless the Birmingham Children's Hospital have received a copy of the consent form and all of the relevant contact details to ensure that study drug is appropriately dispensed and sent to the correct address.

Investigators will keep their own study file logs which link patients with anonymised CRFs. The Investigator must maintain documents not for submission to the Trials Office (e.g. Patient Identification Logs) in strict confidence. In the case of specific issues and/or queries from the regulatory authorities, it will be necessary to have access to the complete trial records, provided that patient confidentiality is protected.

The Trials Office will maintain the confidentiality of all patient data and will not disclose information by which patients may be identified to any third party other than those directly involved in the treatment of the patient and organisations for which the patient has given explicit consent for data transfer (e.g. Birmingham Children's Hospital Pharmacy Department). Representatives of the PREDNOS 2 trial team may be required to have access to patient's notes for quality assurance purposes, but patients should be reassured that their confidentiality will be respected at all times.

The Study Drug Prescription and Parent Consent Form, which will be sent to the Birmingham Children's Hospital will, out of necessity, contain identifiable personal data. These will be stored separately from the study record. Investigators will keep their own study file logs which link patients with anonymised CRFs.

Participants will be informed that their trial data and information will be securely stored at the trial office at the BCTU, and will be asked to consent to this. The data will be stored on a secure computer database, and all personal information obtained for the study will be held securely and treated as strictly confidential. Any data processed outside of the BCTU will be anonymised.

9.3 Long-Term Storage of Data

In line with the Medicines for Human Use (Clinical Trials) Regulations, once data collection is complete on all participants, all data will be retained for up to 25 years.

Any queries or concerns about the data, conduct, or conclusions of the trial can also be resolved in this time. Limited data on the participants and records of any adverse events may be kept for longer if recommended by an independent advisory board.

Trial data will be stored within the BCTU under controlled conditions for at least 3 years after closure. Long-term offsite data archiving facilities will be considered for storage after this time. The BCTU has standard processes for both hard copy and computer database legacy archiving.

10. RESEARCH INDEMNITY

PREDNOS 2 was developed by members of the NIHR Medicines for Children Research Network Nephrology Clinical Studies Group in collaboration with the BCTU and is funded by a grant from National Institute for Health Research Health Technology Assessment programme. Manchester University NHS Foundation Trust and the University of Birmingham are the trial co-sponsors. As it is not an industry-sponsored trial, ABPI guidelines on indemnity do not apply and there are no special arrangements for compensation for any non-negligent harm suffered by patients as a result of participating in the study. The normal NHS indemnity liability arrangements for clinician initiated research will, therefore, operate – see NHS Executive Health Service Guidelines HSG (96) 48, 8th November 1996. It should be noted, however, that negligent liability remains the responsibility of the hospital, whether or not a patient is part of a clinical trial, because of the duty of care that the hospital has for their patients.

11. RESEARCH GOVERNANCE

The trial will be conducted in compliance with the principles of the Declaration of Helsinki (1996), the principles of GCP and in accordance with all applicable regulatory requirements including but not limited to the Research Governance Framework for Health and Social Care and the Medicines for Human Use (Clinical Trials) Regulations 2004, as amended in 2006 and any subsequent amendments.

The trial protocol and related documents have been submitted for Research Ethics Committee (REC) approval and Clinical Trial Authorisation (CTA) from the Medicines and Healthcare products Regulatory Agency (MHRA). Any subsequent protocol amendments will also be submitted to the REC and MHRA for approval, as appropriate. Children are considered vulnerable trial subjects, however a trial involving children with nephrotic syndrome is ethically justified as the condition is specific to children and the evidence base for treatment used in clinical practice is inadequate.

11.1 Quality Assurance

Monitoring of this trial will be to ensure compliance with GCP. A risk proportionate approach to the initiation, management and monitoring of the trial will be adopted (as per the MRC/DH/MHRA Joint Project: Risk-adapted Approaches to the Management of

Clinical Trials of Investigational Medicinal Products) and outlined in the trial-specific risk assessment.

11.2 Sponsor

Manchester University NHS Foundation Trust and the University of Birmingham will share responsibilities for co-sponsorship of the trial. The trial is being coordinated by the BCTU at the University of Birmingham. All sites will be required to sign a Clinical Study Site Agreement and Investigator's Agreement, outlining their commitment to accrual, compliance, GCP, confidentiality and publication.

11.3 Trial Organisation

Chief Investigator

Dr Martin Christian, Nottingham Children's Hospital, Nottingham

Co-Investigators

Co-Investigators – Ms Natalie Ives, Dr Emma Frew, Mrs Elizabeth Brettell

University of Birmingham

Dr David Milford, Prof Detlef Bockenhauer, Prof Moin Saleem, Dr Angela Hall, Dr Ania Koziell, Dr Heather Maxwell, Dr Shivaram Hegde, Dr Eric Finlay, Dr Rodney Gilbert, Ms Jenny Booth, Dr Caroline Jones, Dr Karl McKeever, Mrs Wendy Cook

NIHR Medicines for Children Research Network Nephrology Clinical Studies Group

Principal Investigator

Each centre should nominate one person to act as the Local Principal Investigator, who will be responsible for ensuring that all approvals are obtained for their centre and that the clinical trial site agreement is signed and in place. The responsibilities of the local Principal Investigator will be to ensure that all staff who will be involved in the trial are well informed about the trial and trained in trial procedures.

12. FINANCE

PREDNOS 2 is funded by a grant from National Institute for Health Research Health Technology Assessment programme and organised by the Department of Health funded University of Birmingham Clinical Trials Unit.

No individual per patient payment will be made to NHS Trusts, Investigators or patients.

13. COST IMPLICATIONS

PREDNOS 2 has been adopted by the Clinical Research Network (CRN) for Children. This will assist with study site and PI identification and local study set up (Trust R+D approval etc.).

Study drug will be provided free of charge and sent by Royal Mail Special Delivery to the patient's home from the Birmingham Children's Hospital Clinical Trials Pharmacy.

14. TRIAL COMMUNICATIONS

Trial investigators will be informed of trial progress in the form of electronic newsletters, annual investigators' meetings and electronic mail (emerging issues).

15. CLINICAL QUERIES

During office hours, the clinical coordinators (see inside front cover for contact details) provide an on-call service for any clinical queries about the trial.

16. PUBLICATION

A meeting will be held after the end of the study to allow discussion of the main results among the collaborators prior to publication. The success of PREDNOS 2 depends on the collaboration of a large number of staff including nephrologists and nurses. For this reason, one individual from each centre which has collaborated in the study will be listed as a contributing author. The Writing Committee will approve all publications using PREDNOS 2 data and the authorship. The Writing Committee will be composed of the Chief Investigator, Co-applicants, BCTU statistical and data analysis staff, and investigators with expertise in clinical and basic science related to childhood nephrotic syndrome.

17. REFERENCES

Abeyagunawardena AS, Trompeter RS. Increasing the dose of prednisolone during viral infection reduced the risk of relapse in nephrotic syndrome: a randomised controlled trial. Arch Dis Child 2008;93:226-8.

Gulati A, Sinha A, Sreenivas V, et al. Daily corticosteroids reduce infection-associated relapses in frequently relapsing nephrotic syndrome: a randomized controlled trial. Clin J Am Soc Nephrol 2011;6:63-9.

International Study of Kidney Disease in Children. Nephrotic syndrome in children: a randomised controlled trial comparing two prednisone regimens in steroid-responsive patients who relapse early. J Pediatr 1982; 95: 239-43.

MacDonald N, Wolfish N, Maclaine P, et al. Role of respiratory viruses in exacerbations of primary nephrotic syndrome. J Paediatr 1986;108:378-82.

Machin D, Campbell MJ, Tan SB, Tan SH (2009) Sample size tables for clinical studies. 3rd ed. Chichester: Wiley-Blackwell.

MattooTK, Mahmoud MA. Increased maintenance corticosteroids during upper respiratory infection decrease the risk of relapse in nephrotic syndrome. Nephron 2000;85:343-5.

Medical Research Council. MRC Guidelines for Good Clinical Practice in Clinical Trials. London: MRC; 1998.

Mosteller RD. Simplified calculation of body-surface area. N Engl J Med 1987; 317: 1098.

Trompeter RS, Lloyd BW, Hicks J, et al. Long-term outcome for children with minimal change nephrotic syndrome. Lancet 1985;1:368-70.

Webb NJA. Epidemiology and general management of childhood idiopathic nephrotic syndrome. In Evidence-based Nephrology Ed Molony D and Craig J. Blackwell 2008.

18. APPENDIX 1: PREDNOS 2 TRIAL SCHEMA

**Identification**	Subject identified as attending out-patient appointments at regional paediatric nephrology centre or district general hospital with a diagnosis of steroid sensitive nephrotic syndrome (SSNS)<br>“Does the subject have relapsing SSNS, with ≥2 relapses in the preceding 12 months?”	
	If yes, “Does the subject meet the inclusion/exclusion criteria?”	If no they are not eligible for the trial
	If yes, clinician sends parent and (where age appropriate) subject trial **Parent/Subject Information Leaflets** one to two weeks before next clinic assessment due	
**Information provision**	Parent/subject consider information and have time to discuss trial with family and friends. A contact number will be provided to be used if the parent/subject wants to further discuss the trial prior to their next clinic appointment.<br>Parent/subject considers entering the trial.	Parent/subject is not willing to enter the trial
	At next out-patient appointment the trial information is discussed in further detail and the parent/subject have the opportunity to ask any questions they may have about the trial.	Parent/subject is not willing to enter the trial
**Consent**	If parent/subject are willing to enter the trial arrangements made for **Informed Consent/Assent** to be obtained and for baseline assessments **(Baseline Form, CHU-9D, PedsQL, Achenbach and EQ-5D)** to be performed at that same appointment.	
**Randomisation**	Principal Investigator or nominated deputy randomises subject using the **Randomisation Notepad**.<br>Family provided with URTI diagnosis checklist and information on precise study drug dosing regimen by PI using the **Advice to Parents and Participants** sheet. Order supply of study drug using **Prescription Form** from Birmingham Children’s Hospital pharmacy. Prednisolone 5mg tablets or matching placebo will be delivered to parent’s nominated address. Family provided with clear instructions (printed with back up version sent electronically, if requested) regarding precise regimen to be administered in the event of URTI developing.	
	Subject randomised to active treatment arm.<br>At onset of each URTI, in the following 12 month period, receives 6 days prednisolone.<br>Continues other background immunosuppressive therapy if receiving this.	
	Subject randomised to control arm.<br>At onset of each URTI, in the following 12 month period, receives 6 days of matching placebo.<br>Continues other background immunosuppressive therapy if receiving this	
**Follow-up**	3, 6, 9 and 12 month assessment. Use **Follow-up Form and CHU-9D, PedsQL, Achenbach and EQ-5D** questionnaires dependent on the subject’s age. Collect blood for DNA and RNA analysis (single episode).<br>Primary end point (incidence of first URTI-related relapse).	

19. APPENDIX 2: PREDNISOLONE EXPECTED ADVERSE EVENTS

From post-marketing experience, the following side-effect incidences are commonly quoted. The occurrence of drug reactions is generally dose dependent.

Adverse reactions are listed according to the following categories:

- Very common (≥1/10)
- Common (≥1/100 to <1/10)
- Uncommon (≥1/1,000 to <1/100)
- Rare (≥1/10,000 to <1/1,000)
- Very rare (<1/10,000)

Prednisolone is associated with the following adverse drug reactions:

**Cardiac disorders**	
Uncommon:	Oedema, hypertension
**Respiratory disorders**	
Common	Epistaxis miscellaneous: Diaphoresis
**Central nervous system disorders**	
Very common	Insomnia, nervousness, behavioural change
Common	Dizziness or light-headedness, headache
Uncommon	Hallucinations, seizures
**Eye disorders**	
Common	Cataracts, glaucoma
**Gastrointestinal disorders**	
Very common	Increased appetite, indigestion
**Musculoskeletal and connective tissue disorders**	
Uncommon	Fractures, muscle-wasting, osteoporosis
**Endocrine and metabolic disorders**	
Common	Diabetes mellitus, glucose intolerance, hyperglycemias, reduced growth velocity, Cushingoid facies
Uncommon	Cushing’s syndrome, pituitary-adrenal axis suppression, pancreatitis
**Dermatologic conditions**	

Common	Hirsutism, hypo pigmentation, striae, acne
**Neuromuscular and skeletal**	
Common	Arthralgia

Prednisolone is also associated with the following SAEs – predominantly in adult patients:

- Musculoskeletal: insufficiency fractures, avascular osteonecrosis
- Cardiovascular: premature atherosclerosis, myocardial infarction
- Gastrointestinal: ulceration and bleeding
- Central nervous system: psychosis
- Endocrine: hyperosmolar non-ketotic state, Addisonian crisis
- Miscellaneous: Bacterial infections, fungal infections, viral infections, ocular herpes zoster.

20. APPENDIX 3: PREDNOS 2 UNBLINDING OF PATIENTS

PREDNOS 2 Clinical Trial

Code-break/Unblinding of Patients Procedure

Background

In the event of a medical emergency it may be important for the doctor to be aware of which treatment has been used for the patient and it may be necessary for the trial code to be broken and the treatment allocation to be revealed.

When not to unblind:

1. There should be no need for unblinding if the patient is not currently taking a 6 day course of prednisolone following an URTI, as unblinding would be extremely unlikely to influence the choice of treatment for, or prognosis of, any condition in the patient. Hence any request for unblinding during when the patient is not taking a 6 day course of prednisolone following a URTI should be referred to the Chief or Principal Investigators.

When to unblind:

1. When patient is taking study treatment, i.e. when they are taking a 6 day course of prednisolone following a URTI, unblinding should only occur when there is a clinical need to know whether the patient is receiving prednisolone, as this information is required to decide on the patient's treatment.

   e.g. i) the patient develops a condition where oral or IV prednisolone therapy is indicated, e.g. severe asthma.

   ii) the patient develops a condition where the prednisolone may need to be changed or is relevant to determining treatment, for example where a severe infection develops.

How to unblind?

1. If the PI or co-investigator decides it is necessary to unblind the patient, please request the unblinding from the Senior Clinical Trials Technician or Lead Pharmacist for Clinical Trials at Birmingham Children's Hospital Pharmacy:
   a. Tel: **0121 333 9308** (during pharmacy opening hours)
   b. Tel: **0121 333 9999** (outside pharmacy opening hours contact the on-call pharmacist through the hospital switchboard)

2. The pharmacist will record the name, post, address and contact number of the person requesting the unblinding. They will also require patient ID, name and the reason for unblinding request.

21. APPENDIX 4: PREDNOS 2 QUESTIONNAIRES TABLE OF COMPLETION BY AGE

PREDNOS 2 Questionnaires Table of Completion by Age				
**Age of Child (yrs)**	**Parent Completed Peds QL**	**Parent Completed Child Health Utility (CHD) 9D**	**Parent Completed Achenbach Childhood Behaviour Checklist**	**Parent completed EQ-5D**
**>1-1.5**	No form available	No form available	No form available	No form available
**1.5-2**	No form available	No form available	Use version 1.5-5 yrs	No form available
**2**	Use version 2-4yrs	No form available	Use version 1.5-5 yrs	No form available
**3**	Use version 2-4yrs	No form available	Use version 1.5-5 yrs	No form available
**4**	Use version 2-4yrs	No form available	Use version 1.5-5 yrs	No form available
**5**	Use version 5-7yrs	Use CHU-9D	Use version 1.5-5 yrs	No form available
**6**	Use version 5-7yrs	Use CHU-9D	Use version 6-18 yrs	No form available
**7**	Use version 5-7yrs	Use CHU-9D	Use version 6-18 yrs	No form available
**8**	Use version 8-12 yrs	Use CHU-9D	Use version 6-18 yrs	No form available
**9**	Use version 8-12 yrs	Use CHU-9D	Use version 6-18 yrs	No form available
**10**	Use version 8-12 yrs	Use CHU-9D	Use version 6-18 yrs	No form available
**11**	Use version 8-12 yrs	Use CHU-9D	Use version 6-18 yrs	No form available
**12**	Use version 8-12 yrs	Use CHU-9D	Use version 6-18 yrs	Use EQ-5D
**13**	Use version 13-18yrs	Use CHU-9D	Use version 6-18 yrs	Use EQ-5D
**14**	Use version 13-18yrs	Use CHU-9D	Use version 6-18 yrs	Use EQ-5D
**15**	Use version 13-18yrs	Use CHU-9D	Use version 6-18 yrs	Use EQ-5D
**16**	Use version 13-18yrs	Use CHU-9D	Use version 6-18 yrs	Use EQ-5D
**17**	Use version 13-18yrs	Use CHU-9D	Use version 6-18 yrs	Use EQ-5D
**18**	Use version 13-18yrs	Use CHU-9D	Use version 6-18 yrs	Use EQ-5D

Short course daily prednisolone therapy at the time of upper respiratory tract infection in children with relapsing steroid sensitive nephrotic syndrome: The PREDNOS 2 Trial

Trial Registration: ISRCTN10900733

Statistical Analysis Plan

SAP Version Number	Protocol Version Number
V2.0	v3.0

Name of Author:	Rebecca Woolley	Role:	Trial Statistician	Affiliation:	BCTU<br>University of Birmingham
Signature of Author:		Date:			
Name of Chief Investigator:	Martin Christian	Role:	Chief Investigator	Affiliation:	Nottingham Children's Hospital
Signature of Chief Investigator:		Date:			
**This Statistical Analysis Plan has been approved by:**					
Name of Approver:	Natalie Rowland	Role:	Senior Statistician	Affiliation:	BCTU<br>University of Birmingham
Signature of Approver:		Date:			

Statistical Analysis Plan (SAP) Amendments

SAP version number	SAP section number	Description of and reason for change	Timing of change with respect to interim analysis/ final analysis/ database lock	Blind Reviewer	
2.0		Review of SAP prior to final analysis, decision made to transfer to new BCTU template which is a more comprehensive document. Text from previous SAP copied over to appropriate sections in new template. New sections in template, uses template text and/or text taken directly from SAP v1.0 or the latest protocol.	Prior to final database lock	Name:	
				Signature:	
				Date:	
	4.3	Following discussion with TSC and funder, the primary outcome was changed from URTI-related relapse following the first URTI to first URTI-related relapse, so URTI-relapse could occur after any URTI during the 12 month follow-up period.			
	4.7	Sample size was revised during study, with the inflation rate applied to the sample size increased from 10-20% to 30%.			
	9.1	The primary analysis will include covariate adjustment of the minimisation variable. Since the SAP was written, there has been a change in practice with the standard now for analyses to be adjusted for the minimisation variables included in the randomisation process.			
	9.3	Text in SAP v1.0 on handling missing data for the secondary endpoints based on continuous data deleted. These outcomes are being analysed using repeated measures methods which can accommodate missing data.			
	9.5	Analysis methods for primary outcome changed to reflect the use of regression models to adjust for the minimisation variables in the analysis.			

	9.6<br><br>9.10	Intervention effect to be expressed as a risk difference and relative risk, rather than an odds ratio.<br><br>Analysis methods changed to reflect the use of regression models to adjust for the minimisation variables in the analysis.<br><br>Planned per-protocol analysis removed as considered uninformative now the primary outcome changed to any URTI-related relapse during the 12 months rather than assessing for relapse following first URTI, meaning for those who do not experience an URTI-related relapse during the 12 months which URTI is used for the assessment of adherence. Adherence to study medication is being assessed and summarised in a descriptive manner as described in section 7.2.			

**Abbreviations & Definitions**	
**Abbreviation / Acronym**	**Meaning**
BCTU	Birmingham Clinical Trials Unit
CONSORT	Consolidated Standards of Reporting Trials
DMC	Data Monitoring Committee
ISRCTN	International Standard Randomised Controlled Trial Number
ITT	Intention to Treat
SAE	Serious Adverse Event
SAP	Statistical Analysis Plan
SSNS	Steroid Sensitive Nephrotic Syndrome
SUSAR	Suspected Unexpected Serious Adverse Reaction
TSC	Trial Steering Committee
URTI	Upper Respiratory Tract Infection
**Term**	**Definition**
International Standard Randomised Controlled Trial Number	A clinical trial registry
Protocol	Document that details the rationale, objectives, design, methodology and statistical considerations of the study
Randomisation	The process of assigning trial subjects to intervention or control groups using an element of chance to determine the assignments in order to reduce bias.
Statistical Analysis Plan	Pre-specified statistical methodology documented for the trial, either in the protocol or in a separate document.

TABLE OF CONTENTS

1. Introduction 7
2. Background and rationale 7
3. Trial objectives 7
4. Trial methods 8
4.1. Trial design 8
4.2. Trial interventions 8
4.3. Primary outcome measure 8
4.4. Secondary outcome measures 8
4.5. Timing of outcome assessments 9
4.6. Randomisation 9
4.7. Sample size 9
4.8. Framework 9
4.9. Interim analyses and stopping guidance 10
4.10. Internal Pilot Progression Rules 10
4.11. Timing of final analysis 10
4.12. Timing of other analyses 10
4.13. Trial comparisons 10
5. Statistical Principles 10
5.1. Confidence intervals and p-values 10
5.2. Adjustments for multiplicity 10
5.3. Analysis populations 10
5.4. Definition of adherence 11
5.5. Handing protocol deviations 11
5.6. Unblinding 11
6. Trial population 11
6.1. Recruitment 11
6.2. Baseline characteristics 12
7. Intervention(s) 12
7.1. Description of the intervention(s) 12
7.2. Adherence to allocated intervention 12
8. Protocol deviations 12
9. Analysis methods 12
9.1. Covariate adjustment 12
9.2. Distributional assumptions and outlying responses 13
9.3. Handling missing data 13
9.4. Data manipulations 13
9.5. Analysis methods – primary outcome(s) 25
9.6. Analysis methods – secondary outcomes 25
9.7. Analysis methods – exploratory outcomes and analyses 26
9.8. Safety data 26
9.9. Planned subgroup analyses 26
9.10. Sensitivity analyses 26
10. Analysis of sub-randomisations 27
11. Health economic analysis 27
12. Statistical software 27
13. References 27
Appendix A: Deviations from SAP 28
Appendix B: Trial schema 28

1. Introduction

This document is the Statistical Analysis Plan (SAP) for the PREDNOS 2 trial, and should be read in conjunction with the current trial protocol. This SAP details the proposed analyses and presentation of the data for the main paper(s) reporting the results for PREDNOS 2.

The results reported in these papers will follow the strategy set out here. Subsequent analyses of a more exploratory nature will not be bound by this strategy, though they are expected to follow the broad principles laid down here. The principles are not intended to curtail exploratory analysis (e.g. to decide cut-points for categorisation of continuous variables), nor to prohibit accepted practices (e.g. transformation of data prior to analysis), but they are intended to establish rules that will be followed, as closely as possible, when analysing and reporting data.

Any deviations from this SAP will be described and justified in the final report or publication of the trial (using a table as shown in Appendix A). The analysis will be carried out by an appropriately qualified statistician, who should ensure integrity of the data during their data cleaning processes.

2. Background and rationale

The background and rationale for the trial are outlined in detail in the protocol. In brief, presenting episodes and relapses of nephrotic syndrome in children with steroid sensitive nephrotic syndrome (SSNS) are treated with high dose prednisolone. At least 50% of relapses are precipitated by viral upper respiratory tract infection (URTI), possibly mediated through release of cytokines.[1] Furthermore, in children with frequently relapsing SSNS, the development of an URTI results in relapse in over 50% of instances. Given these strong links between viral URTI and relapse, and the morbidity and cost associated with relapse and its treatment, it is logical that attempts are made to ameliorate the URTI-driven process.

3. Trial objectives

The primary objective is to determine whether a six day course of oral prednisolone given at the time of URTI reduces the incidence of first URTI-related relapse in children with relapsing SSNS.

Secondary objectives are to determine whether a six day course of oral prednisolone given at the time of URTI:

i. Reduces the overall rate of URTI-related relapse of nephrotic syndrome
ii. Reduces the overall rate of (URTI-related and non URTI-related) relapse of nephrotic syndrome
iii. Reduces the cumulative dose of prednisolone received over the 12 month trial period
iv. Reduces the incidence and prevalence of adverse effects of prednisolone including behavioural abnormalities
v. Reduces the number of subjects undergoing escalation of background immunosuppressive therapy

vi. Increases the number of subjects undergoing reduction of background immunosuppressive therapy
vii. Is more cost-effective than standard therapy

4. Trial methods

4.1. Trial design

PREDNOS 2 is a multi-centre, prospective, double-blind, superiority, parallel group, phase III randomised controlled trial. See Appendix B for trial schema.

Participants will be recruited from secondary care.

4.2. Trial interventions

Active treatment group: Daily prednisolone for a total of 6 days commenced within 24 hours of the criteria for an URTI being met.

Control group: Daily matched placebo for a total of 6 days commenced within 24 hours of the criteria for an URTI have being met.

See protocol for detailed information on the dosing of the study drug.

4.3. Primary outcome measure

The primary outcome is first URTI-related relapse of nephrotic syndrome during the 12 month follow up period.

Relapse is defined as Albustix positive proteinuria (+++ or greater) for 3 consecutive days or the presence of generalised oedema plus 3+ proteinuria.

An URTI-related relapse is defined as a relapse occurring within 14 days of the development of an URTI.

4.4. Secondary outcome measures

The secondary outcomes are as follows:

i. Rate of URTI-related relapse of nephrotic syndrome
ii. Rate of relapse (URTI-related and non URTI-related) of nephrotic syndrome
iii. Cumulative dose of prednisolone (mg/kg and mg/m^2) received over the 12 month period
iv. Incidence of serious adverse events (SAEs)
v. Incidence of adverse effects of prednisolone including assessment of behaviour using the Achenbach Child Behaviour Checklist
vi. Incidence of escalation of background immunosuppressive therapy
vii. Incidence of reduction of background immunosuppressive therapy

viii. Quality of life using the PedsQL (0=worst, 100=best). See Section 9.4 for questionnaire scoring.
ix. Cost per relapse of nephrotic syndrome
x. Cost per QALY gained

The secondary outcomes measures ix and x relate to the health economic analysis and are not part of this SAP.

4.5. Timing of outcome assessments

The schedule of trial procedures and outcome assessments are given in the protocol section 4.12.

4.6. Randomisation

Participants will be randomised in a 1:1 ratio to either active treatment or placebo.

Randomisation will be performed centrally at the Birmingham Clinical Trials Unit (BCTU) using a minimisation algorithm incorporating the following factor:

- Treatment regimen subject is receiving at randomisation: no background immunosuppressive therapy; long term maintenance prednisolone therapy; long term maintenance prednisolone therapy plus other immunosuppressive therapies; other immunosuppressive therapies alone.

4.7. Sample size

A total of 360 subjects will be recruited into the trial (180 into each group).

In children with frequently relapsing SSNS, the development of an URTI results in relapse in around 50% of instances.[2] To detect an absolute difference of 17.5% (i.e. 35% proportional reduction) in URTI-related relapse rate (i.e. from 50% to 32.5%), with 80% power and alpha=0.05, requires 250 subjects in total. Allowing for 30% drop out (e.g. subject withdrawal or lost to follow up, or subject not having an URTI during the 12 month follow up period), will require recruitment of 360 subjects. We therefore propose to recruit 360 subjects, 180 to each group.

4.8. Framework

The objective of the trial is to test the superiority of one intervention to another.

The null hypothesis is that there is no difference in the number of children experiencing an URTI-related relapse between the intervention groups. The alternative hypothesis is that there is a difference between the groups.

4.9. Interim analyses and stopping guidance

A separate Data Monitoring Committee (DMC) reporting template will be drafted and agreed by the DMC including an agreement on which outcomes will be reported at interim analyses. The statistical methods stated in this SAP will be followed for the outcomes included in the DMC report, where possible.

Interim analyses of efficacy and safety are planned annually. The Haybittle-Peto approach will be used whereby all interim analyses use a difference of 3 standard errors (approximately p=0.002) as a stopping guideline. These interim analyses will be reviewed by the DMC on an annual basis or more frequently if required by the DMC or Trial Management Committee

4.10. Internal Pilot Progression Rules

Not Applicable.

4.11. Timing of final analysis

The final analysis for the trial will occur after the last patient randomised into the trial has reached 12 months follow up and the corresponding outcome data has been entered onto the trial database and validated as being ready for analysis. This is provided that the trial has not been stopped early for any reason (e.g. DMC advice or funding body request).

4.12. Timing of other analyses

Not Applicable.

4.13. Trial comparisons

All references in this document to 'group' refer to the active 6 day daily course of prednisolone group or to the placebo group.

5. Statistical Principles

5.1. Confidence intervals and p-values

All estimates of differences between groups will be presented with two-sided 95% confidence intervals, unless otherwise stated. P-values will be reported from two-sided tests at the 5% significance level.

5.2. Adjustments for multiplicity

No correction for multiple testing will be made.

5.3. Analysis populations

All primary analyses (primary and secondary outcomes including safety outcomes) will be based on the intention-to-treat (ITT) principle. Participants will be analysed in the intervention group to which they were randomised, and all participants shall be included whether or not they

received the allocated intervention following an URTI. This is to avoid any potential bias in the analysis.

The ITT population will not include all of the randomised population. Analyses will be based on a modified ITT population which will include only those participants who had an URTI over the 12 month follow up period. Excluding those participants who did not have an URTI will result in no bias as taking the study medication is conditional on them experiencing an URTI, and this should by chance be balanced between the two groups.

5.4. Definition of adherence

Adherence to allocated intervention will be monitored using the parent-reported tablet count. This is instead of using the returned pot tablet count because the tablet count is just a check of what they have taken compared to what they have said they have taken, rather than whether or not they have been adherent to the intervention. This is further complicated by reissued pots, pots that have not been returned, dropped tablets etc. Good adherence is defined as participants taking 80-120% of their required tablets.

5.5. Handing protocol deviations

A protocol deviation is defined as a failure to adhere to the protocol such as errors in applying the inclusion/exclusion criteria, the incorrect intervention being given, incorrect data being collected or measured, follow-up visits outside the visit window or missed follow-up visits. We will apply a strict definition of the ITT principle and will include all participants as per the modified ITT population described in section 5.3 for the primary analyses, in some form, regardless of deviation from the protocol.[3] This includes participants who were randomised but later found to not meet the inclusion or exclusion criteria. This does not include those participants who have specifically withdrawn consent for the use of their data in the first instance; however these outcomes will be explored as per other missing responses.

5.6. Unblinding

This is a double-blind trial with the participants and clinicians remaining blind to treatment allocation. To maintain blinding, participants randomised to the control group receive the same number of study tablets that they would have received in the active treatment group through supplemental placebo tablets. The Trial Statistician has been unblinded to the intervention code for all of the interim analyses, and presented unblinded analyses to the DMC.

6. Trial population

6.1. Recruitment

A flow diagram (as recommended by CONSORT[4]) will be produced to describe the participant flow through each stage of the trial. This will include information on the number (with reasons) of losses to follow-up (drop-outs and withdrawals) over the course of the trial.

6.2. Baseline characteristics

The trial population will be tabulated with categorical data summarised by number of participants, counts and percentages, and continuous data summarised by the number of participants, mean and standard deviation if deemed to be normally distributed or number of participants, median and interquartile range if data appear skewed, and ranges if appropriate. Tests of statistical significance will not be undertaken, nor confidence intervals presented.[5]

7. Intervention(s)

7.1. Description of the intervention(s)

Not Applicable.

7.2. Adherence to allocated intervention

Adherence to allocated intervention will be summarised descriptively. At each follow-up visit, if the participant had an URTI the following questions are completed:

- Did the parent commence their child on study medication at time of URTI?
- If study medication not commenced, please state reason
- Date study medication commenced (note: study medication should be commenced within 24 hours of the criteria for an URTI being met).
- Was the whole 6 day course of study medication taken?
- If no, how many days were missed?
- Was the 6 day course prematurely discontinued?
- If study medication discontinued, please state reason

These questions will be summarised and presented by group.

8. Protocol deviations

Frequencies and percentages by group will be tabulated for the protocol deviations.

9. Analysis methods

9.1. Covariate adjustment

In the first instance, intervention effects between groups for all outcomes will be adjusted for the minimisation parameter listed in section 4.6. Other covariate adjustment will be baseline values for parameters where available (e.g. an analysis of questionnaire scores will also include the baseline score as a covariate in the model).

If covariate adjustment is not possible (e.g. the model does not converge), the minimisation variable covariate will be removed from the model, but where applicable adjustment for baseline values will still be made. If the model still does not converge, unadjusted estimates will be produced, and it will be made clear in the final report why this occurred (e.g. not possible due to low event rate/lack of model convergence).

If the log-binomial model fails to converge, a Poisson regression model with robust standard errors will be used to estimate the same parameters[6]. If this also fails to converge, unadjusted estimates will be produced from the log-binomial model. It will be made clear in the final report why this occurred (e.g. not possible due to low event rate/lack of model convergence).

9.2. Distributional assumptions and outlying responses

Distributional assumptions (e.g. normality of regression residuals for continuous outcomes) will be assessed visually prior to analysis; although in the first instance the proposed primary method of estimation in this analysis plan will be followed. If responses are considered to be particularly skewed and/or distributional assumptions violated, the impact of this will be examined through sensitivity analysis; this will consist of transformation of responses prior to analysis (e.g. log transformation) in the first instance. If extreme values are apparent and considered to be affecting the integrity of the analysis, a sensitivity analysis consisting of removing the outlying response(s) and repeating the analysis will be performed. Output from these analyses, if performed, will be described and presented alongside the original analysis (or included, e.g. in appendices) with the excluded values clearly labelled.

9.3. Handling missing data

In the first instance, analysis will be completed on received data only with every effort made to follow-up participants even after protocol violation to minimise any potential for bias. To examine the possible impact of missing data on the results, and to make sure we are complying with the intention-to-treat principle, sensitivity analysis will be performed on the primary outcome measure.[7] See section 9.10 for further details regarding sensitivity analyses.

9.4. Data manipulations

The Trial Statistician will derive all responses from the raw data recorded in the database:

Cumulative dose of prednisolone:

Prednisolone is recorded in both Part B1 and Part I of the follow-up form at each time-point. Part I dosing is recorded as free text so in order to use this data for the cumulative prednisolone doses, the Trial Statistician will recode all the free text as the numerical variables: dose and frequency where possible.

Then, in the first instance, clean data from Part B1 and Part I will be combined.

Data can then be checked across both parts of the form to see whether there are any duplicates in doses received. Duplicated doses will be deleted.

Any dosing regimens that are ongoing on one form will be checked against the following form for the stop date. Once a stop date has been obtained, the entry or entries where

the dose is ongoing will be collapsed, so that there is one dose regimen with a defined start and stop date.

There may be some instances where start date and/or stop date are missing as the sites had a rough idea of the treatment regimen but not of the dates e.g. tapering regimen following a relapse. In these cases it is more appropriate to check against the participants relapse history and received treatment in order to make sensible assumptions about the missing dates, than to not use the available data.

If a start date is pre-randomisation date, and the prednisolone regimen is on-going at point of randomisation, the start date will be replaced with the randomisation date so as to only capture prednisolone therapy during the trial.

Cumulative prednisolone dose will be based on those participants who complete the full 12 months trial follow-up. If the dose is ongoing at 12 months the dose will be truncated at 12 months (i.e. randomisation date + 12 months) to provide the cumulative prednisolone dose over the 12 month follow-up period.

For those in the active treatment group, the trial treatment will be included in the cumulative prednisolone. Where tablet count is available, the tablet count will be converted to mg, and then added to the cumulative prednisolone for that participant. If tablet count is not available, then other information on adherence to the trial medication will be used instead to provide an estimated tablet count or dose.

Since drug data is notoriously difficult to collect and analyse, as a check of the statistical code, a manual check and calculation of cumulative prednisolone for 20 participants will be undertaken.

Incidence of Escalation/Reduction of Background Immunosuppressant Therapy:

The background treatment regimen is collected at each of the follow up time points. Data on second line immunosuppressants is also collected (ciclosporin, tacrolimus, levamisole, mycophenolate mofetil, mycophenolate sodium, azathioprine, cyclophosphamide and rituximab). The background treatment regime is categorised into the following four categories:

1. No long-term alternate day prednisolone or other long-term immunosuppressive therapies
2. On long-term alternate day prednisolone only
3. On both long-term alternate day prednisolone and other long-term immunosuppressive therapies
4. On other long-term immunosuppressive therapies only

The background treatment regimen and second line immunosuppressant information will be used in the following way to calculate escalation/reduction of background immunosuppressant therapy:

Escalation will be computed based as per the following criteria –

- If a patient is in category 1 from the background treatment regime at previous visit and then at a following time-point is in category 2, 3 or 4 of the background treatment regime then this is considered as an escalation.
- If a patient is in category 2 from the background treatment regime at previous visit and then at a following time-point is in category 3 or 4 of the background treatment regime then this is also considered as an escalation.
- If a patient is in category 4 from the background treatment regime at previous visit and then at a following time-point is in category 3 of the background treatment regime then this is also considered as an escalation.
- If the participant is not on cyclophosphamide or rituximab at previous visit but are then given cyclophosphamide or rituximab at the following time-point then this is also considered as an escalation.
- If participant has an addition of any immunosuppressant (e.g. addition of ciclosporin) in between follow up visits then this is considered an escalation.
- If the participant has a change in medication from Levamisole to either Tacrolimus, Ciclosporin or Mycophenolate then this is also considered as an escalation.

Reduction will be computed based as per the following criteria –

- If a patient is in category 3 from the background treatment regime at previous visit and then at a following time-point is in category 4, 2 or 1 of the background treatment regime then this is considered as a reduction.
- If a patient is in category 4 from the background treatment regime at previous visit and then at a following time-point is in category 2 or 1 of the background treatment regime then this is also considered as a reduction.
- If a patient is in category 2 from the background treatment regime at previous visit and then at a following time-point is in category 1 of the background treatment regime then this is also considered as a reduction.
- If participant on an immunosuppressant (except cyclophosphamide or rituximab) at one time point, but is no longer on it at the following time point then this is also considered as a reduction.

PedsQL for ages 2-4 years

The PedsQL response scales are coded as follows:

Physical Functioning (problems with…)

- Walking: Never=100, Almost Never=75, Sometimes=50, Often=25, Almost Always=0

- Running: : Never=100, Almost Never=75, Sometimes=50, Often=25, Almost Always=0
- Participating in active play or exercise: Never=100, Almost Never=75, Sometimes=50, Often=25, Almost Always=0
- Lifting something heavy: Never=100, Almost Never=75, Sometimes=50, Often=25, Almost Always=0
- Bathing: Never=100, Almost Never=75, Sometimes=50, Often=25, Almost Always=0
- Helping to pick up his or her toys: Never=100, Almost Never=75, Sometimes=50, Often=25, Almost Always=0
- Having hurts or aches: Never=100, Almost Never=75, Sometimes=50, Often=25, Almost Always=0
- Low energy level: Never=100, Almost Never=75, Sometimes=50, Often=25, Almost Always=0

Emotional Functioning (problems with…)

- Feeling afraid or scared: Never=100, Almost Never=75, Sometimes=50, Often=25, Almost Always=0
- Feeling sad or blue: Never=100, Almost Never=75, Sometimes=50, Often=25, Almost Always=0
- Feeling angry: Never=100, Almost Never=75, Sometimes=50, Often=25, Almost Always=0
- Trouble sleeping: Never=100, Almost Never=75, Sometimes=50, Often=25, Almost Always=0
- Worrying: Never=100, Almost Never=75, Sometimes=50, Often=25, Almost Always=0

Social Functioning (problems with…)

- Playing with other children: Never=100, Almost Never=75, Sometimes=50, Often=25, Almost Always=0
- Other kids not wanting to play with him or her: Never=100, Almost Never=75, Sometimes=50, Often=25, Almost Always=0

- Getting teased by other children: Never=100, Almost Never=75, Sometimes=50, Often=25, Almost Always=0

- Not able to do things that other children his or her age can do: Never=100, Almost Never=75, Sometimes=50, Often=25, Almost Always=0

- Keeping up when playing with other children: Never=100, Almost Never=75, Sometimes=50, Often=25, Almost Always=0

School Functioning (problems with…)

- Doing the same school activities as peers: Never=100, Almost Never=75, Sometimes=50, Often=25, Almost Always=0

- Missing school/daycare because of not feeling well: Never=100, Almost Never=75, Sometimes=50, Often=25, Almost Always=0

- Missing school/daycare to go to the doctor or hospital: Never=100, Almost Never=75, Sometimes=50, Often=25, Almost Always=0

The scores are derived as follows:

  - Mean physical functioning score=sum of all physical function scores / (number of items answered)

  - Mean emotional functioning=sum of all emotional functioning scores / (number of items answered)

  - Mean social functioning=sum of all social functioning scores / (number of items answered)

  - Mean school functioning=sum of all school functioning scores / (number of items answered)

NOTE: If more than 50% of items in the scale are missing, a domain score should not be computed for that domain.

Psychosocial Health Summary Score = sum of items in emotional functioning, social functioning, and school functioning scales / (number of items answered)

Physical Health Summary Score = Physical Functioning Scale score (as shown above)

Total score: Sum of all the items / (number of items answered)

PedsQL for ages 5-7 years

The PedsQL response scales are coded as follows:

Physical Functioning (problems with…)

- Walking more than one block: Never=100, Almost Never=75, Sometimes=50, Often=25, Almost Always=0
- Running: Never=100, Almost Never=75, Sometimes=50, Often=25, Almost Always=0
- Participating in sports activity or exercise: Never=100, Almost Never=75, Sometimes=50, Often=25, Almost Always=0
- Lifting something heavy: Never=100, Almost Never=75, Sometimes=50, Often=25, Almost Always=0
- Taking a bath or shower by him or herself: Never=100, Almost Never=75, Sometimes=50, Often=25, Almost Always=0
- Doing chores, like picking up his or her toys: Never=100, Almost Never=75, Sometimes=50, Often=25, Almost Always=0
- Having hurts or aches: Never=100, Almost Never=75, Sometimes=50, Often=25, Almost Always=0
- Low energy level: Never=100, Almost Never=75, Sometimes=50, Often=25, Almost Always=0

Emotional Functioning (problems with…)

- Feeling afraid or scared: Never=100, Almost Never=75, Sometimes=50, Often=25, Almost Always=0
- Feeling sad or blue: Never=100, Almost Never=75, Sometimes=50, Often=25, Almost Always=0
- Feeling angry: Never=100, Almost Never=75, Sometimes=50, Often=25, Almost Always=0
- Trouble sleeping: Never=100, Almost Never=75, Sometimes=50, Often=25, Almost Always=0

- Worrying about what will happen to him or her: Never=100, Almost Never=75, Sometimes=50, Often=25, Almost Always=0

Social Functioning (problems with…)

- Getting along with other children: Never=100, Almost Never=75, Sometimes=50, Often=25, Almost Always=0

- Other kids not wanting to be his or her friend: Never=100, Almost Never=75, Sometimes=50, Often=25, Almost Always=0

- Getting teased by other children: Never=100, Almost Never=75, Sometimes=50, Often=25, Almost Always=0

- Not able to do things that other children his or her age can do: Never=100, Almost Never=75, Sometimes=50, Often=25, Almost Always=0

- Keeping up when playing with other children: Never=100, Almost Never=75, Sometimes=50, Often=25, Almost Always=0

School Functioning (problems with…)

- Paying attention in class: Never=100, Almost Never=75, Sometimes=50, Often=25, Almost Always=0

- Forgetting things: Never=100, Almost Never=75, Sometimes=50, Often=25, Almost Always=0

- Keeping up with school activities: Never=100, Almost Never=75, Sometimes=50, Often=25, Almost Always=0

- Missing school because of not feeling well: Never=100, Almost Never=75, Sometimes=50, Often=25, Almost Always=0

- Missing school to go to the doctor or hospital: Never=100, Almost Never=75, Sometimes=50, Often=25, Almost Always=0

The scores are derived as follows:

Mean Physical Functioning=sum of all physical function scores / (number of items answered)

Emotional Functioning=sum of all emotional functioning scores / (number of items answered)

Social Functioning=sum of all social functioning scores / (number of items answered)

School Functioning=sum of all school functioning scores / (number of items answered)

NOTE: If more than 50% of items in the scale are missing, a domain score should not be computed for that domain.

Psychosocial Health Summary Score = sum of items in emotional functioning, social functioning, and school functioning scales / (number of items answered)

Physical Health Summary Score = Physical Functioning Scale score (as shown above)

Total score: Sum of all the items / (number of items answered)

PedsQL for ages 8-12 years

Physical Functioning (problems with…)

- Walking more than one block: Never=100, Almost Never=75, Sometimes=50, Often=25, Almost Always=0
- Running: Never=100, Almost Never=75, Sometimes=50, Often=25, Almost Always=0
- Participating in sports activity or exercise: Never=100, Almost Never=75, Sometimes=50, Often=25, Almost Always=0
- Lifting something heavy: Never=100, Almost Never=75, Sometimes=50, Often=25, Almost Always=0
- Taking a bath or shower by him or herself: Never=100, Almost Never=75, Sometimes=50, Often=25, Almost Always=0
- Doing chores around the house: Never=100, Almost Never=75, Sometimes=50, Often=25, Almost Always=0
- Having hurts or aches: Never=100, Almost Never=75, Sometimes=50, Often=25, Almost Always=0
- Low energy level: Never=100, Almost Never=75, Sometimes=50, Often=25, Almost Always=0

Emotional Functioning (problems with…)

- Feeling afraid or scared: Never=100, Almost Never=75, Sometimes=50, Often=25, Almost Always=0

- Feeling sad or blue: Never=100, Almost Never=75, Sometimes=50, Often=25, Almost Always=0

- Feeling angry: Never=100, Almost Never=75, Sometimes=50, Often=25, Almost Always=0

- Trouble sleeping: Never=100, Almost Never=75, Sometimes=50, Often=25, Almost Always=0

- Worrying about what will happen to him or her: Never=100, Almost Never=75, Sometimes=50, Often=25, Almost Always=0

Social Functioning (problems with…)

- Getting along with other children: Never=100, Almost Never=75, Sometimes=50, Often=25, Almost Always=0

- Other kids not wanting to be his or her friend: Never=100, Almost Never=75, Sometimes=50, Often=25, Almost Always=0

- Getting teased by other children: Never=100, Almost Never=75, Sometimes=50, Often=25, Almost Always=0

- Not able to do things that other children his or her age can do: Never=100, Almost Never=75, Sometimes=50, Often=25, Almost Always=0

- Keeping up when playing with other children: Never=100, Almost Never=75, Sometimes=50, Often=25, Almost Always=0

School Functioning (problems with…)

- Paying attention in class: Never=100, Almost Never=75, Sometimes=50, Often=25, Almost Always=0

- Forgetting things: Never=100, Almost Never=75, Sometimes=50, Often=25, Almost Always=0

- Keeping up with schoolwork: Never=100, Almost Never=75, Sometimes=50, Often=25, Almost Always=0

- Missing school because of not feeling well: Never=100, Almost Never=75, Sometimes=50, Often=25, Almost Always=0

- Missing school to go to the doctor or hospital: Never=100, Almost Never=75, Sometimes=50, Often=25, Almost Always=0

The scores are derived as follows:

Mean Physical Functioning=sum of all physical function scores / (number of items answered)

Emotional Functioning=sum of all emotional functioning scores / (number of items answered)

Social Functioning=sum of all social functioning scores / (number of items answered)

School Functioning=sum of all school functioning scores / (number of items answered)

NOTE: If more than 50% of items in the scale are missing, a domain score cannot be computed for that domain.

Psychosocial Health Summary Score = sum of items in emotional functioning, social functioning, and school functioning scales / (number of items answered)

Physical Health Summary Score = Physical Functioning Scale score (as shown above)

Total score: Sum of all the items / (number of items answered)

<u>PedsQL for ages 13-18 years</u>

<u>Physical Functioning (problems with…)</u>

- Walking more than one block: Never=100, Almost Never=75, Sometimes=50, Often=25, Almost Always=0

- Running: Never=100, Almost Never=75, Sometimes=50, Often=25, Almost Always=0

- Participating in sports activity or exercise: Never=100, Almost Never=75, Sometimes=50, Often=25, Almost Always=0

- Lifting something heavy: Never=100, Almost Never=75, Sometimes=50, Often=25, Almost Always=0

- Taking a bath or shower by him or herself: Never=100, Almost Never=75, Sometimes=50, Often=25, Almost Always=0

- Doing chores around the house: Never=100, Almost Never=75, Sometimes=50, Often=25, Almost Always=0

- Having hurts or aches: Never=100, Almost Never=75, Sometimes=50, Often=25, Almost Always=0

- Low energy level: Never=100, Almost Never=75, Sometimes=50, Often=25, Almost Always=0

Emotional Functioning (problems with…)

- Feeling afraid or scared: Never=100, Almost Never=75, Sometimes=50, Often=25, Almost Always=0

- Feeling sad or blue: Never=100, Almost Never=75, Sometimes=50, Often=25, Almost Always=0

- Feeling angry: Never=100, Almost Never=75, Sometimes=50, Often=25, Almost Always=0

- Trouble sleeping: Never=100, Almost Never=75, Sometimes=50, Often=25, Almost Always=0

- Worrying about what will happen to him or her: Never=100, Almost Never=75, Sometimes=50, Often=25, Almost Always=0

Social Functioning (problems with…)

- Getting along with other teens: Never=100, Almost Never=75, Sometimes=50, Often=25, Almost Always=0

- Other teens not wanting to be his or her friend: Never=100, Almost Never=75, Sometimes=50, Often=25, Almost Always=0

- Getting teased by other teens: Never=100, Almost Never=75, Sometimes=50, Often=25, Almost Always=0

- Not able to do things that other teens his or her age can do: Never=100, Almost Never=75, Sometimes=50, Often=25, Almost Always=0

- Keeping up when playing with other teens: Never=100, Almost Never=75, Sometimes=50, Often=25, Almost Always=0

School Functioning (problems with…)

- Paying attention in class: Never=100, Almost Never=75, Sometimes=50, Often=25, Almost Always=0

- Forgetting things: Never=100, Almost Never=75, Sometimes=50, Often=25, Almost Always=0

- Keeping up with schoolwork: Never=100, Almost Never=75, Sometimes=50, Often=25, Almost Always=0

- Missing school because of not feeling well: Never=100, Almost Never=75, Sometimes=50, Often=25, Almost Always=0

- Missing school to go to the doctor or hospital: Never=100, Almost Never=75, Sometimes=50, Often=25, Almost Always=0

The scores are derived as follows:

Mean Physical Functioning=sum of all physical function scores / (number of items answered)

Emotional Functioning=sum of all emotional functioning scores / (number of items answered)

Social Functioning=sum of all social functioning scores / (number of items answered)

School Functioning=sum of all school functioning scores / (number of items answered)

NOTE: If more than 50% of items in the scale are missing, a domain score should not be computed for that domain.

Psychosocial Health Summary Score = sum of items in emotional functioning, social functioning, and school functioning scales / (number of items answered)

Physical Health Summary Score = Physical Functioning Scale score (as shown above)

Total score: Sum of all the items / (number of items answered)

9.5. Analysis methods – primary outcome(s)

The outcome will be presented as number of URTI-related relapses along with percentage using the modified ITT cohort.

An adjusted risk difference and relative risk along with the respective 95% confidence intervals will be estimated using binomial models with the identity and log link respectively. Statistical significance of the treatment group parameter will be determined from the p-value generated by the model. The number needed to treat will also be provided.

See section 9.1 for covariate adjustment and model convergence.

9.6. Analysis methods – secondary outcomes

The number of participants experiencing any relapse (i.e. both URTI-related and non URTI-related relapses), the number of participants having an escalation of background immunosuppressant therapy and the number of participants having a reduction of background immunosuppressant therapy will be presented and analysed as per the primary outcome measure.

The rate of URTI-related relapses and rate of any relapse will be presented in the first instance as numbers of participants who have a relapse with the respective percentage, and in the second instance as a median and IQR. An adjusted incidence rate ratio with 95% confidence interval will be estimated using a Poisson regression model. An offset for the length of time the participant was in the trial will be included in the model. Statistical significance of the treatment group parameter will be determined from the p-value generated by the model.

The cumulative dose of prednisolone received over the 12 month period will be presented as means with standard deviations and ranges. Adjusted mean differences along with 95% confidence intervals will be estimated using a linear regression model. Statistical significance of the treatment group parameter will be determined from the p-value generated by the model.

Adverse effect data will be tabulated at each time point (3, 6, 9 and 12 months) with statistical significance determined by a chi-squared or Fisher's exact test (as appropriate). We will also assess whether or not each adverse event occurred over the whole trial period by assessing all the 3, 6, 9 and 12 month data collectively.

Quality of life measures are taken at multiple time points, as is the Achenbach Behaviour Checklist Scores. Means and standard deviations will be summarised at each time point. Data will be analysed using mixed effect repeated measures models with the minimisation variable and the baseline questionnaire score included in the model as covariates. Time will be included as a continuous variable in the model. In the initial model, a treatment by time cross-term will be included in the model. If this is not significant, it will be considered that the treatment effect

is constant over time, and models without the treatment by time cross-term will be fitted. Adjusted mean differences between groups will be presented alongside the respective 95% confidence interval.

9.7. Analysis methods – exploratory outcomes and analyses

Any data that does not form a pre-specified outcome will be presented using simple summary statistics by intervention group (i.e. numbers and percentages for binary data and means (or medians) and standard deviations (or inter-quartile ranges) for continuous normal (or non-normal) data.

9.8. Safety data

The number and percentage of participants experiencing any adverse events, serious adverse events (SAEs) and suspected unexpected serious adverse reactions (SUSARs) will be presented by intervention group. Statistical significance will be determined by a chi-squared or Fisher's exact test (as appropriate). A table listing the SAEs including information on the nature of the event and whether or not it was deemed related to the trial treatment will be provided.

9.9. Planned subgroup analyses

Interpretation of subgroup analysis will be treated with caution (output will be treated as exploratory rather than definitive[9]). Analysis will be limited to the primary outcome only, and the following subgroups based on Background Treatment Regimen at Randomisation:

- No background immunosuppressive therapy; long term maintenance prednisolone therapy; long term maintenance prednisolone therapy plus other immunosuppressive therapies; other immunosuppressive therapies alone.
- On prednisolone vs. not on prednisolone
- On other immunosuppressant vs. not on other immunosuppressant

The effects of these subgroups will be examined by including a treatment group by subgroup interaction parameter in the final binomial model.

9.10. Sensitivity analyses

Sensitivity analyses will be limited to the primary outcome and will consist of:

- URTI-related relapse (Worse) / no URTI-related relapse (Best) and no URTI-related relapse (Best) / URTI-related relapse (Worse) for active and placebo groups respectively.

As an additional analysis, the number of URTI and non-URTI related relapses along with the number of participants who had a relapse in the modified ITT cohort will be compared to the number of non-URTI related relapses in that of the excluded cohort. It would be anticipated that those who have been excluded experience fewer relapses on the basis that they did not experience an URTI. However, if the numbers are similar it might be the case that some URTIs went unreported in this excluded population.

10. Analysis of sub-randomisations

Not Applicable.

11. Health economic analysis

As indicated in the protocol there will also be an economic analysis. The details of this analysis are documented separately.

12. Statistical software

Statistical analysis will be undertaken in the following statistical software packages: SAS software and Stata.

13. References

1. MacDonald N, Wolfish N, Maclaine P, et al. Role of respiratory viruses in exacerbations of primary nephrotic syndrome. J Paediatr 1986; 108:378-82.
2. Abeyagunawardena AS, Trompeter RS. Increasing the dose of prednisolone during viral infection reduced the risk of relapse in nephrotic syndrome: a randomised controlled trial. Arch Dis Child 2008;93:226-8.
3. Gupta SK. Intention-to-treat concept: A review. Perspect Clin Res. 2011;2(3):109-112.
4. Schulz KF, Altman DG, Moher D, for the CONSORT Group. CONSORT 2010 Statement: updated guidelines for reporting parallel group randomised trials. BMJ. 2010;340:c332.
5. Altman DG, Dore CJ. Randomisation and baseline comparisons in clinical trials. Lancet. 1990;335:149–53.
6. Zou G. A modified Poisson regression approach to prospective studies with binary data. Am J Epidemiol. 2004;159(7):702-6.
7. White IR, Horton NJ, Carpenter J, Pocock SJ. Strategy for intention to treat analysis in randomised trials with missing outcome data. BMJ. 2011;342:d40.
8. THE EUROQOL GROUP. (1990) EuroQol - a new facility for the measurement of health-related quality of life. *Health Policy*, 16, 199-208
9. 7. Wand R, Lagakos SW, Ware JH, Hunter DJ, Drazen JM. Reporting of subgroups analyses in clinical trials. NEJM. 2007;357:2189-94.

Appendix A: Deviations from SAP

This report below follows the statistical analysis plan dated <insert effective date of latest SAP> apart from following:

Section of report not following SAP	Reason
<insert section >	<insert, e.g. exploratory analyses request by TMG>

Appendix B: Trial schema

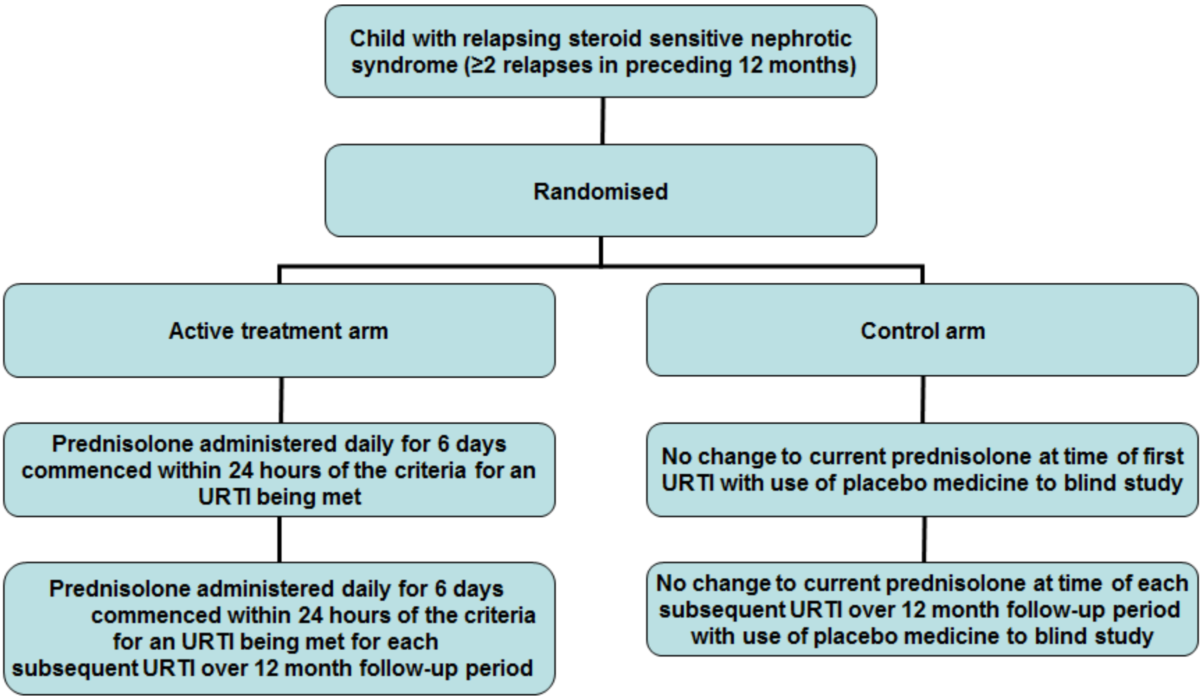

Supplement: Supplement 1. — Trial Protocol and Statistical Analysis Plan [file jamapediatr-e215189-s001.pdf]
