# Supplementary material for: Evaluation of Daily Low-Dose Prednisolone During Upper Respiratory Tract Infection to Prevent Relapse in Children With Relapsing Steroid-Sensitive Nephrotic Syndrome: The PREDNOS 2 Randomized Clinical Trial
Source: JAMA Pediatr. 2021 Dec 20;176(3):1–8. doi: 10.1001/jamapediatrics.2021.5189 (PMC8689426; doi:10.1001/jamapediatrics.2021.5189)
Supplement: Supplement 2. — eTable 1. Sensitivity Analyses for Primary Outcome Measures eTable 2. Adverse Events eTable 3. Achenbach Child Behavior Checklist Scores eTable 4. Quality of Life Using the PEDSQL eTable 5. Rationalisation of Ethnic Groups Following Self-reported Ethnicity eFigure. Subgroup Analyses for Primary Outcome Measure [file jamapediatr-e215189-s002.pdf]

Supplementary Online Content

Christian MT, Webb NJA, Mehta S, et al. Evaluation of daily low-dose prednisolone during upper respiratory tract infection to prevent relapse in children with relapsing steroid-sensitive nephrotic syndrome: the PREDNOS 2 randomized clinical trial. *JAMA Pediatr.* Published online December 20, 2021. doi:10.1001/jamapediatrics.2021.5189

eTable 1. Sensitivity Analyses for Primary Outcome Measures

eTable 2. Adverse Events

eTable 3. Achenbach Child Behavior Checklist Scores

eTable 4. Quality of Life Using the PEDSQL

eTable 5. Rationalisation of Ethnic Groups Following Self-reported Ethnicity

eFigure. Subgroup Analyses for Primary Outcome Measure

This supplementary material has been provided by the authors to give readers additional information about their work.

© 2021 Christian MT et al. *JAMA Surgery*.

eTable 1. Sensitivity Analyses for Primary Outcome Measure

	Prednisolone N=134	Placebo N=137	Adjusted Treatment Effect	95% Confidence Interval	P-value
**Primary Analysis**	**N=131**	**N=131**			
No	75 (57.3%)	73 (55.7%)	RD = -0.024	-0.142, 0.095	0.70
Yes	56 (42.7%)	58 (44.3%)	RR = 0.96	0.74, 1.26	-
**Sensitivity Analysis #1**	**N=134**	**N=136**			
No	78 (58.2%)	78 (57.4%)	RD = -0.014	-0.131, 0.102	0.81
Yes	56 (41.8%)	58 (42.6%)	RR = 0.99	0.75, 1.30	-
**Sensitivity Analysis #2**	**N=134**	**N=137**			
No	78 (58.2%)	73 (53.3%)	RD = -0.055	-0.171, 0.061	0.35
Yes	56 (41.8%)	64 (46.7%)	RR = 0.90	0.69, 1.16	-
**Sensitivity Analysis #3**	**N=134**	**N=137**			
No	75 (56.0%)	79 (57.7%)	RD = 0.012	-0.105, 0.129	0.84
Yes	59 (44.0%)	58 (42.3%)	RR = 1.04	0.80, 1.36	-

* Adjusting for the background therapy at baseline

RD=Risk Difference; a negative risk difference favours the Prednisolone Arm

RR=Risk Ratio; a risk ratio less than 1 favours the Prednisolone Arm

URTI=Upper respiratory tract infection

Sensitivity Analysis #1: One patient in the placebo arm had an URTI but no information was provided on whether they had an URTI-related relapse or not, so they are classed as missing. Three patients in the prednisolone arm and five patients in the placebo arm did not complete the 12-month follow-up and did not report an URTI-related relapse for any time-points where they provided data. These were classed as missing in the primary analysis, but were counted as 'No URTI-related relapse' in this sensitivity analysis.

Sensitivity Analysis #2: Assuming best-case (No URTI-related relapse) in the prednisolone arm and worse-case (URTI-related relapse) in the placebo arm for participants with missing data for the primary outcome.

© 2021 Christian MT et al. *JAMA Surgery*.

Sensitivity Analysis #3: Assuming worse-case (URTI-related relapse) in the prednisolone arm and best-case (No URTI-related relapse) in the placebo arm for participants with missing data for the primary outcome.

© 2021 Christian MT et al. *JAMA Surgery*.

eTable 2. Adverse Events

	Prednisolone	Placebo	P-value
Cushingoid facies	N=134	N=137	
No	93 (69.4%)	96 (70.1%)	0.9
Yes	41 (30.6%)	41 (29.9%)	
Striae	N=134	N=137	
No	125 (93.3%)	129 (94.2%)	0.8
Yes	9 (6.7%)	8 (5.8%)	
Hypertrichosis	N=134	N=137	
No	118 (88.1%)	119 (86.9%)	0.8
Yes	16 (11.9%)	18 (13.1%)	
Acne	N=134	N=137	
No	122 (91%)	126 (92%)	0.8
Yes	12 (9%)	11 (8%)	
Increased appetite	N=134	N=137	
No	66 (49.3%)	57 (41.6%)	0.2
Yes	68 (50.7%)	80 (58.4%)	
Poor behavior	N=134	N=137	
No	74 (55.2%)	63 (46%)	0.1
Yes	60 (44.8%)	74 (54%)	
Glycosuria	N=134	N=137	
Absent	123 (91.8%)	129 (94.2%)	0.4
Present	11 (8.2%)	8 (5.8%)	
Abdominal pain			
Absent	99 (73.9%)	104 (75.9%)	0.7
Present	35 (26.1%)	33 (24.1%)	

* P-value from chi-squared test

© 2021 Christian MT et al. *JAMA Surgery*.

eTable 3. Achenbach Child Behavior Checklist Scores

Achenbach		Prednisolone	Placebo	Adjusted Mean Difference (95% C.I) P-value	Treatment by time interaction p-value
**Total Problems T Score**					
**Baseline**	N	131	131	-1.02 (-2.91, 0.86) P=0.29	0.11
	Mean (SD)	50.2 [13.3]	50.9 [13.6]		
**3 months**	N	127	133		
	Mean (SD)	48 [13.7]	49.7 [14.2]		
**6 months**	N	117	123		
	Mean (SD)	47.4 [13.5]	48.1 [13.9]		
**9 months**	N	121	115		
	Mean (SD)	45.8 [13.8]	46.4 [13.2]		
**12 months**	N	118	121		
	Mean (SD)	45 [12.9]	47.8 [14.4]		
**Total Problems Total Score**					
**Baseline**	N	131	132	-1.43 (-5.00, 2.14) P=0.43	0.05
	Mean (SD)	30.2 [27.5]	31.5 [26.6]		
**3 months**	N	127	133		
	Mean (SD)	26.8 [28]	29.7 [29.5]		
**6 months**	N	119	124		
	Mean (SD)	24.9 [25.7]	26.5 [26.9]		
**9 months**	N	122	115		
	Mean (SD)	23.1 [24.6]	23.1 [24]		
**12 months**	N	119	121		
	Mean (SD)	20.7 [22.6]	26.1 [29.7]		

Achenbach score: higher scores are worse.

Means and standard deviations are presented unless otherwise specified.

A negative mean difference favours the Prednisolone Arm.

* Adjusting for the background therapy at baseline and baseline value.

© 2021 Christian MT et al. *JAMA Surgery*.

eTable 4. Quality Of Life Using the PEDSQL

PedsQL		Prednisolone	Placebo	Adjusted Mean Difference (95% C.I)<br><br>P-value	Treatment by time interaction p-value
**Physical Health Summary Score**					
**Baseline**	N<br>Mean (SD)	133<br>83.5 [20.2]	135<br>84.3 [16.9]	2.77 (-0.27, 5.80)<br><br>P=0.07	0.85
**3 months**	N<br>Mean (SD)	131<br>84.2 [19]	132<br>79.9 [20.8]		
**6 months**	N<br>Mean (SD)	124<br>84.5 [19]	124<br>83.5 [17.7]		
**9 months**	N<br>Mean (SD)	125<br>85.5 [18.2]	117<br>83.9 [19.9]		
**12 months**	N<br>Mean (SD)	124<br>85.6 [18.6]	124<br>82.2 [20.7]		
**Psychosocial Health Summary Score**					
**Baseline**	N<br>Mean (SD)	133<br>81 [16.8]	135<br>79.4 [15.5]	0.98 (-1.40, 3.36)<br><br>P=0.42	0.95
**3 months**	N<br>Mean (SD)	131<br>81.8 [15.8]	132<br>78.3 [15.2]		
**6 months**	N<br>Mean (SD)	124<br>82.1 [16.4]	124<br>81.5 [14.8]		
**9 months**	N<br>Mean (SD)	125<br>83.4 [15.3]	117<br>82.4 [14.6]		
**12 months**	N<br>Mean (SD)	124<br>84.8 [16]	124<br>81.7 [16.3]		
**Total Score**					
**Baseline**	N<br>Mean (SD)	133<br>81.9 [16.5]	135<br>81.2 [14.5]	1.52 (-0.83, 3.87)<br><br>P=0.20	0.98
**3 months**	N<br>Mean (SD)	131<br>82.7 [15.4]	132<br>78.9 [15.9]		
**6 months**	N<br>Mean (SD)	124<br>83 [16.2]	124<br>82.2 [14]		
**9 months**	N<br>Mean (SD)	125<br>84.2 [15]	117<br>82.9 [15.5]		
**12 months**	N<br>Mean (SD)	124<br>85.1 [15.3]	124<br>81.9 [16.9]		

PedsQL Domain Scores: 0=worst 100=best.

Means and standard deviations are presented unless otherwise specified.

A positive mean difference favours the Prednisolone Arm.

* Adjusting for the background therapy at baseline and baseline value.

© 2021 Christian MT et al. *JAMA Surgery*.

eTable 5. Rationalisation of Ethnic Groups Following Self-reported Ethnicity

Ethnic broad group	N (%)		Self-reported Ethnicity	N (%)	
	Prednisolone	Placebo		Prednisolone	Placebo
White	96 (72%)	92 (67%)	British	85 (63%)	88 (64%)
			Irish	0 (0%)	2 (1%)
			Other White background	11 (8%)	2 (1%)
South Asian	30 (22%)	28 (20%)	Indian	9 (7%)	7 (5%)
			Pakistani	9 (7%)	9 (7%)
			Bangladeshi	4 (3%)	4 (3%)
			Sri Lankan	2 (1%)	1 (1%)
			Other Asian background	3 (2%)	2 (1%)
			White and Asian	3 (2%)	5 (4%)
Other ethnicity/unknown	8 (6%)	17 (12%)	African	1 (1%)	3 (2%)
			Other Black British background	1 (1%)	2 (1%)
			White and Black Caribbean	0 (0%)	2 (1%)
			White and Black African	1 (1%)	4 (3%)
			Other mixed background	1 (1%)	2 (1%)
			Other ethnic group	2 (1%)	3 (2%)
			Not stated	2 (1%)	1 (1%)

© 2021 Christian MT et al. *JAMA Surgery*.

© 2021 Christian MT et al. *JAMA Surgery*.

eFigure. Subgroup Analyses for Primary Outcome Measure

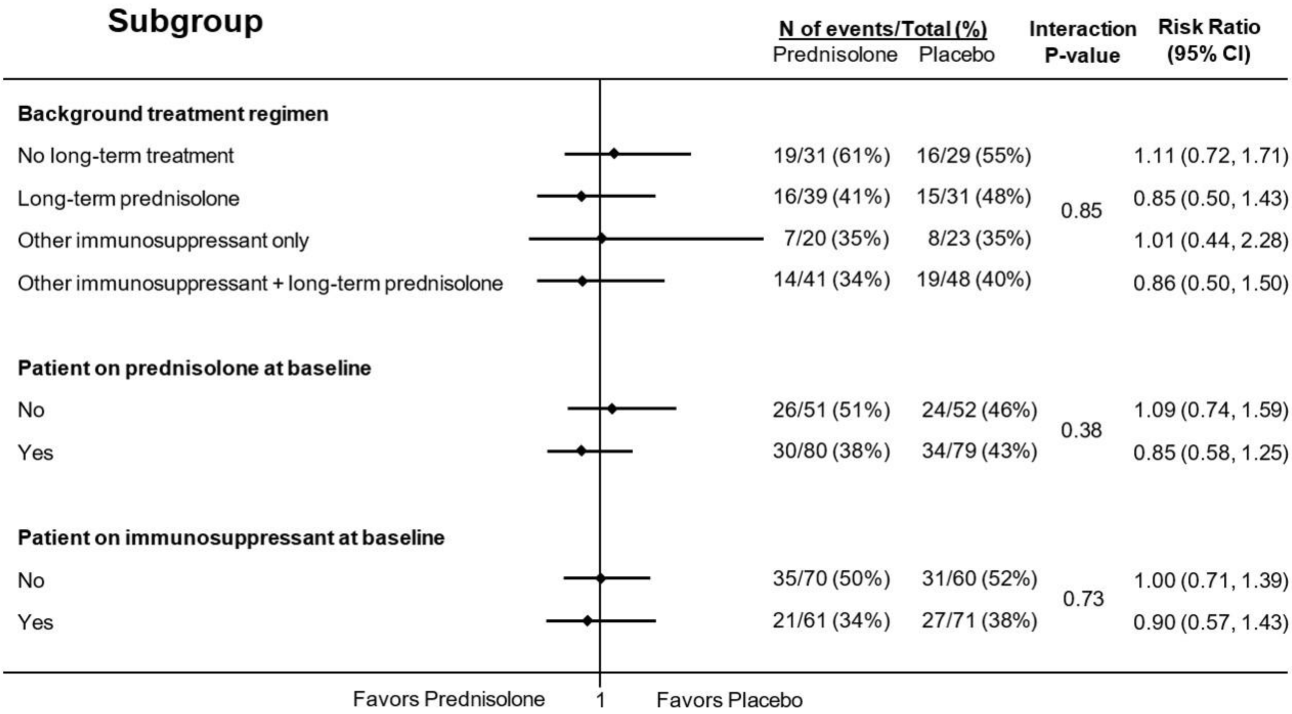

A risk ratio less than 1 favors the Prednisolone Arm

Adjusting for the background therapy at baseline, and including a treatment by subgroup interaction variable in the model.

© 2021 Christian MT et al. *JAMA Surgery*.
