# Supplementary material for: Evaluation of Daily Low-Dose Prednisolone During Upper Respiratory Tract Infection to Prevent Relapse in Children With Relapsing Steroid-Sensitive Nephrotic Syndrome: The PREDNOS 2 Randomized Clinical Trial
Source: JAMA Pediatr. 2021 Dec 20;176(3):1–8. doi: 10.1001/jamapediatrics.2021.5189 (PMC8689426; doi:10.1001/jamapediatrics.2021.5189)
Supplement: Supplement 3. — Data Sharing Statement [file jamapediatr-e215189-s003.pdf]

Data Sharing Statement

Christian. Evaluation of Daily Low-Dose Prednisolone During Upper Respiratory Tract Infection With Relapse in Children With Relapsing Steroid-Sensitive Nephrotic Syndrome. *JAMA Pediatr*. Published December 20, 2021. doi:10.1001/jamapediatrics.2021.5189

Data
Data available: Yes
Data types: Deidentified participant data
How to access data: martin.christian@nuh.nhs.uk
When available: With publication

Supporting Documents
Document types: None

Additional Information
Who can access the data: Data will be available to researchers with a clear research plan and hypothesis, with the appropriate team in place to undertake the work.
Types of analyses: For any appropriate purpose as detailed in a research plan.
Mechanisms of data availability: Requests for access to data from the PREDNOS 2 trial should be addressed to the corresponding author at martin.christian@nuh.nhs.uk. The individual participant data collected during the trial (including the data dictionary) will be available, after de-identification, when the article has been published with no end date. All proposals requesting data access will need to have a research plan and specify how the data will be used, and all proposals will need the approval of the trial co-investigator team (or individual(s) subsequently delegated this responsibility) before data release.
